# Global controls on phosphatization of fossils during the Toarcian Oceanic Anoxic Event

**DOI:** 10.1038/s41598-021-03482-7

**Published:** 2021-12-16

**Authors:** Sinjini Sinha, A. D. Muscente, James D. Schiffbauer, Matt Williams, Günter Schweigert, Rowan C. Martindale

**Affiliations:** 1 Department of Geological Sciences, The University of Texas at Austin, 2275 Speedway, Austin, TX 78712 USA; 2 Department of Geology, Cornell College, 600 First Street SW, Mount Vernon, Iowa, 52314 USA; 3 Department of Geological Sciences, University of Missouri, 101 Geological Sciences Building, Columbia, MO 65211 USA; 4 X-Ray Microanalysis Core Facility, University of Missouri, 1 Geological Sciences Building, Columbia, MO 65211 USA; 5 Bath Royal Literary and Scientific Institution, 16–18 Queen Square, Bath, BA1 2HN UK; 6 Staatliches Museum für Naturkunde, Rosenstein 1, 70191 Stuttgart, Germany

**Keywords:** Palaeontology, Biodiversity, Biogeochemistry, Biogeography, Biodiversity, Biogeochemistry, Biogeography, Palaeoecology

## Abstract

Konservat-Lagerstätten—deposits with exceptionally preserved fossils—vary in abundance across geographic and stratigraphic space due to paleoenvironmental heterogeneity. While oceanic anoxic events (OAEs) may have promoted preservation of marine lagerstätten, the environmental controls on their taphonomy remain unclear. Here, we provide new data on the mineralization of fossils in three Lower Jurassic Lagerstätten—Strawberry Bank (UK), Ya Ha Tinda (Canada), and Posidonia Shale (Germany) —and test the hypothesis that they were preserved under similar conditions. Biostratigraphy indicates that all three Lagerstätten were deposited during the Toarcian OAE (TOAE), and scanning electron microscopy (SEM) and energy dispersive X-ray spectroscopy (EDS) show that each deposit contains a variety of taxa preserved as phosphatized skeletons and tissues. Thus, despite their geographic and paleoenvironmental differences, all of these Lagerstätten were deposited in settings conducive to phosphatization, indicating that the TOAE fostered exceptional preservation in marine settings around the world. Phosphatization may have been fueled by phosphate delivery from climatically-driven sea level change and continental weathering, with anoxic basins acting as phosphorus traps.

## Introduction

Konservat-Lagerstätten**—**exceptional deposits that contain fossils of organisms with weakly or non-biomineralized tissues and articulated skeletons**—**are rare but provide unique insights into past life, including information about organism morphology, ecology, diversity, paleocommunity structure, and tissues not typically preserved e.g.^1–3^. They form under circumstances where soft-tissues are mineralized faster than they are degraded^4^, and notably vary in abundance and facies across geographic and stratigraphic space, suggesting that their preservation potential depends on both overarching global controls and local variability in paleoenvironmental conditions^4–6^. Ocean redox conditions may represent a control on the exceptional fossil record. Most marine Lagerstätten were preserved prior to the middle Paleozoic, when oceanic oxygen levels were low, and exceptional preservation was comparatively rare in younger marine settings^3,6^, except during Jurassic and Cretaceous Oceanic Anoxic Events (OAEs)^7–15^. Although water column anoxia or hypoxia may promote preservation of soft tissues by limiting scavenging and bioturbation^4^, some Lagerstätten contain evidence of benthic life at various levels^9,16^. Furthermore, anaerobic processes can degrade soft-tissues as rapidly as aerobic decay^17^, and anoxic conditions can limit fossil mineralization^18^. For these reasons, the effects of water column anoxia on exceptional preservation remain debated.

Toarcian Lagerstätten provide an opportunity to explore the effects of an OAE on exceptional preservation in disparate settings. The Early Jurassic was an interval of significant environmental changes and biotic crises, including the ~ 183 Ma Toarcian Oceanic Anoxic Event (TOAE) e.g.^19^. The Karoo-Ferrar Large Igneous Provinces injected greenhouse gases into the atmosphere, causing carbon dioxide levels to increase from ~ 500 ppmv to ~ 1000 ppmv during the TOAE and subsequent global warming, with estimated temperature increases of 2–3.5 °C in the subtropics and 6–8 °C at higher latitudes^20–24^ Increased temperature and humidity exacerbated hydrological cycles, which led to high precipitation and continental runoff e.g. ^25,26^. Excessive nutrients in the ocean increased primary productivity, resulting in low oxygen conditions, global extinctions e.g.^19,27,28^, and organic-rich black shale deposition^25,26^.

**Figure 1:**
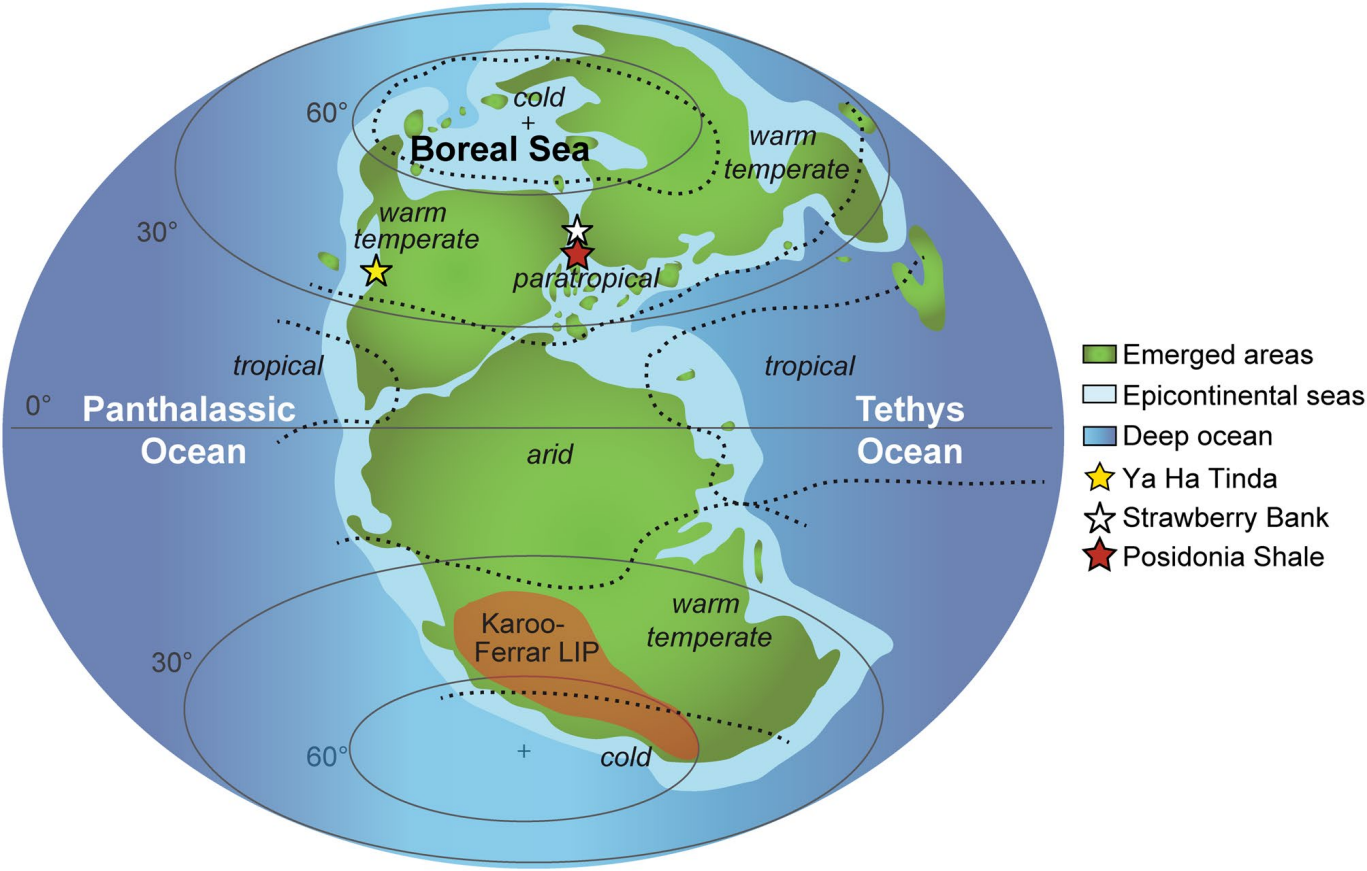
Paleogeographical map showing the location of the three Toarcian Lagerstätten, map based on Dera et al.^75^ and Fantasia et al.^76^.

**Figure 2:**
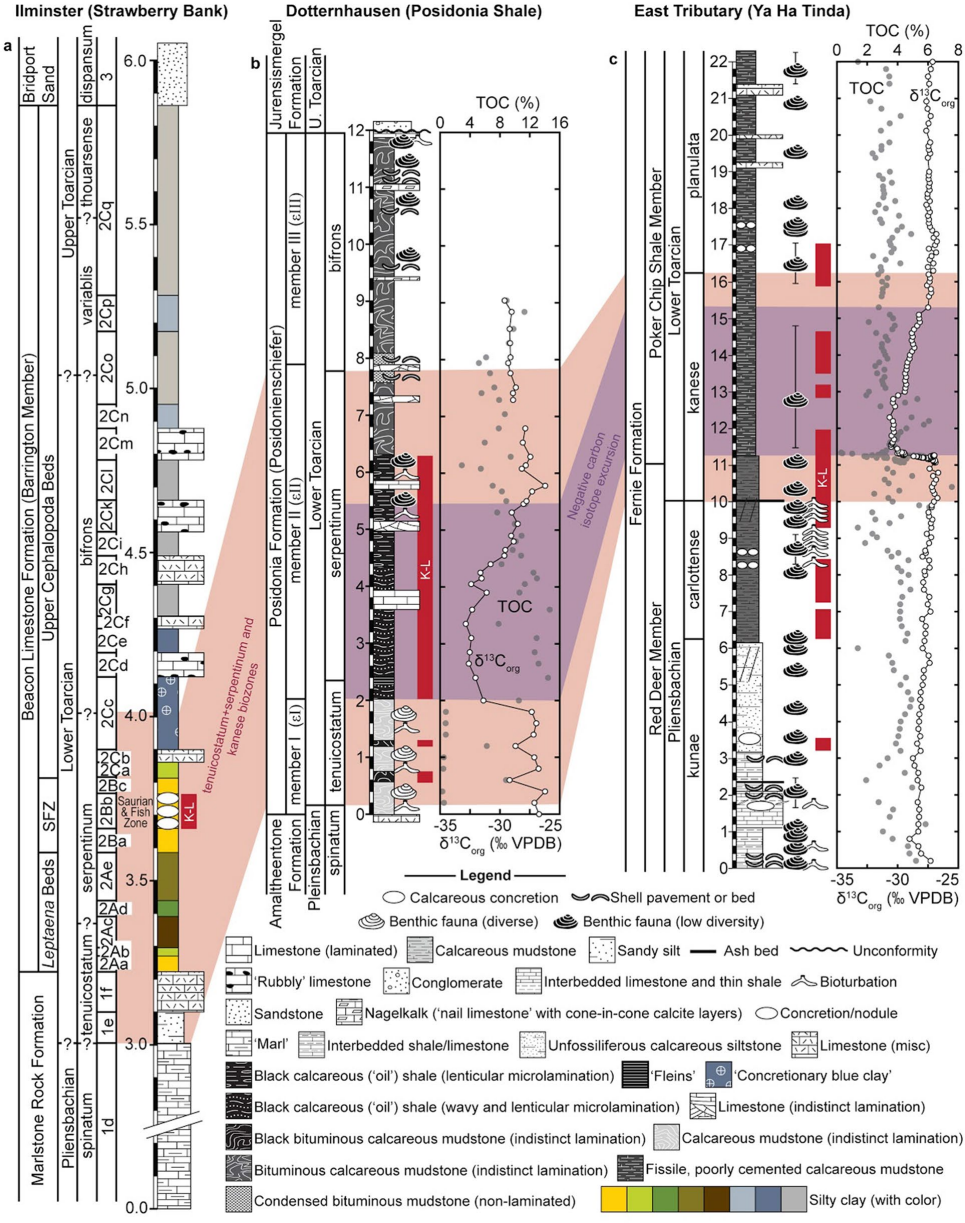
Stratigraphy of three sections with exposures of Lower Jurassic (Toarcian) Konservat-Lagerstätten (K-L). (**a**) Strawberry Bank (Ilminster, UK)^36^. (**b**) Dotternhausen (Baden-Württemberg, Germany)^16^. (**c**) East Tributary near Ya Ha Tinda Ranch (Alberta, Canada)^9^. Each section includes a sedimentary log along with names of lithostratigraphic (e.g. formations, members, beds, etc.), chronostratigraphic (sub-stage), and biostratigraphic (fossil biozone and subzone) units. Chemostratigraphic data, in the form of stable carbon isotope (δ^13^C relative to the Vienna Pee Dee Belemnite) and total organic carbon (TOC) measurements, are provided with the Dotternhausen and East Tributary logs. The TOAE is correlated with the *tenuicostatum* and *serpentinum* biozones in Europe and the *kanense* biozone in North America. Shaded areas illustrate correlations between the biozones and negative carbon isotope excursion caused by the event. Locations of Konservat-Lagerstätten are illustrated by vertical red bars (labelled with “K-L”). Values in logs are provided at meter scale. “Mudstone” refers to terrigenous mudstone (rather than micrite).

The Strawberry Bank (UK), Posidonia Shale (Germany), and Ya Ha Tinda (Canada) Lagerstätten (Fig. 1) all occur within the *Dactylioceras tenuicostatum*, *Harpoceras serpentinum*, *Hildoceras bifrons*, and equivalent (*Dactylioceras kanense* and *Rarenodia planulata*) biozones of the early Toarcian and exhibit signs of the negative isotope excursion that is a signature of the TOAE^9,10,15^ (Fig. 2). The Strawberry Bank Lagerstätte (Beacon Limestone Fm.) is interpreted as a shallow marine lagoon deposit^10^, while both the Ya Ha Tinda (Fernie Fm.) and Posidonia Shale (Posidonienschiefer Fm.) Lagerstätten record deeper marine settings, which intermittently developed bottom water anoxia^29^ between times of oxic and suboxic conditions^9,16,30^. These three Lagerstätten have similar fauna (Fig. 4) including ichthyosaurs, ray-finned fishes, crinoids, coleoids (belemnites and vampyropods), ammonites, bivalves, and crustaceans^9,10,16,31^. In this contribution, we synthesize new and published data on Toarcian Lagerstätten taphonomy to test whether their modes of preservation are explained by local or global factors e.g.^16,18,32^. We evaluate a model for exceptional fossilization during OAEs^18^, which relates the origin of Lagerstätten to redox-dependent processes.

## Geologic Setting

### Strawberry Bank Lagerstätte

The Strawberry Bank Lagerstätte is part of the Lower Jurassic Lias Group of sedimentary rocks, which extends from Dorset to Yorkshire in the United Kingdom and was deposited in the Jurassic epicontinental sea on the northwestern margin of the Tethys Ocean^10,33^. This Lagerstätte is located near the town of Ilminster, Somerset, and belongs to the Beacon Limestone Formation (Fig. 2). This formation exhibits spatial heterogeneity in its lithology; along the coast of Dorset, it consists of well cemented limestones, but the clay content increases towards the north^34^. Consequently in Somerset, the Beacon Limestone Formation typically consists of interbedded marls and argillaceous limestones^34,35^, and at Strawberry Bank, it is composed of interbedded nodular limestones and silty-clays^10,36^. The Beacon Limestone Formation lies above the Marlstone Rock Formation and below the Bridport Sand Formation^35^, and according to ammonite biostratigraphy, represents the lower to upper Toarcian interval, *falciferum* biozone (Fig. 2).

Most of the fossils from this Lagerstätte were collected between 1840 and 1860 by Charles Moore and the original quarry has been backfilled and converted to farmland^10^. Despite the extensive collections, few studies were conducted on the fossils reported by Moore^37–41^, but recent research has focused on the vertebrate taxa, such as the crocodiles, ichthyosaurs, and fish^10,42–44^. Strawberry Bank fossils include cephalopods, crustaceans, and insects, as well as articulated fishes and reptiles; some fossils include remains of delicate structures such as coleoid ink sacs^10^. Most (if not all) of these fossils were preserved in carbonate concretions or nodules and collected from the ‘Saurian and Fish Zone,’ which is either a nodular limestone bed or thin unit of clay (Fig. 2)^36^. In either case, this stratum has been correlated with the *exaratum* subzone of the *falciferum* (now *serpentinum*) ammonite biozone^10^, which in other locations, is corelated with the extreme negative values of the carbon isotope excursion caused by the TOAE^45^. Cephalopod and crustacean fossils support the interpretation that the nodular limestone was deposited in a setting influenced by marine life and processes. The abundance of insect fossils and relatively high clay content of the Beacon Limestone Formation at Ilminster suggest that the fossils were preserved in a transitional depositional environment near the paleocoastline^10^. Given that exceptional fossil preservation often occurs in restricted environments^6^ and the Beacon Limestone Formation exhibits great lithologic variation in Somerset, the Lagerstätte may have formed within a localized lagoon, mud flat, or similar quiet-water environment.

### Posidonia Shale Lagerstätte

The Posidonienschiefer Formation is a geologic unit located in numerous countries across Europe^46^, most notably (for exceptionally preserved fossils) from the Swabian Alb (Germany) and Switzerland (as the Rietheim Member of the Staffelegg Formation), the Franconian Alb (Mistelgau and Bad Staffelstein), and Lower Saxony (Schandelah). In general, this unit consists of black, microlaminated shales and mudstones intercalated with limestones^15,16,45,47^, which were deposited in the European epicontinental sea that existed during the Mesozoic^16,30,48^ (Fig. 2). Historically, in the Swabian Alb, the Posidonienschiefer Formation has been utilized as a source of construction and building materials as well as fuel. Quarrying of the raw materials in places, such as Holzmaden, Ohmden, and Dotternhausen, over the past 200 years has led to the discovery and collection of a myriad of exceptionally preserved fossils, which are collectively known as the ‘Posidonia Shale Lagerstätte^47^.

**Figure 3:**
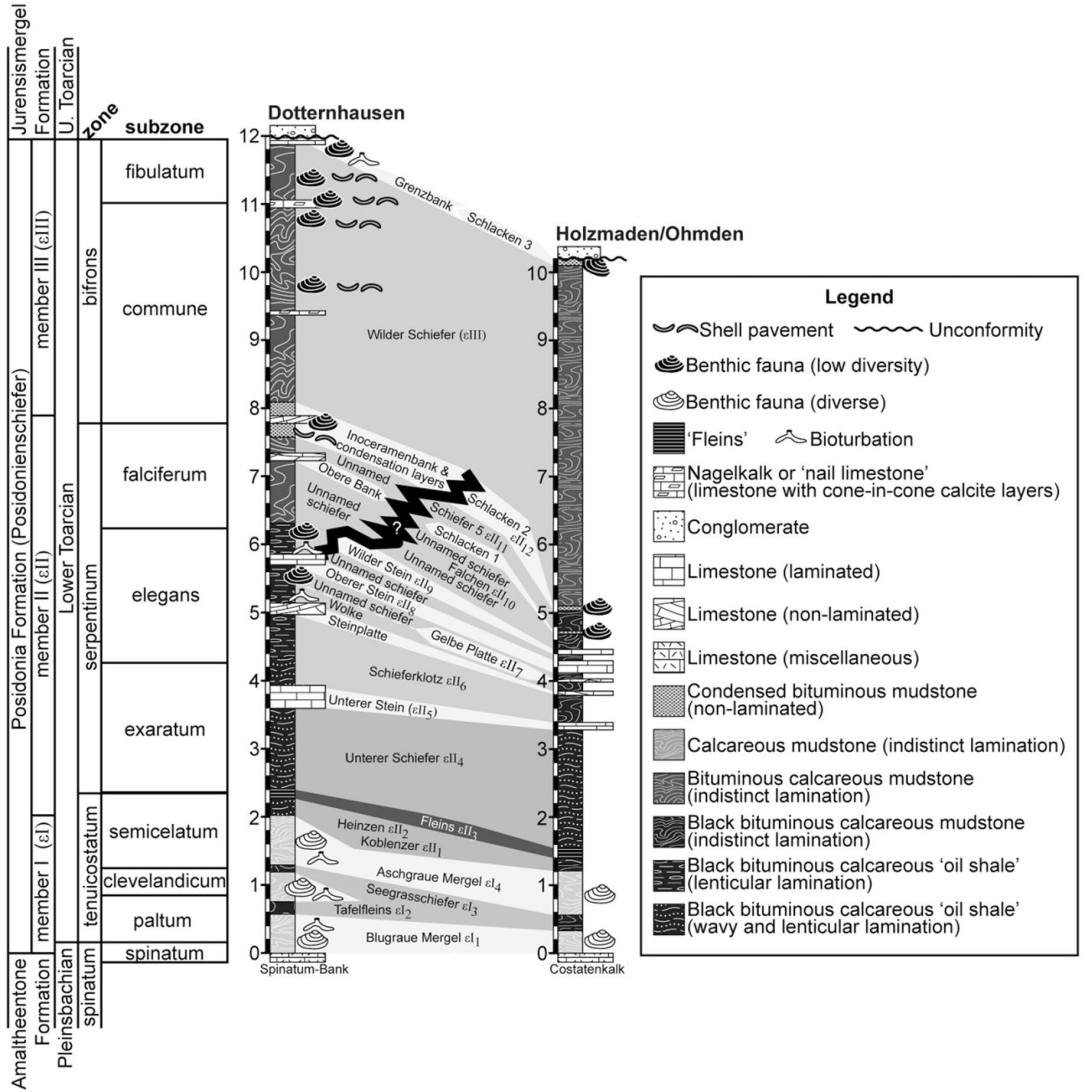
Stratigraphy of the Posidonia Shale at Dotternhausen, Dormettingen, Holzmaden, and Ohmden. Sedimentary log for Dotternhausen (and nearby Dormettingen)^16^. The other log illustrates the stratigraphy in the area between Ohmden and Holzmaden, as described by Hauff ^47^, with thicknesses corresponding to their average values between Reutlingen and Schwäbisch Gmünd. These logs are provided with lithostratigraphic (formation and member), chronostratigraphic (sub-stage), and biostratigraphic (fossil biozone and subzones) units^16^.

The Posidonia Shale lies above the Amaltheenton Formation and below the Jurensismergel Formation in the Swabian Alb, where it is informally divided into three members^45^—member I (εI), member II (εII), and member III (εIII)—which are further subdivided into beds and intervals that can be correlated across the region to varying degrees^47^ (Fig. 3). Although the exact stratigraphy can vary from one locality to the next, some lithologic patterns are evident in all sections (Fig. 3). In general, Member I corresponds to non-laminated terrigenous (but also calcareous) mudstones (‘marls’) with diverse benthic fossils and robust evidence of bioturbation; Member II—the TOAE interval—consists of black, microlaminated, bituminous ‘oil’ shales that contain a wealth of exceptionally preserved fossils but few benthic body or trace fossils; and Member III refers to the Wilder Schiefer, another black shale unit but one with shell beds, bioturbation, and other robust evidence of benthic life^15,16^. These broad lithologic trends follow patterns of change in the redox conditions of seafloor environments during the TOAE.

The lowest member (Member I) generally consists of two or more intervals of non-laminated and highly bioturbated mudstone (e.g. the ‘Blaugraue Mergel’ and ‘Aschgraue Mergel’) separated by one or two layers of relatively hard yet bituminous black shale (e.g. ‘Tafelfleins’ and ‘Seegrassschiefer’), which are inconsistently laminated. The mudstones encompass a diversity of benthic body and trace fossils, whereas the shales contain few benthic body fossils and ichnofossils other than the subhorizontal branching burrow system, *Phymatoderma granulatum*. The middle Posidonia Shale is dominated by black, calcareous, bituminous ‘oil’ shale intercalated with limestone layers. Beginning at the base of Member II, important shale units include the Koblenzer, Hainzen, Fleins, and Unterer Schiefer layers (in ascending order). With the exception of the Koblenzer layer, which is inconsistently laminated and contains faint evidence of bioturbation^46^, all of these units are microlaminated black shales with abundant but non-diverse benthic fauna^16^, including exceptionally preserved fossils^47^. The shales located higher in the middle member contain a number of limestone layers that are not present at all sites^45^. The lowermost of these limestone layers—the Unterer Stein—is a well-laminated and laterally extensive limestone, which serves as a marker bed for regional lithostratigraphic correlation^45,46^. The thin shale unit above the Unterer Stein—the Schieferklotz— is known for marine reptiles^16^. Above this layer, shales are interbedded with the following limestone layers (in ascending order): the Steinplatte, a non-laminated limestone that sometimes appears yellow in outcrop due to weathering; the Gelbe Platte, a similar limestone bed that is missing at some localities; and the Oberer Stein, another well-laminated and laterally extensive limestone that serves as a regional marker^45,46^. Above the Oberer Stein, the Posidonienschiefer exhibits a significant amount of regional variation in its lithology^16,46^. In general, the upper part of Member II consists of bituminous shales and mudstones intercalated with localized layers of limestone^16,46^. Notably, many of these shales and mudstones are irregularly laminated, show evidence of bioturbation, and include fossils of diverse benthic fauna. A number of these beds also exhibit evidence of sediment starvation and sedimentary condensation, such as ‘schlacken’ or time averaged accumulations of mollusk and vertebrate debris (e.g. fish scales and teeth)^16^.

Member III corresponds to the Wilder Schiefer, another unit of non-laminated calcareous mudstone and shale. It generally contains greater amounts of carbonate and lesser amounts of organic matter than the more bituminous rocks in the lower part of Member II^16^. Shell beds, schlacken, bioturbation, and assemblages of diverse benthic fauna are relatively common in the Wilder Schiefer^46^.

Given this succession of facies and the types of fossils present in the Posidonia Shale Lagerstätte, the organisms were most likely preserved in the deep water of an anoxic, stagnant marine basin during a slow transgression^48^. Fish, marine reptiles, arthropods, echinoderms, bivalves, gastropods, ammonites, belemnites, and other coleoids occur through the succession; however, most of the exceptionally preserved fossils occur between the Koblenzer (Supplementary Figs. S60–S66) and Oberer Stein layers; for example, in the Fleins (Supplementary Figs. S45–S47) and Unterer Schiefer layers (Supplementary Figs. S50–S56) (Fig. 3). This interval spans from the top of the *tenuicostatum* biozone (lower Posidonia Shale) to the middle of the *falciferum/serpentinum* biozone (middle Posidonia Shale), and encompasses the negative carbon isotope excursion that was produced by the TOAE^48^.

The oxygenation history of the Posidonia Shale is complex; high-resolution studies of fauna, total organic content (TOC), and sulfur levels at Dotternhausen indicate long-term anoxia/dysoxia with periods of oxygenation, which lasted for weeks to several years^16,48^. In addition, the presence of pyrite framboids are indicative of anoxic conditions in the water column and/or sediment pore water (Supplementary Figs. S44, S53). Geochemical data from iron and thallium redox proxies also indicated that the Posidonia Lagerstätte was deposited under anoxic to euxinic conditions^29^, although it should be noted that several bioturbated layers and benthic colonization events are found within these units, suggesting intervals of oxia^16,48^. The source and variation in oxygen could have been a result of atmospheric circulation and seasonal changes in wind patterns^48,49^.

### Ya Ha Tinda Lagerstätte

The Ya Ha Tinda Lagerstätte belongs to the Red Deer and Poker Chip Shale members of the Fernie Formation e.g.^50^ with most specimens collected from the Ya Ha Tinda Ranch in Alberta, Canada^9^. In the area around Ya Ha Tinda Ranch, the Red Deer Member overlies rocks of the Middle Triassic Sulphur Mountain Formation, and consists of grey to black platy calcareous shale interbedded with fine siltstone and fetid black limestone^50,51^. The overlying lower Toarcian Poker Chip Shale consists of finer-grained and predominantly poorly-cemented fissile black, calcareous shales and mudstone^29,52^. The Poker Chip Shale is overlain by the Highland Member of the Fernie Formation^51,53^. The predominance of finely laminated rocks lacking major sedimentary structures^54^ suggest that they were deposited below fair weather wave base in a deep-water, open marine setting^55^.

According to ammonite biostratigraphy, the Red Deer Member spans the *kunae*, *carlottense* and part of the *kanense* biozones of the late Pliensbachian and lowermost Toarcian (equivalent to the *margaritatus*, *spinatum*, and *tenuicostatum* biozones in the European sub-boreal ammonite zones) and the Poker Chip Shale was deposited in the *kanense* and *planulata* zones of the later Toarcian (equivalent to *falciferum/serpentinum* and *bifrons* biozones of the European sub-boreal ammonite zones)^52^. Exceptionally preserved fossils occur throughout this interval at Ya Ha Tinda^9,18^.The Pliensbachian/Toarcian boundary occurs within the Red Deer Member, and is located 1 m below its contact with the Poker Chip Shale^52^. Chemostratigraphic data places the TOAE within the Poker Chip Shale Member, with the onset of the carbon isotope excursion just at the end of the Red Deer Member^52^.

The Red Deer and Poker Chip Shale members contain fish^56^, ichthyosaurs^53^, crinoids^57^, crustaceans^58,59^, brachiopods, coccolithophores, trace fossils, bivalves, gastropods, ammonites^53,60^, and coleoids like vampyropods^61,62^. Exceptionally preserved fossils include crustacean (shrimp and lobster) cuticles^58,59^; coleoid gladii with ink sacs and mantle muscle tissues^61,62^; crinoid calyxes^57^, articulated fish^56^, and ichthyosaurs^53^. Like the Posidonia Shale, geochemical data (iron and thallium redox proxies) suggest that parts of the Ya Ha Tinda Lagerstätte were deposited under anoxic and even euxinic conditions^29^. Nevertheless, it is important to note that several well bioturbated layers, benthic colonization events^60^, and phosphatized fossil specimens^18^ are also found within these units, which indicate that intervals of oxygenation were rare to common throughout the depositional history of the succession.

## Materials and methods

**Figure 4:**
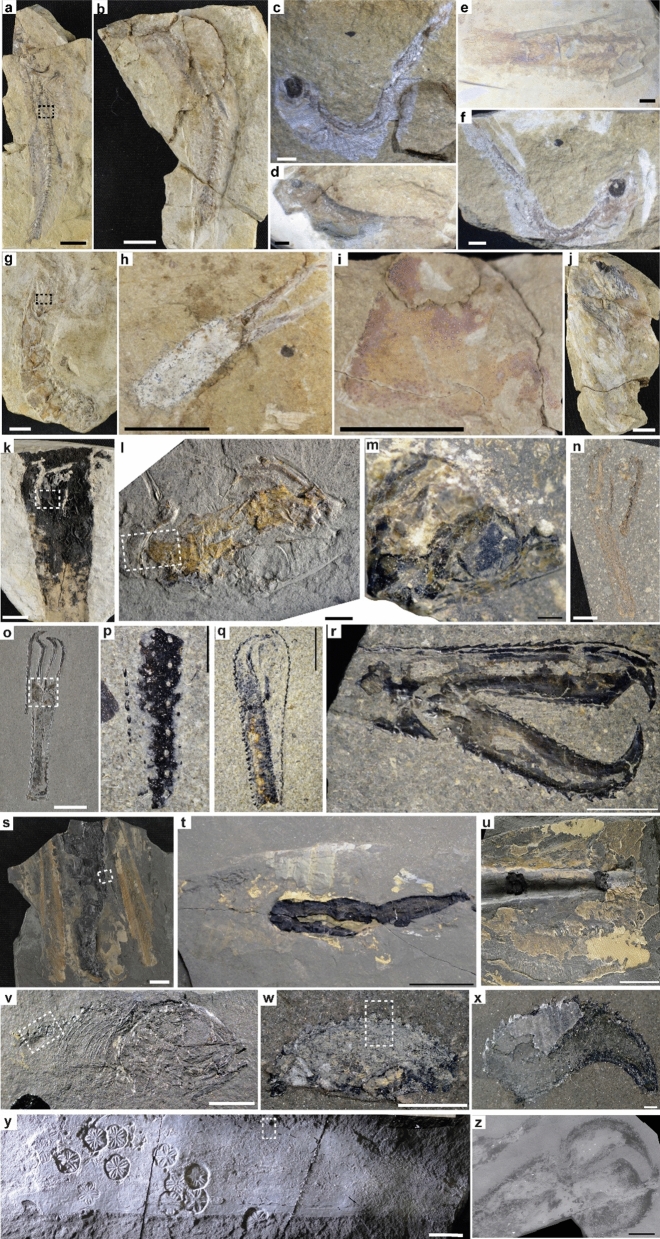
Fossil specimens from Strawberry Bank (**a**)–(**k**), Posidonia Shale (l-u), and Ya Ha Tinda (v-z). Boxes indicate regions of interest shown in Fig. 5. (**a**)–(**f**) Articulated fish *Leptolepis* (BRLSI.M1261, BRLSI.M1261A, BRLSI.M1269A, BRLSI.M1271A, BRLSI.M1275, and BRLSI.M1269) with well-preserved vertebral column. (**g**) Crustacean (BRLSI.M1256) with abdomen (tail). (**h**)**-**(**i**) Crustacean (BRLSI.M1242 and M1243C) with well-preserved exoskeleton. (**j**) Coleoid gladius (BRLSI.M1237 B). (**k**) Vampyropod (BRLSI.M3917) with well-preserved ink sac (black material) and mantle tissue (white material). (**l**) Fish fossil *Leptolepis* (NPL00036036.000, Kromer Quarry). (**m**) Fish skull (NPL00094461.000, Dormettingen near Dotternhausen). (**n**) claw of an *Uncina posidoniae* (NPL00036039.000) **o**, Chela of the first pereiopod (claw) of an *Uncina posidoniae* (NPL00036038.000) lobster. (**p**)–(**r**), exoskeletons of crustaceans (NPL00094458.000, NPL00094459.000, and NPL00094457.000). (**s**) Vampyropod *Loligosepia aalensis* (NPL00036037.000) gladius (tan material) with well-preserved ink sac (black material). (**t**) Vampyropod *Clarkeiteuthis* sp. (NPL00094460.000) gladius with well preserved ink sac. (**u**) Vampyropod *Loligosepia* sp. gladius (NPL00036035.000) with pieces of ink sac. (**v**) Articulated fish (TMP2014.021.0043) with fins and gill arches. (**w**, **x**) Chela of the first pereiopod (claw) of an *Uncina pacifica* lobsters (TMP2018.024.0030 and TMP2018.024.0037). (**y**) Coleoid *Paraplesioteuthis* cf. *sagittate* (TMP2005.028.0001) gladius with impressions of *Seirocrinus subangularis* (crinoid) ossicles. **z**, Claw of the holotype specimen of the decapod crustacean *Uncina pacifica* (TMP2002.043.0005). Scales, (**a**), (**b**), (**g**), (**j**), (**k**), (**n**), (**o**), (**s**), (**t**), (**u**), (**y**) = 1 cm; (**c**)–(**f**), (**l**), (**p**) = 2 mm; (**h**), (**i**) = 0.5 cm; (**m**), (**w**), (**x**) = 1 mm; and **q**, **r**, **v**, **z** = 5 mm.

To assess the similarity of the Lagerstätten with respect to their preservational pathways, we examined multiple exceptionally preserved fossils (Table 1) from each Lagerstätte with scanning electron microscopy (SEM) and analyzed their elemental compositions with energy dispersive X-ray spectroscopy (EDS)^63^. See supplementary information for detailed methods. Material from the Strawberry Bank, Posidonia Shale, and Ya Ha Tinda deposits are reposited at Bath Royal Literary and Scientific Institution (BRLSI), Bath, UK; Non-vertebrate Paleontology Laboratory (NPL), University of Texas at Austin, USA; and Royal Tyrrell Museum of Palaeontology (RTMP), Alberta, Canada, respectively. Fossils analyzed include articulated teleost fish, crustacean carapaces, and coleoid gladii and soft tissues from all deposits (Fig. 4; Supplementary Table S1).

**Table 1:** Number of specimens analyzed (number in parentheses indicates the number of specimens figured in the article).

Specimen	Posidonia Shale	Strawberry Bank	Ya Ha Tinda
Fish	2 (2)	4 (4)	4 (1)
Crustacean	11 (5)	5 (5)	6 (2)
Coleoid	6 (4)	2 (2)	3 (1)

## Results

### Strawberry Bank Lagerstätte, Ilminster, UK

Strawberry Bank fossils primarily occur in limestone concretions (e.g. Fig. 4 a-d; Supplementary Figs. S1–S13). The fossils assessed are mostly flat with minor μm- to mm-scale topography (e.g. Supplementary Figs. S1–S33). Elemental maps show fish skeletons, crustacean carapaces, and coleoid gladii (Fig. 5) contain high concentrations of P and Ca, indicating that they consist of apatite minerals; additional images highlighting these findings are displayed in Supplementary Figs. S1–S18 (fish bones), S19-S33 (crustacean carapaces), and S34-S39 (coleoid material). In addition, many fossils contain high amounts of S (e.g. Supplementary Figs. S2–S8, S1–S33, S18–S21, S23–S24, S28, S30; S34–S39), traces of F (Supplementary Figs. S1–S5, S31–S33), and coleoid soft tissues, such as ink sacs, contain high concentrations of C (Fig. 5, Supplementary Figs. S34–S36). The matrix surrounding the fossils contains appreciable Al and Si, suggesting siliciclastic material (Fig. 5), and traces of Fe and Ba (Supplementary Fig. S2, S10, S35). Auxiliary minerals, such as calcite and aluminosilicate minerals, are present but they occur in the matrix (Supplementary Figs. S1–S4, S10, S19–S20, S30–S31) and do not represent fossil remains.

**Figure 5:**
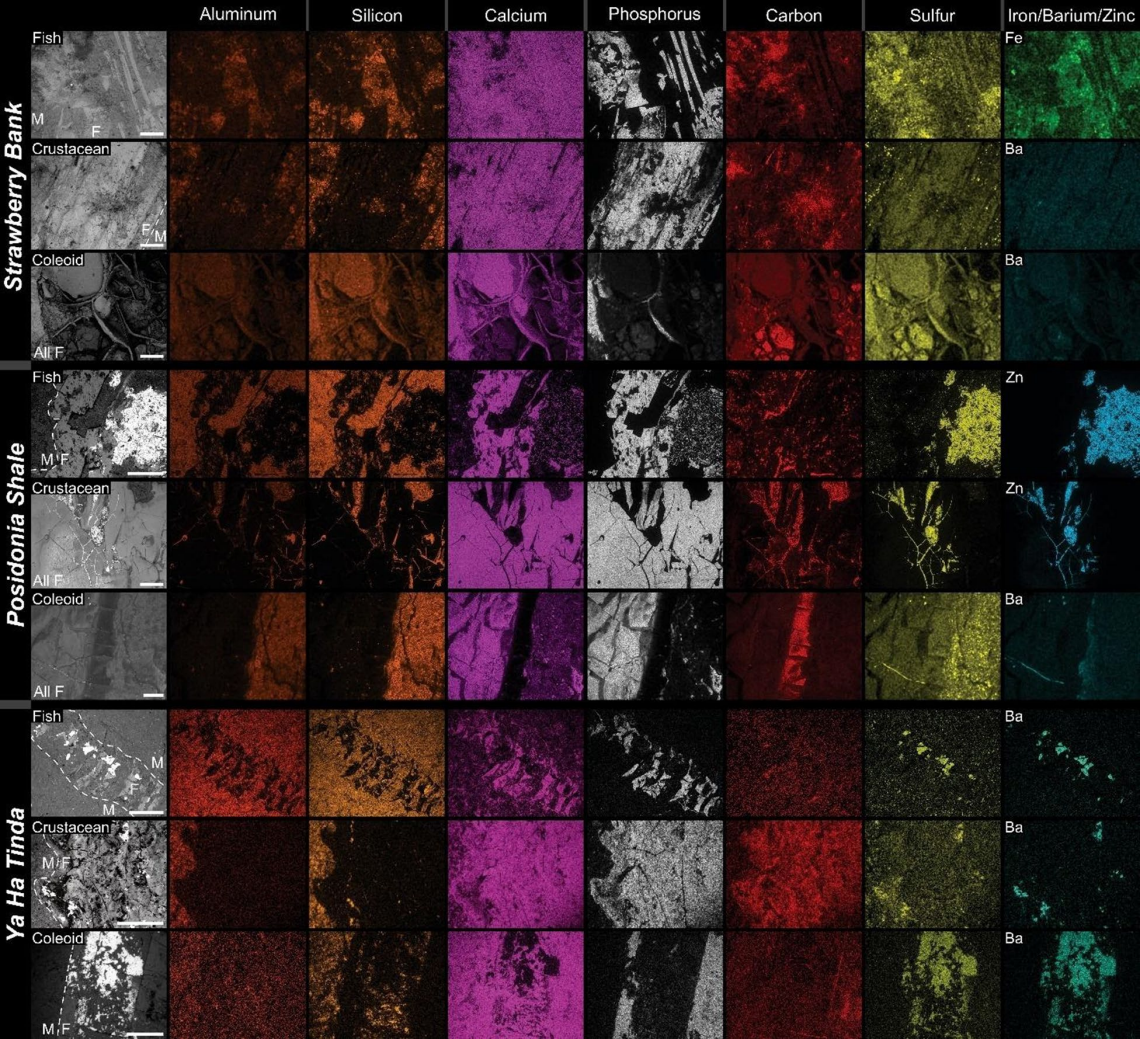
EDS elemental maps of specimens (areas from Fig. 4) from Strawberry Bank (SB), Posidonia Shale (PS), and Ya Ha Tinda (YHT) Lagerstätten fossils. Top to bottom- SB Fish, Phosphatized gut of *Leptolepis* (M1261; Fig. 4a). SB Crustacean, phosphatized exoskeleton of crustacean (M1256; Fig. 4g). SB Coleoid, phosphatized mantle and carbonaceous ink sac of vampyropod gladius (M3917; Fig. 4k) and alumino-silicate rich matrix. PS Fish, Phosphatized fish bone (NPL00036036.000; Fig. 4l). PS Crustacean, phosphatized *Uncina posidoniae* claw (NPL00036038.000; Fig. 4o). PS Coleoid, phosphatized mantle tissue of *Loligosepia aalensis* (NPL00036037.000; Fig. 4s) gladius with ink sac, surrounded by carbonaceous material. YHT Fish, Phosphatized skeletal material encrusted by barite (TMP2014.021.0043; Fig. 4v). YHT Crustacean, phosphatized claws of *Uncina pacifica* (TMP2018.024.0030; Fig. 4w) and carbonaceous material (encrusted by barite). YHT Coleoid, phosphatized gladius of *Paraplesioteuthis* cf. *sagittate* (TMP2005.028.0001; Fig. 4y), encrusted by calcite and barite*.* All scales = 1 mm. F = segments where fossil is present and M = segment where matrix is present.

### Posidonia Shale Lagerstätte, Swabian Alb, Europe (primarily Germany)

The Posidonia Shale is a finely laminated, bituminous black shale unit intercalated with limestones ^e.g.^^16,30,48^. Most fossils analyzed (Fig. 4l–u), even articulated fish skeletons and coleoid gladii, are flat with minor μm- to mm-scale topography (Supplementary Figs. S40–S44, S60–S65, S67–S78). Elemental maps, shown in Fig. 5, indicate that fish bones, crustacean carapaces, and coleoid gladii, ink sacs, and mantle tissues all contain higher concentrations of P and Ca (but lower concentrations of Al, K, Mg, Na, and Si) than the surrounding silicate matrix (Fig. 5). These results, indicate that the fossils largely consist of apatite minerals; additional images highlighting these findings are displayed in Supplementary Figs. S40–S44 (fish bones), S45–S59 (crustacean carapaces), S60–S63, S67, S69, S71–S73 (coleoid gladii), S63–S66, S68, S70, S74–S76 (coleoid ink sacs), and S77-S78 (coleoid mantle tissues). Phosphatic soft tissues were also found recently in ichthyosaur skin from the Fleins layer of the Posidonia Shale^64^. The data also indicate that most specimens contain a variety of auxiliary minerals: calcite, barite, pyrite, sphalerite, and aluminosilicate minerals. These minerals were formed after the soft tissues had been phosphatized, and do represent void-filling cements rather than mineralized soft tissues, even though many originally calcitic or aragonitic shell fossils in the Posidonia Shale consist of pyrite^65^. Indeed, the auxiliary minerals occur as cements that encrust phosphatic material (Supplementary Fig. S55, S62, S67) and fill cracks and voids within the fossils (Supplementary Figs. S42, S45, S48, S49, S52, S57, S58, S63–S66, S68). They are also present as cements and framboids within the host rock (Supplementary Fig. S44, S53). Some coleoid fossils contain small amounts of carbonaceous material (Supplementary Figs. S61–S66, S69–S70, S74), which occurs on phosphatic surfaces (Supplementary Figs. S61–S62, S67–S69). In some specimens, the carbonaceous material is localized to cracks and spaces (Supplementary Figs. S63–S66, S70) within the phosphatic fossils (Supplementary Fig. S66), which are also filled with diagenetic minerals (e.g. pyrite and barite).

### Ya Ha Tinda Lagerstätte, Alberta, Canada

The Ya Ha Tinda Lagerstätte spans parts of the Red Deer and Poker Chip Shale members of the Fernie Fm e.g.^50^. The Pliensbachian to early Toarcian Red Deer Member consists of grey to black platy calcareous shales interbedded with fine siltstones and fetid black limestones, whereas overlying Toarcian Poker Chip Shale is comprised of finer-grained and more fissile black, calcareous shales and mudstones^66^. The fossils (Fig. 4v–z) are generally flat with minor μm-to mm-scale topography (Supplementary Figs. S79–S82). Elemental maps, shown in Fig. 5, demonstrate, that fish skeletons, crustacean carapaces, and coleoid gladii, mantle tissues, and ink sacs contain higher concentrations of P, Ca, C, S, Ba, Zn, and Fe (but lower concentrations of Al, K, Mg, Na, and Si) than the silicate matrix (Fig. 5; Fig. 15 in Muscente et al.^18^). These data indicate that fossils consist of phosphatic and carbonaceous material as well as auxiliary minerals, including barite, calcite, pyrite, sphalerite, and aluminosilicate minerals^18^; additional images are displayed in Supplementary Figs. S79 (fish skeletons), S80-S81 (crustacean carapaces), and S82 (coleoid gladii, mantle tissues, and ink sacs). By and large, the exceptionally preserved organism consist of apatite minerals. Carbonaceous material is visible in backscattered electron (compositional) SEM imaging^63^, generally occurring as thin, dark (low average atomic number) layers on phosphatic material and as void fill within fossils^18^ (Supplementary Figs. S80, S82). The auxiliary minerals were formed after the soft tissues had been phosphatized, and now occurring as cements within secondarily phosphatized tissues (Supplementary Figs. S79, S81–S82).

For a summary of fossil mineralogy, see Table 2.

**Table 2:** Mineralogy of the analyzed specimens.

Lagerstätte	Organism							Matrix
	Fish			Crustacean	Coleoid			
	Eye	Bones (near eye, vertebral column, tail)	Gut	Carapace	Ink Sac	Mantle Muscle	Gladius	
Strawberry Bank	Alumino-silicates	Calcium phosphate rich in sulfur; traces of fluorine	Alumino-silicates	Calcium phosphate rich in sulfur; traces of fluorine	Carbonaceous	Calcium phosphate rich in sulfur	Calcium phosphate rich in sulfur	Alumino- silicates; calcite, traces of iron and barium
Posidonia Shale	N.A	Calcium phosphate	N.A	Calcium phosphate	Calcium phosphate	Calcium phosphate, barite, pyrite, trace of zinc (sphalerite)	Calcium phosphate	Pyrite, sphalerite, calcite, aluminosilicates, rare barite
Ya Ha Tinda	N.A	Calcium phosphate, carbonaceous	N.A	Calcium phosphate; carbon; iron; sulfur	Calcium phosphate, carbonaceous	Calcium phosphate; carbonaceous	Calcium phosphate, carbonaceous	Barite, calcite, alumino- silicates; rare pyrite and sphalerite

## Discussion

The three Toarcian Lagerstätten come from different regions (Tethys vs Panthalassa; Fig. 1), lithologies (shale vs lime muds), and depositional settings (deeper marine vs shallow lagoon). Despite these differences, they contain similar faunas e.g.^9,10,16,31^ (Fig. 4) and phosphatic fossils (Fig. 5). Although fish bones consist of bioapatite^67^, crustacean carapaces contain relatively small amounts of carbonate apatite^68^, and none of the vampyropod coleoid tissues^69,70^, or ichthyosaur skin or muscles^64^ contained apatite. Therefore, the fossils of crustacean carapaces, coleoid mantle tissues, and ichthyosaur skin and muscles provide robust evidence that the taphonomic pathways of the lagerstätten involved secondary phosphatization, or the conversion of organic substrates to apatite minerals^71,72^. This commonality shared by the three Lagerstätten, irrespective of depositional settings and proximity, supports the hypothesis that they reflect a broad common influence on their taphonomy: the TOAE. The auxiliary minerals, which distinguish the lagerstätten from each other (e.g. concretions at Strawberry Bank and pyrite in the Posidonia Shale), formed in response to local/regional processes, like sedimentation rate, pore water pH, sedimentary organic carbon content, reactive Fe (or Zn) availability, and sulfate levels^4,6,18,72^.

Other Toarcian Lagerstätten may provide additional evidence of phosphatization during the TOAE^7^. Muscente et al.^6^ recognized 11 potential Lagerstätten of Toarcian age. With one exception (the Zhargalant Formation Oshin-Boro-Udzur-Ula in Mongolia), which may be a terrestrial deposit, all of these 11 “Lagerstätten” belong to the Lias Group of Europe, and are represented almost entirely by insect fossils. By and large, these fossils are preserved in calcareous nodules/concretions within marine or restricted marine “clays” of limited exposure. Therefore, they resemble the Strawberry Bank in terms of geology and preservation^7^. Like Strawberry Bank, these other Lias deposits belong to the *tenuicostatum and serpentinum* biozones suggesting that they all occur within beds that can be correlated with the negative carbon isotope excursion of TOAE*.* Depending on the locality, the fossils may represent a single bed (like Strawberry Bank) or a range of strata (like the Posidonia Shale and Ya Ha Tinda deposits). Fossils at Grimmen, Dobbertin, Braunschweig, and other places have been reported from multiple sub-biozones (the *elegantulum* and *exaratum* subzones), suggesting that the exceptional preservation occurred throughout the duration of the event at these localities. In any case, although the taphonomy of these Lagerstätten have not received much attention, reports suggest that fossiliferous bedding planes such as those at Dobbertin are covered with phosphatized algae and that insect tissues at Gimmen have been replaced with apatite minerals.

**Figure 6:**
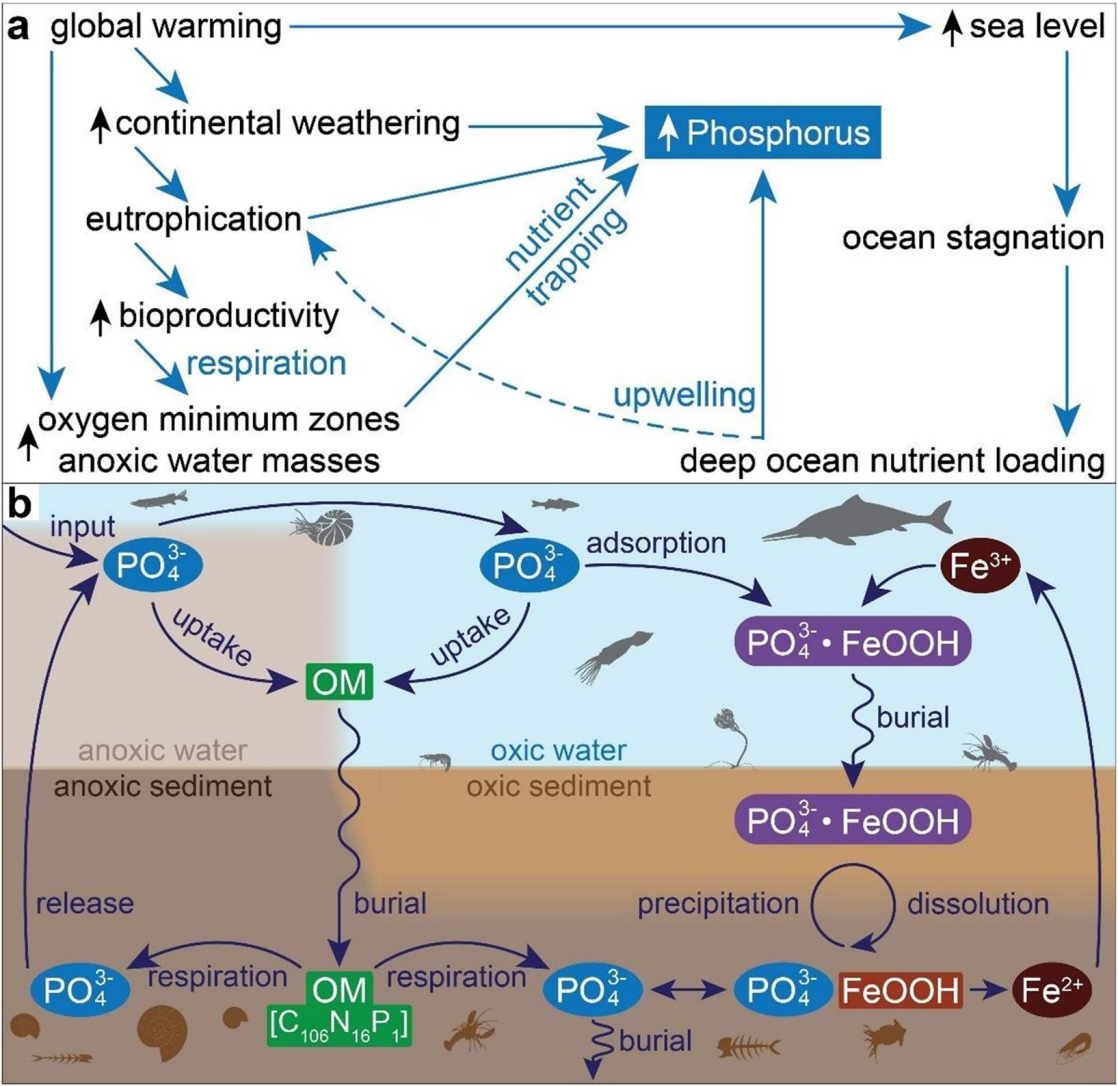
Proposed model of linkages between OAE-driven environmental changes and exceptional fossilization (see main text for details). (**a**) OAE-driven perturbations (e.g. warming and weathering) that lead to increased P availability and secondary phosphatization. (**b**) Possible cycling of P between sediments and the water column in the three Lagerstätten.

Phosphatization occurs in environments conducive to phosphogenesis, or precipitation of P as apatite minerals in sediment^73^. In marine environments, natural sources of P include nutrient runoff (weathering) and oceanic upwelling (Fig. 6). Phosphogenesis generally occurs in sediment with high phosphate content sourced by (i) basin-scale processes (e.g. anoxia and circulation) that influence P availability; (ii) burial of phosphate absorbed onto iron oxide particulates; (iii) remineralization of P from organic matter through microbial respiration; and (iv) other microbial activities (e.g. storage and release of polyphosphates) that influence porewater chemistry e.g.^18,73^. Phosphogenesis commonly results from remineralization via microbial sulfate reduction^74^ as well as from reduction of iron oxide particulates^73^. These particulates help to limit the efflux of phosphate from pore to bottom water via diffusion through cyclic ‘iron-pumping,’ wherein particulates absorb phosphate produced deeper in the sediment and prevent its escape^73^. Because sulfate and iron oxide reduction are redox-sensitive processes, phosphatization may be favored in marine environments with (dys)oxic bottom water^18^.

Our findings support the hypothesis that the TOAE favored exceptional preservation via phosphatization^18^. Although anoxic water masses did not directly cause exceptional preservation during the Early Jurassic, they set the stage by delaying carcass disarticulation, inhibiting soft-tissue decay, releasing P from sediments, enhancing P from upwelling and erosion, and/or trapping nutrients in the water columns of basins^18^ (Fig. 6). Under these conditions, exceptional fossils were likely preserved (1) at the boundaries between (sub)oxic and nutrient-trapping anoxic water bodies and (2) during ephemeral pulses of oxygenation in anoxic basins, when the foci of sulfate and iron oxide reduction shifted into the sediment and conditions favored phosphatization^18^. Oxygen pulses are common near oxygen minimum zones^16^, and records of benthic organisms attest to short-lived intervals of oxia in anoxic basins^60^. Fluctuating seawater redox conditions during the TOAE spanned different depositional environments and ocean basins globally and opened the taphonomic window essential for exceptional preservation.

In summation, this comparative study enhances our understanding of the causes of soft-tissue phosphatization during the Early Jurassic OAE, a critical time in Earth history. Lagerstätten preserved during this episode allow for direct comparison of the taphonomy of the Panthalassa (Ya Ha Tinda) and Tethys Ocean basins (Strawberry Bank and Posidonia Shale). This work suggests that pulses of ocean anoxia lead to widespread preservation of Lagerstätten irrespective of depositional environment. Thus, our results indicate that lagerstätten may occur in a multitude of OAE facies. Use of this search criterion may lead to discoveries of exceptionally preserved fossils in other OAE (e.g. Cretaceous) intervals and provide new insights into the history of marine life.

## Supplementary Information

Supplementary Information.
