# Supplementary material for: Global controls on phosphatization of fossils during the Toarcian Oceanic Anoxic Event
Source: Sci Rep. 2021 Dec 16;11:24087. doi: 10.1038/s41598-021-03482-7 (PMC8677819; doi:10.1038/s41598-021-03482-7)
Supplement: Supplementary file 1 — Supplementary Information. [file 41598_2021_3482_MOESM1_ESM.pdf]

Supplementary material to "Global controls on phosphatization of fossils during the Toarcian Oceanic Anoxic Event"

Sinjini Sinha[1*], A. D. Muscente[1,2], James D. Schiffbauer[3,4], Matt Williams[5], Günter Schweigert[6], and Rowan C. Martindale[1]

[1] *Department of Geological Sciences, The University of Texas at Austin, 2275 Speedway, Austin, Texas, 78712, USA*

[2] *Department of Geology, Cornell College, 600 First Street SW, Mount Vernon, Iowa, 52314, USA*

[3] *Department of Geological Sciences, University of Missouri, 101 Geological Sciences Building, Columbia, Missouri, 65211, USA*

[4] *X-ray Microanalysis Core Facility, University of Missouri, 1 Geological Sciences Building, Columbia, Missouri, 65211, USA*

[5] *Bath Royal Literary and Scientific Institution, 16–18 Queen Square, Bath BA1 2HN, UK*

[6] *Staatliches Museum für Naturkunde, Rosenstein 1, 70191, Stuttgart, Germany*

MATERIALS AND METHODS

Materials

Multiple specimens of fishes, crustaceans, and coleoids from the Strawberry Bank, Ya Ha Tinda, and Posidonia Shale lagerstätten were analyzed (see supplemental data table S1). The Strawberry Bank specimens are reposited in the Bath Royal Literary and Scientific Institution (BRLSI) in Bath, United Kingdom, and were collected between 1815 and 1881, primarily by Charles Moore. All specimens from the Posidonia Shale used in this study are reposited in the Non-Vertebrate Paleontology Lab (NPL) at the University of Texas at Austin (UT Austin) in Texas, United States. These specimens were obtained from surface collection at the Kromer Quarry in Ohmden and the Holcim quarry in Dormettingen near Dotternhausen (often referred as the Dotternhausen quarry). Additional specimens from the Posidonia Shale were donated by Günter Schweigert and Erin Maxwell of the State Museum of Natural History in Stuttgart, Germany. The Ya Ha Tinda specimens are reposited at the Royal Tyrrell Museum of Palaeontology (RTMP) in Drumheller, Alberta, Canada, and were collected during fieldwork between 2013 and 2019. Ya Ha Tinda fossils were collected under Parks Canada permit #YHTR-2014-16156 and Alberta Government Palaeontological Permits RTMP 13-058, 14-009, 15-019, 16-063, 17-048, and 18-072.

Photography and microscopy

All fossils were photographed by either reflected light microscope or digital single lens reflex camera. Since they are so small, the Strawberry Bank fossils were photographed using a Zeiss Discovery V8 microscope and Zen software; in contrast, the Posidonia Shale and Ya Ha Tinda fossils were photographed under normal (non-polarized), high-angle, bidirectional light

with a Nikon d3200 digital single lens reflex camera variably equipped with zoom, wide-angle, and macros lenses. The brightness and contrast of photographs were digitally adjusted using Adobe Photoshop. A combination of microscopic and photographic methods were employed to document the presence of specific tissues in the fossils, including the eyes, guts, and caudal fins of the fishes; the exoskeletal layers of the crustaceans; and the ink sacs, gladii, and mantle tissues of the coleoids.

Scanning electron microscopy and energy-dispersive X-ray spectroscopy

Scanning electron microscopy (SEM) and energy dispersive X-Ray spectroscopy (EDS) were used to characterize the microstructures and elemental compositions of the fossils[1-3], including the authigenic and diagenetic minerals that they contain due to preservational processes[4], like phosphatization, pyritization, calcification, and aluminosilicification[5]. The electron microscopy work included secondary electron SEM (SE-SEM), which produces with high topographic contrast, and backscattered electron SEM (BSE-SEM), which produces images known for compositional contrast based on the average atomic number (Z) of each material in a sample[1,3]. The EDS analyses were conducted to collect semi-quantitative data on the concentrations of elements in the various fossil materials; these data are presented in the form of elemental maps.

Small specimens from Strawberry Bank (3 cm or smaller across its longest axis) were analyzed at the Electron Microbeam Laboratory at the Department of Geological Sciences, University of Texas at Austin (UT Austin), using a JEOL-6490LV SEM equipped with a tungsten filament electron source, BSE and SE detectors, and an EDAX Apollo 11 silicon-drift EDS detector. The small specimens from the Posidonia Shale were studied at the Materials

Analysis, Testing, and Fabrication (MATFab) Facility at the University of Iowa using a Hitachi S-3400N SEM equipped with a tungsten filament electron source, BSE and SE detectors, and XFlash silicon drift detector. All large specimens (i.e., specimens larger than 3 cm along their longest axis) were analyzed at the X-ray Microanalysis Core Laboratory, University of Missouri (Mizzou), using a Zeiss Sigma 500VP SEM equipped with a Schottky field emission electron source, a 5-segment high-definition solid-state BSE detector (HDBSD), a cascade current low vacuum secondary electron detector (C2D), an Atlas 5 correlative microscopy workflow system, and dual Bruker XFlash 6|30 silicon-drift EDS detectors. This SEM system was used to acquire large 'mosaic' images through an automated process of gathering and combining numerous smaller images, thereby allowing for the study of large specimens (and large surface areas), which cannot be visualized using common and conventional SEM instruments.

All samples analyzed were uncoated and unpolished, with the exception of one *Uncina posidoniae* lobster claw (Specimen number NPL00036039.000), which was discovered in a polished slab that was donated to researchers at the State Museum of Natural History Stuttgart. At all facilities, the SEM chamber pressure was held at low vacuum (1-35 Pa) during the analyses, allowing chamber gases to disperse electron charge; this dispersal eliminated the need for the damaging application of conductive coatings[1,3]. Given the use of three SEM instruments, operating conditions inherently varied, though efforts were taken to ensure comparable analytical parameters. At UT Austin and MATFab, BSE images were acquired with an accelerating voltage of 20 keV, spot size of 45-50, and a working distance between 9-24 mm. At Mizzou, all SEM work was conducted with a beam accelerating voltage of 20 keV, beam current of 40 nA, a 60 µm aperture, and a working distance of 16-20 mm. Large image mosaics of both BSE and SE

signals, using the HDBSD and C2D respectively, were assembled from full fossil surfaces using the Atlas 5 workflow.

Similarly, SEM-EDS was conducted to obtain elemental concentration variation at UT Austin, MATFab, and Mizzou with differences in operating conditions. At UT Austin, spectral and areal elemental mapping data collected using the EDAX Genesis Apollo 11 system were processed using the EDAX Genesis spectrum software (Version 6.43), with operating conditions as follows: beam accelerating voltage 20 keV, working distance 10-12 nm, and spot size 99. These settings yielded X-ray count rates of over 100,000 counts per second. Likewise, at MATFab, the elemental data collected by the XFlash silicon drift detector were analyzed using Quantax software and the following operating parameters: beam accelerating voltage 20 keV, working distance 10-12 nm, and spot size 99. Under these conditions, the X-ray count rates were between 10,000 and 30,000, which are sufficient for elemental analysis. At Mizzou, the operating conditions used for SEM imaging were maintained for SEM-EDS analyses, with the exception of the aperture, which was increased to 120 µm to ensure higher X-ray count rates. Spectral and areal elemental mapping data were collected using the dual, coplanar Bruker XFlash 6|30 EDS detector units in tandem, yielding X-ray count rates on the order of 300,000 counts per second. Elements that were mapped included aluminum (Al), barium (Ba), carbon (C), calcium (Ca), fluorine (F), iron (Fe), magnesium (Mg), phosphorus (P), potassium (K), silicon (Si), sodium (Na), sulfur (S), and zinc (Zn).

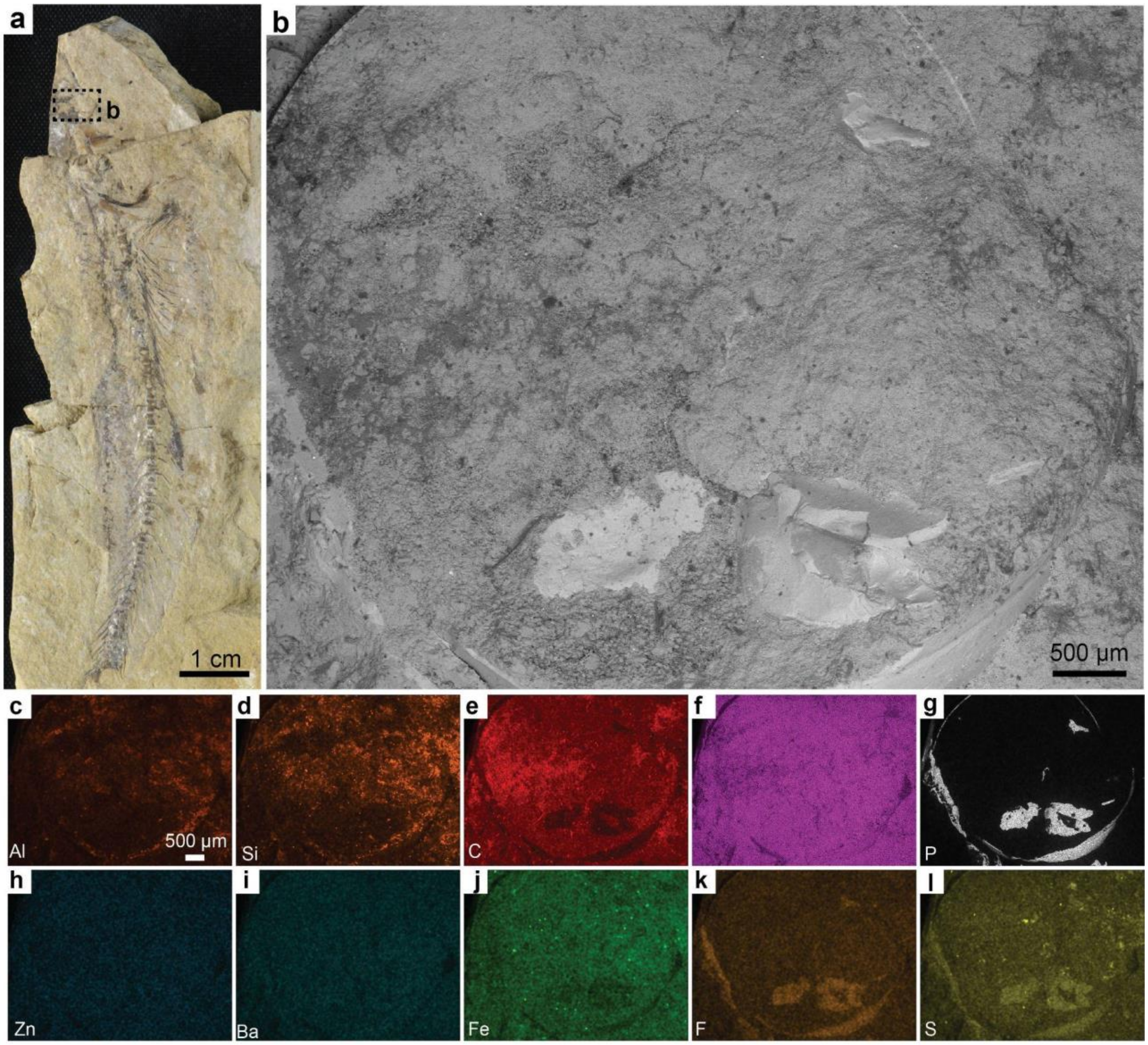

Supplementary Figure S1. Fish fossil *Leptolepis* (BRLSI.M1261) from the Strawberry Bank Lagerstätte (UK) in a limestone concretion. **a**, Reflected-light photograph of the specimen. **b**, Magnified BSE-SEM image of box in **a**, showing the eye of the fish. **c-l**, EDS elemental maps of **b**, showing phosphatized bone near the eye and encrusted by calcite.

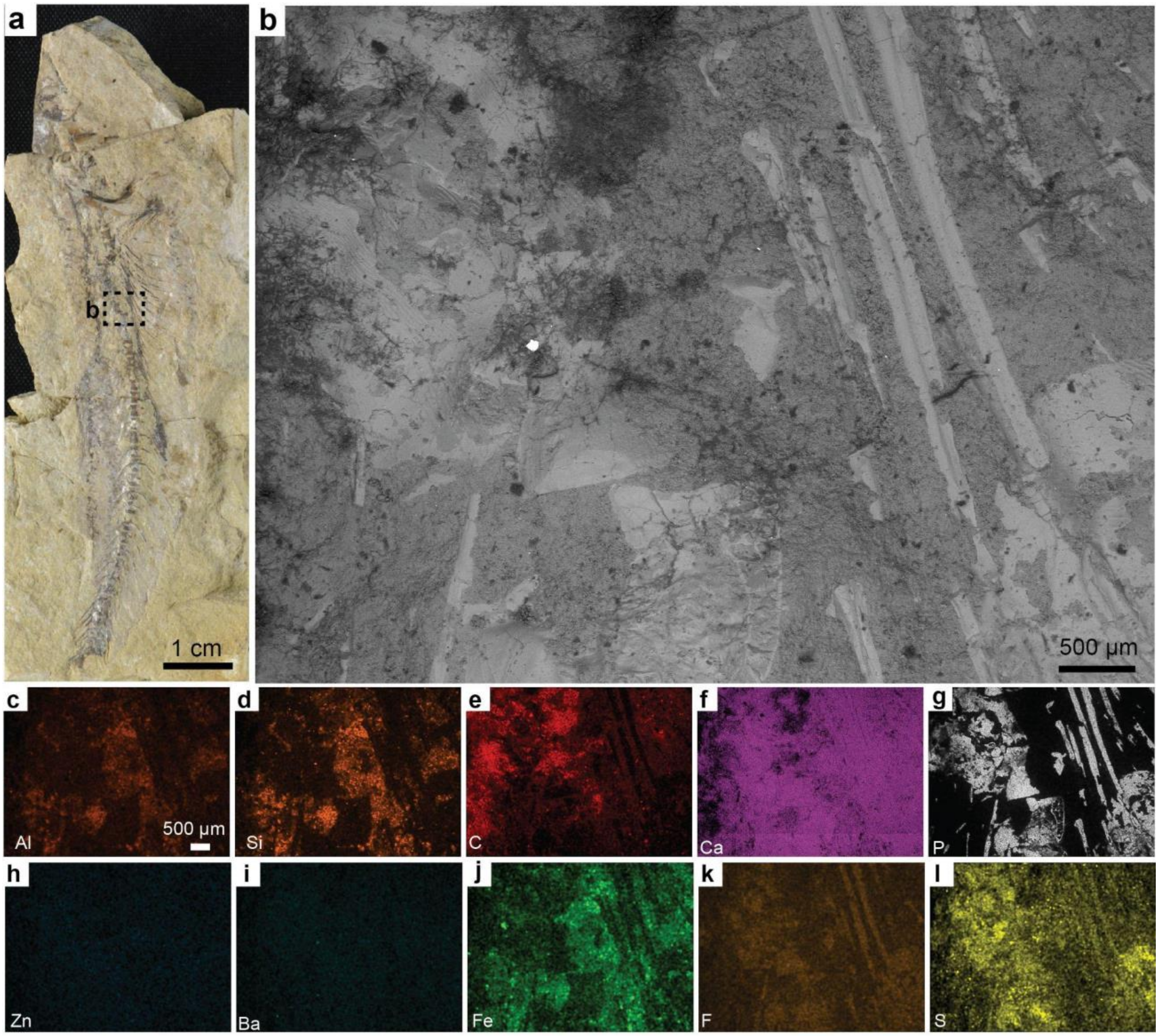

Supplementary Figure S2. Fish fossil *Leptolepis* (BRLSI.M1261) from the Strawberry Bank Lagerstätte (UK) in a limestone concretion. **a**, Reflected-light photograph of specimen. **b**, Magnified BSE-SEM image of box in **a**, showing the vertebral column and fin rays of the fish. **c-l**, EDS elemental maps of **b**, showing phosphatized bone near the gut, traces of iron, fluorine, and carbon.

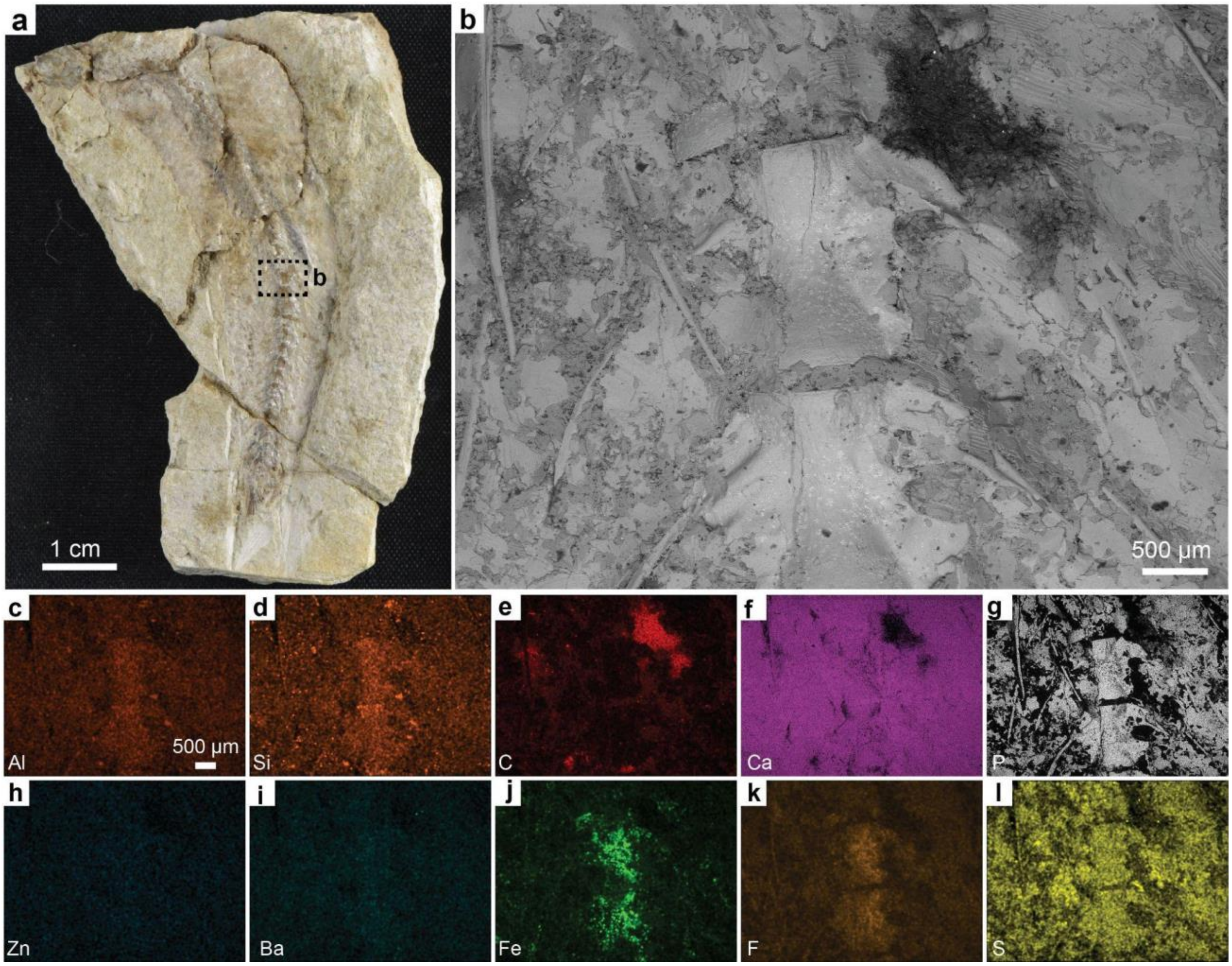

Supplementary Figure S3. Fish fossil *Leptolepis* (BRLSI.M1261A) from the Strawberry Bank Lagerstätte (UK) in a limestone concretion. **a**, Reflected-light photograph of the specimen. **b**, Magnified BSE-SEM image of box in **a**, showing the vertebral column of the fish. **c-l**, EDS elemental maps of **b**, showing phosphatized vertebral centra.

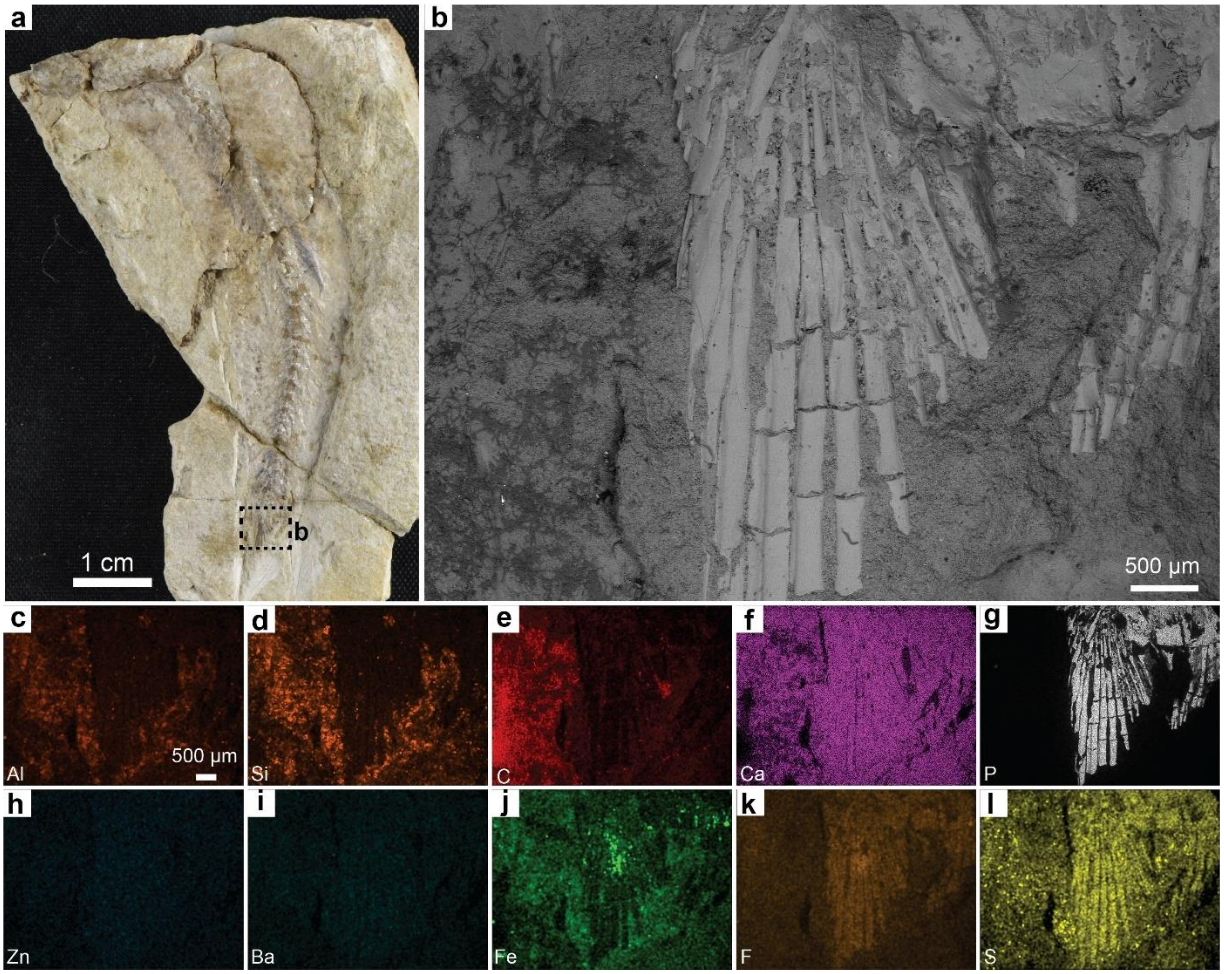

Supplementary Figure S4. Fish fossil *Leptolepis* (BRLSI.M1261A) from the Strawberry Bank Lagerstätte (UK) in a limestone concretion. **a**, Reflected-light photograph of the specimen. **b**, Magnified BSE-SEM image of box in **a**, showing the caudal fin (tail) of the fish. **c-l**, EDS elemental maps of **b**, showing phosphatized tail bones encrusted by calcite, and the alumino-silicate rich surrounding matrix.

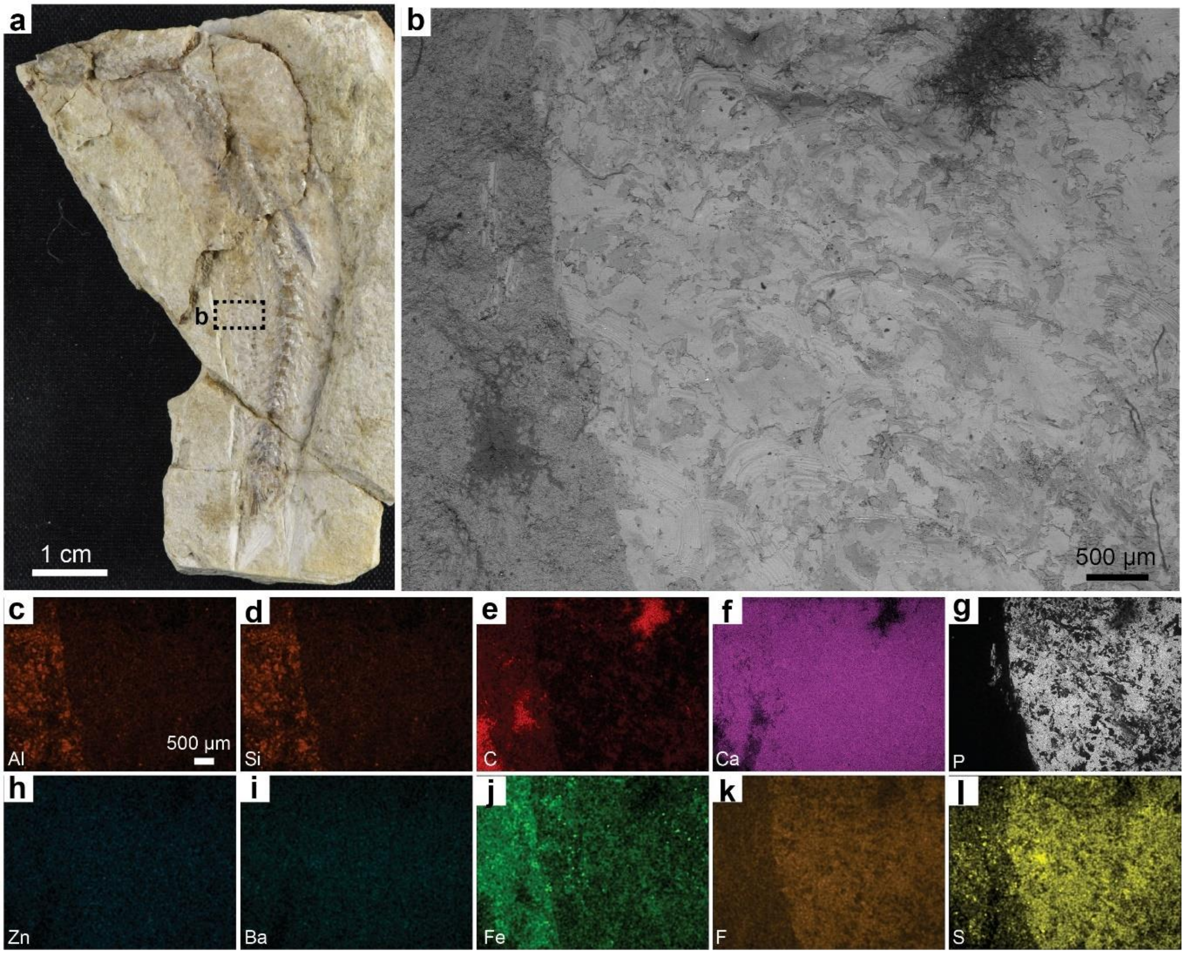

Supplementary Figure S5. Fish fossil *Leptolepis* (BRLSI.M1261A) from the Strawberry Bank Lagerstätte (UK) in a limestone concretion. **a**, Reflected-light photograph of the specimen. **b**, Magnified BSE-SEM image of box in **a**, showing the phosphatized gut. **c**-**l**, EDS elemental maps of **b**.

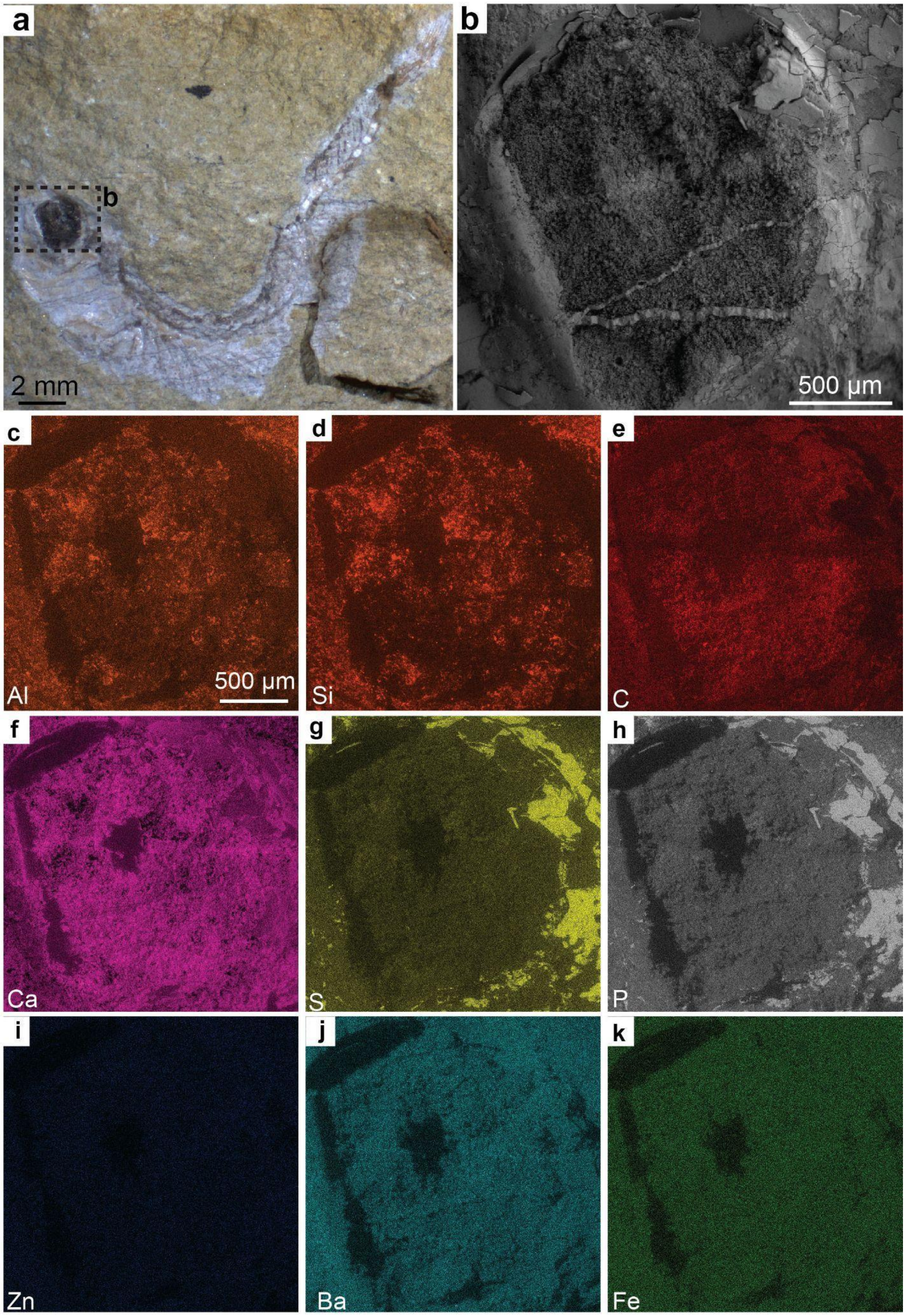

Supplementary Figure S6. Fish fossil *Leptolepis* (BRLSI.M1269A) from the Strawberry Bank Lagerstätte (UK) in a limestone concretion. **a**, Reflected-light photograph of the specimen. **b**, Magnified BSE-SEM image of box in **a**, showing the eye. **c-k**, EDS elemental maps of **b**, showing phosphatized bone near the eye.

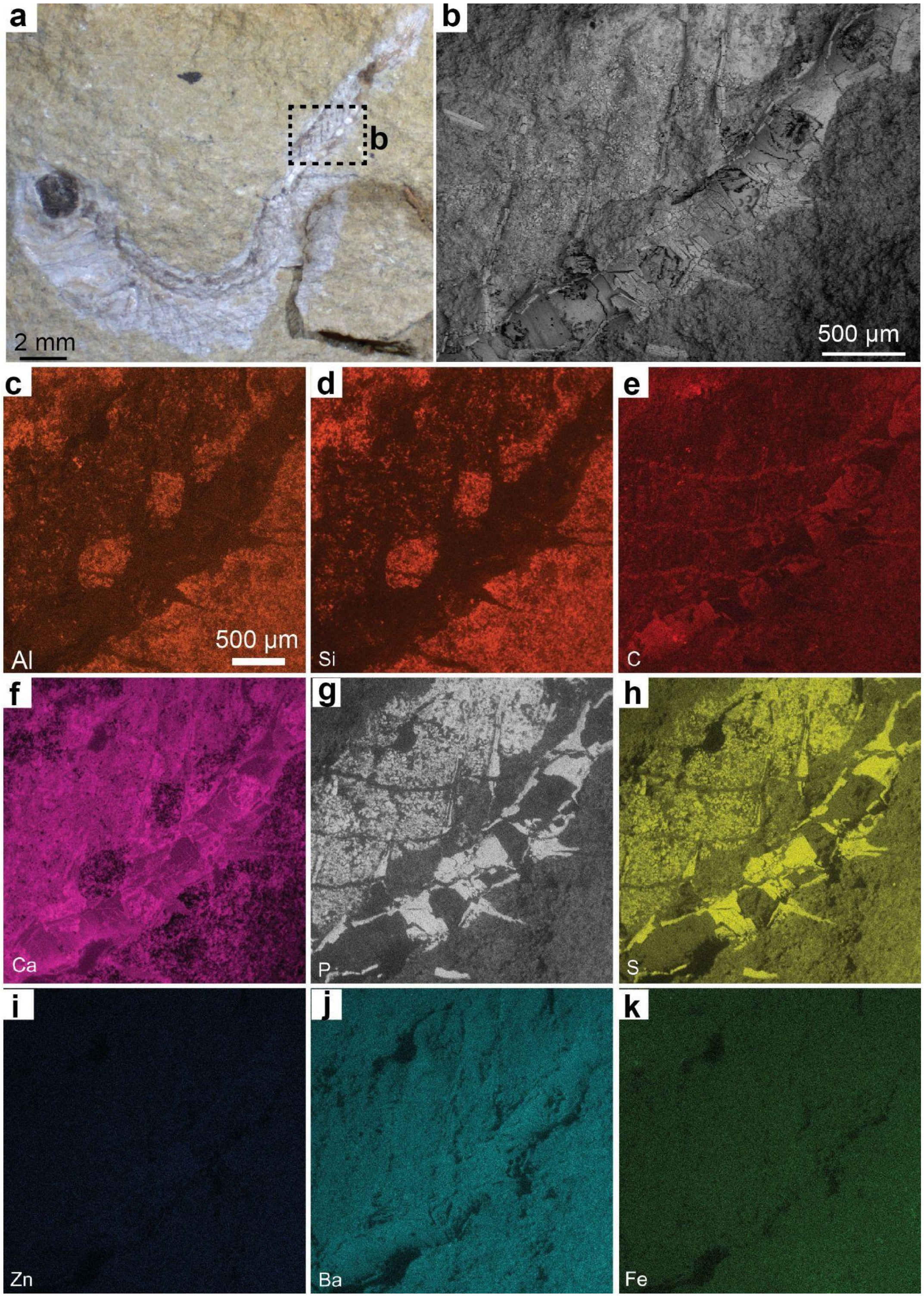

Supplementary Figure S7. Fish fossil *Leptolepis* (BRLSI.M1269A) from the Strawberry Bank Lagerstätte (UK) in a limestone concretion. **a**, Reflected-light photograph of the specimen. **b**, Magnified BSE-SEM image of the box in **a**, showing the vertebral column and the gut area of the fish. **c-k**, EDS elemental maps of **b**, showing phosphatized gut.

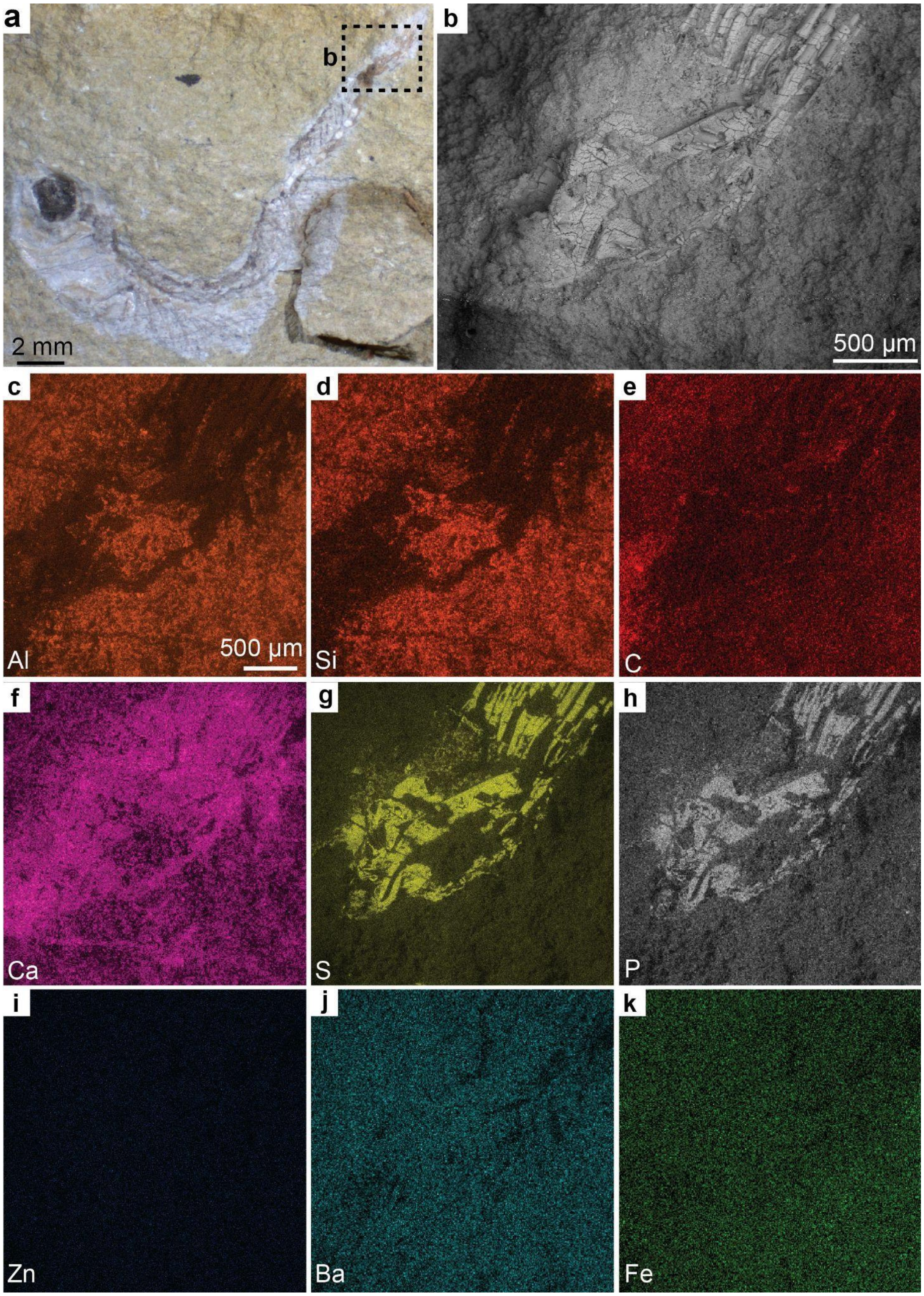

Supplementary Figure S8. Fish fossil *Leptolepis* (BRLSI.M1269A) from the Strawberry Bank Lagerstätte (UK) in a limestone concretion. **a**, Reflected-light photograph of the specimen. **b**, Magnified BSE-SEM image of the box in **a**, showing the caudal fin (tail) of the fish. **c-k**, EDS elemental maps of **b**, showing phosphatized and sulfur-rich tail bones and the alumino-silicate rich surrounding matrix.

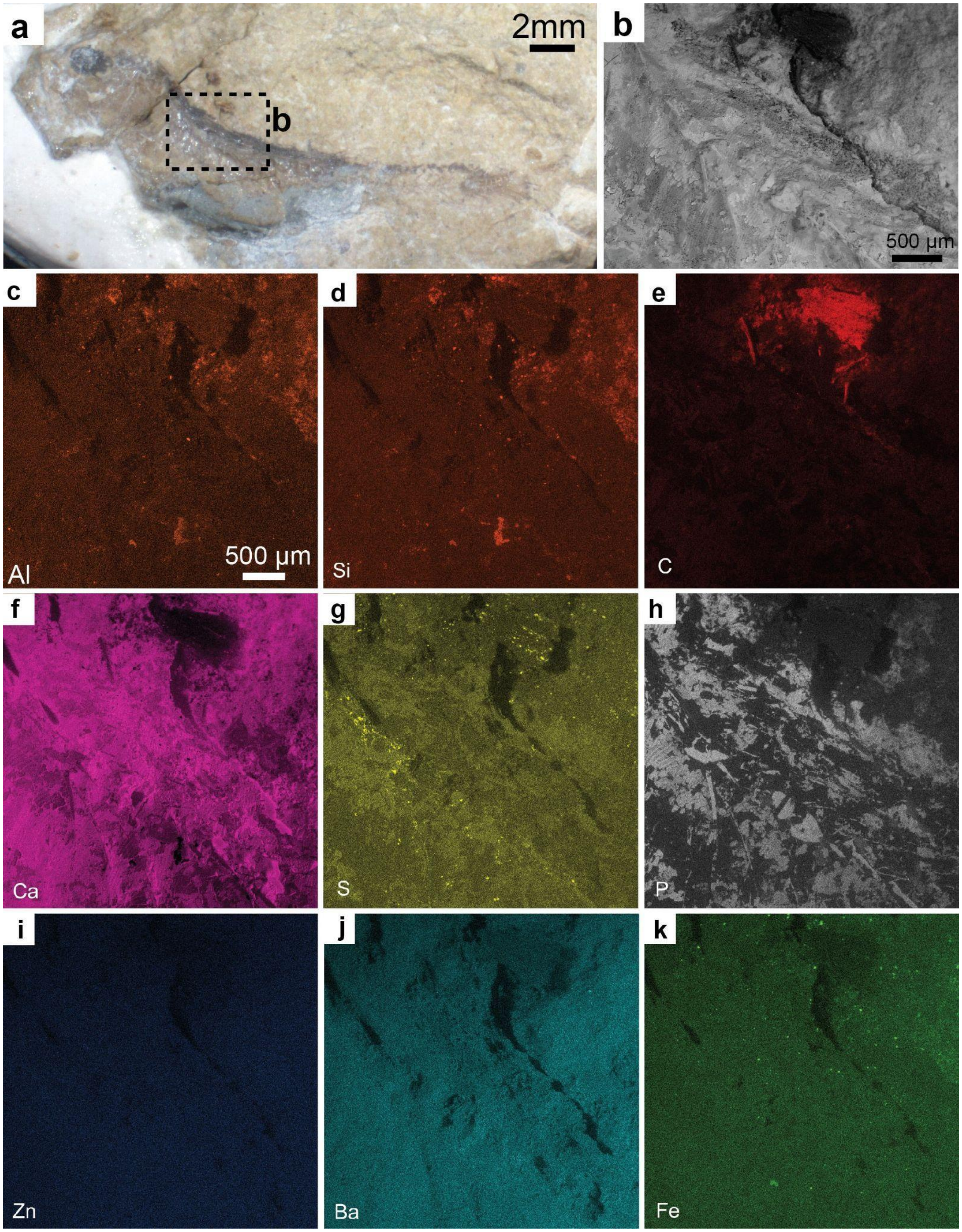

Supplementary Figure S9. Fish fossil *Leptolepis* (BRLSI.M1271A) from the Strawberry Bank Lagerstätte (UK) in a limestone concretion. **a**, Reflected-light photograph of the specimen. **b**, Magnified BSE-SEM image of the box in **a**, showing the vertebral column of the fish. **c-k**, EDS elemental maps of **b**, showing phosphatized bones.

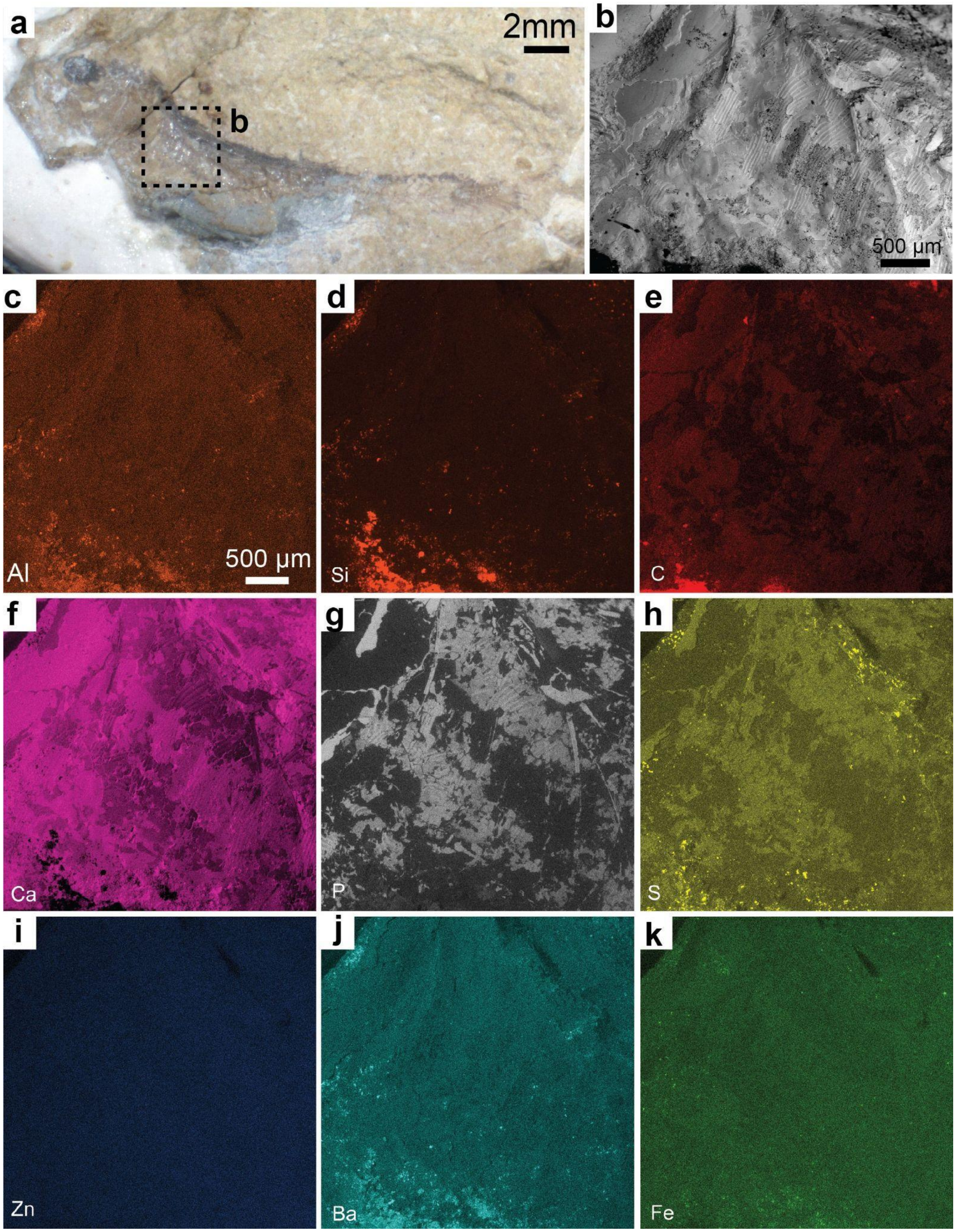

Supplementary Figure S10. Fish fossil *Leptolepis* (BRLSI.M1271A) from the Strawberry Bank Lagerstätte (UK) in a limestone concretion. **a**, Reflected-light photograph of the specimen. **b**, Magnified BSE-SEM image of box in **a**. **c-k**, EDS elemental maps of **b**, showing phosphatized bones.

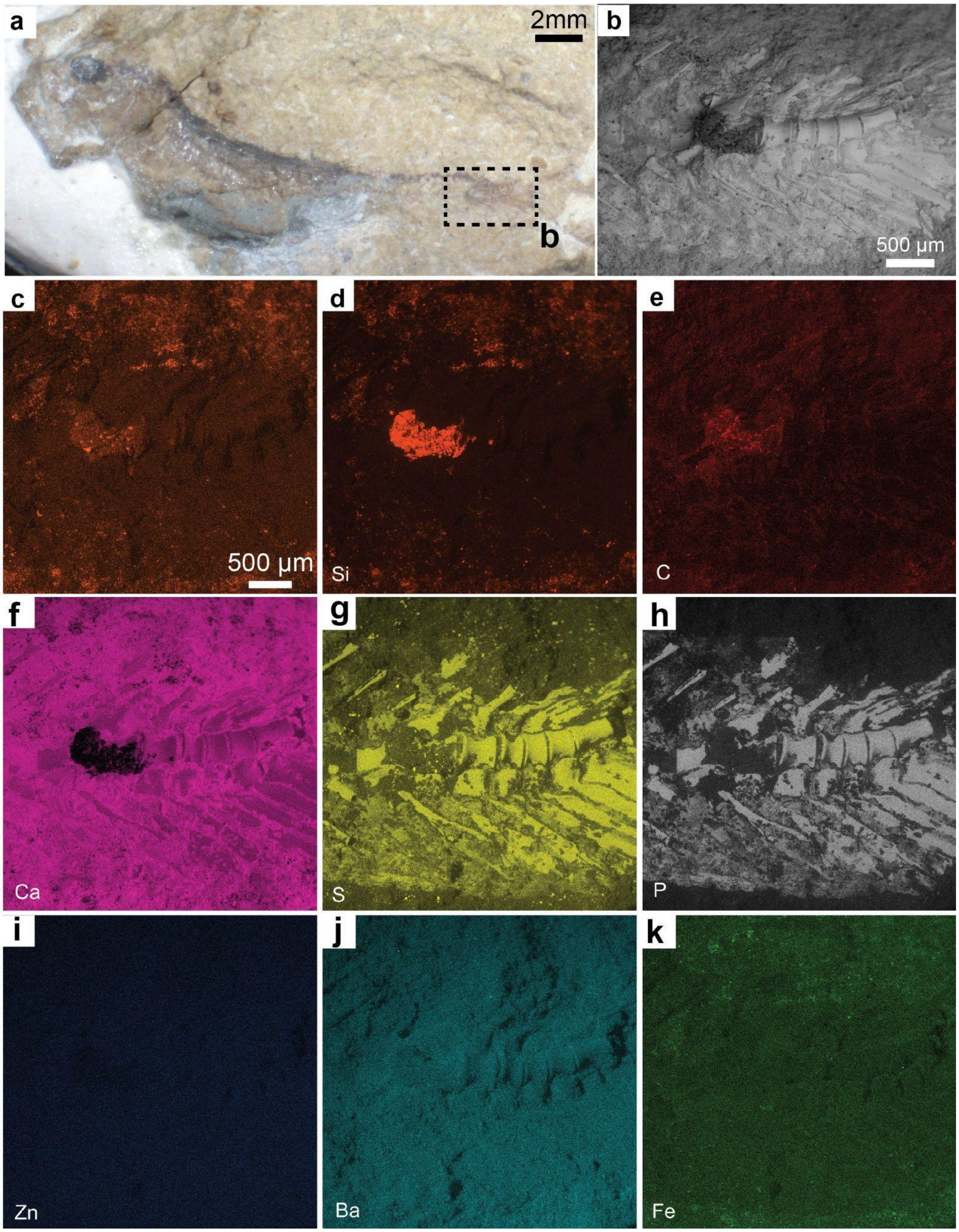

Supplementary Figure S11. Fish fossil *Leptolepis* (BRLSI.M1271A) from the Strawberry Bank Lagerstätte (UK) in a limestone concretion. **a**, Reflected-light photograph of the specimen. **b**, Magnified BSE-SEM image of the box in **a**, showing the posterior part of the vertebral column of the fish. **c-k**, EDS elemental maps of **b**, showing phosphatized, calcium and sulfur-rich vertebral column.

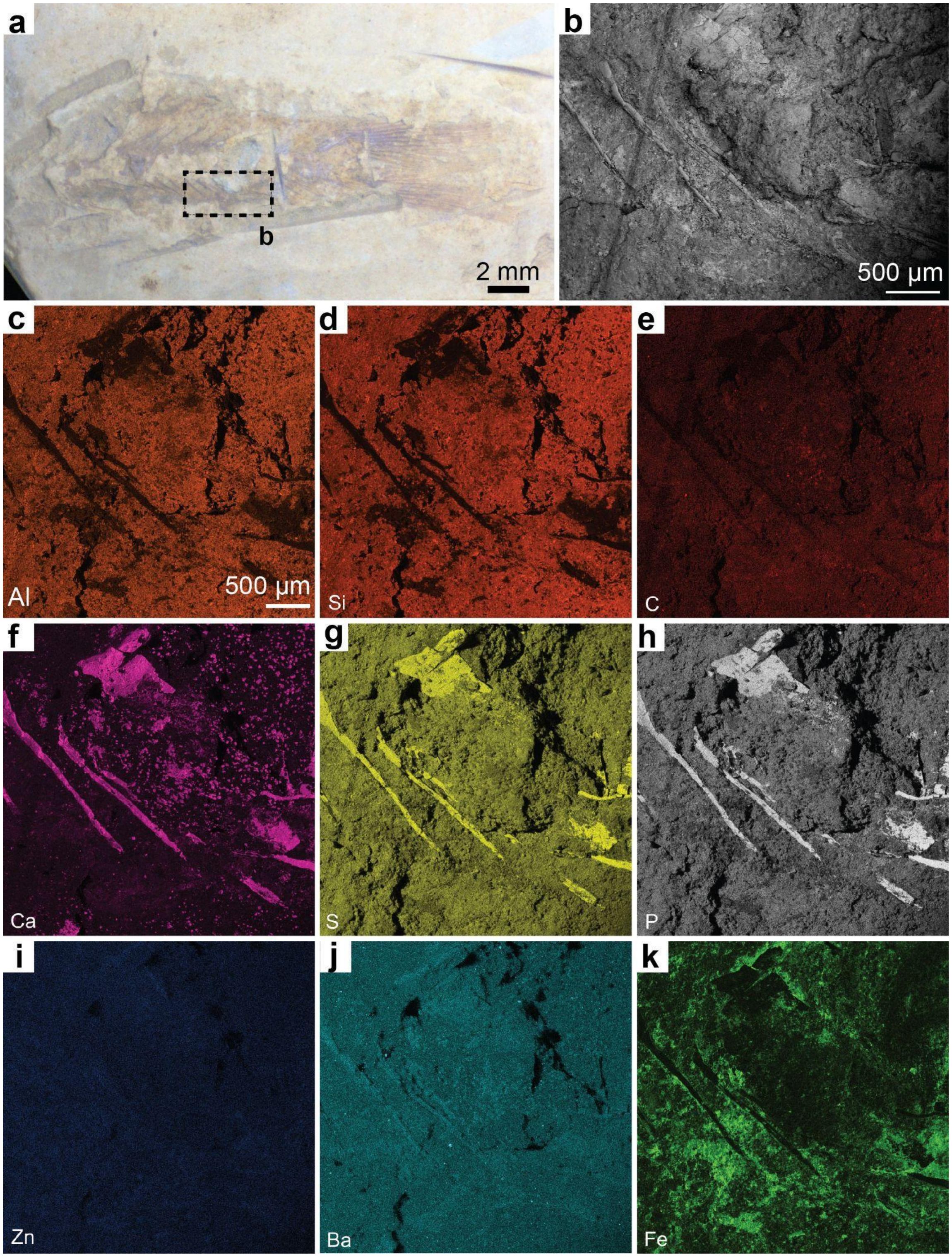

Supplementary Figure S12. Fish fossil (BRLSI.M1275) from the Strawberry Bank Lagerstätte (UK) in a clay-rich limestone matrix. **a**, Reflected-light photograph of the specimen. **b**, Magnified BSE-SEM image of the box in **a**, showing the gut area of the fish. **c-k**, EDS elemental maps of **b**, showing phosphatized, calcium and sulfur-rich bones.

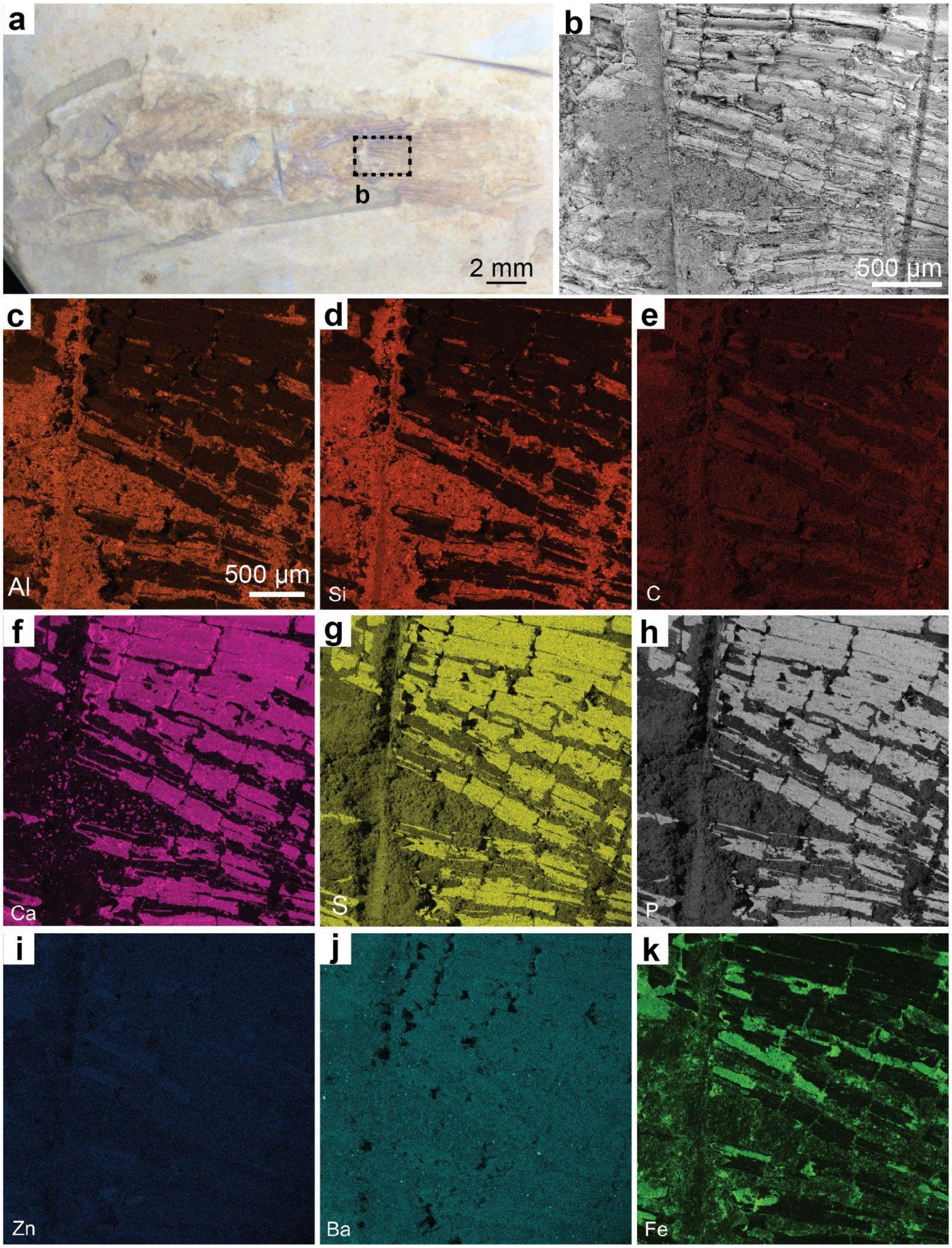

Supplementary Figure S13. Fish fossil (BRLSI.M1275) from the Strawberry Bank Lagerstätte (UK) in a clay-rich limestone matrix. **a**, Reflected-light photograph of the specimen. **b**, Magnified BSE-SEM image of the box in **a**, showing the caudal fin (tail) of the fish. **c**-**k**, EDS elemental maps of **b**, showing phosphatized, calcium and sulfur-rich fish tail and the alumino-silicate rich surrounding matrix.

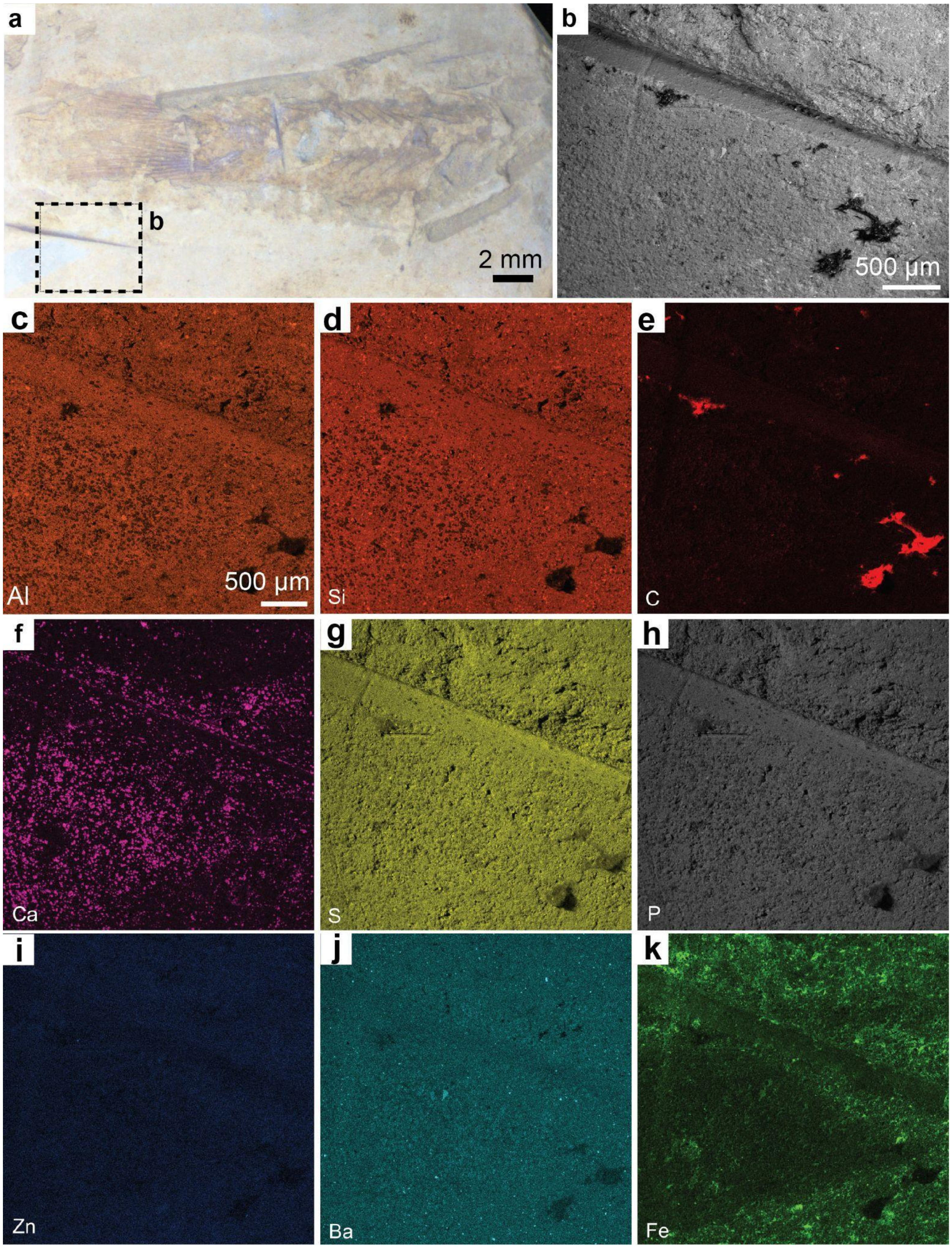

Supplementary Figure S14. Fish fossil (BRLSI.M1275) from the Strawberry Bank Lagerstätte (UK) in a clay-rich limestone matrix. **a**, Reflected-light photograph of the specimen. **b**, Magnified BSE-SEM image of box in **a**, focusing on the matrix containing the fish. **c-k**, EDS elemental maps of **b**.

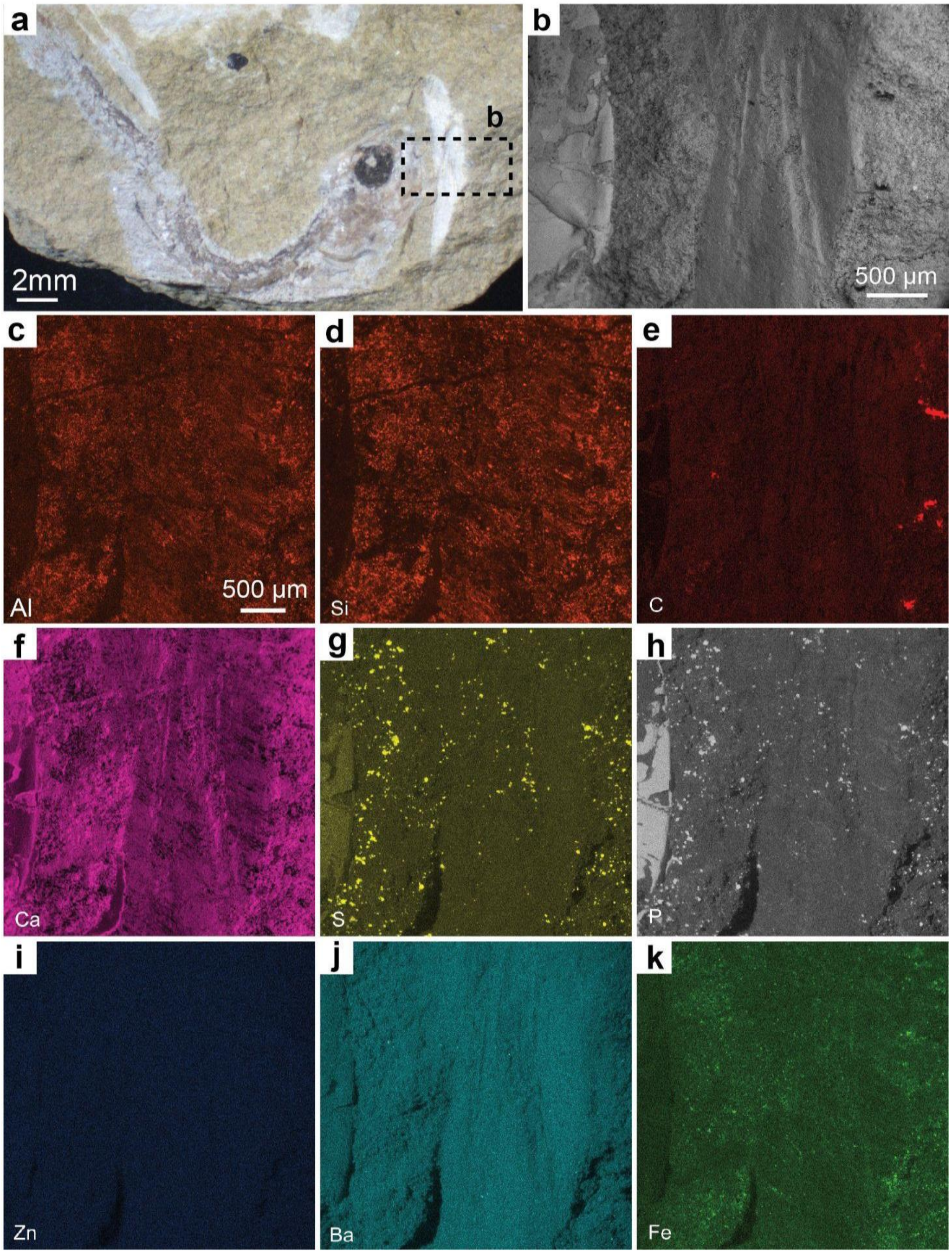

Supplementary Figure S15. Fish fossil *Leptolepis* (BRLSI.M1269) from the Strawberry Bank Lagerstätte (UK) in a limestone concretion. **a**, Reflected-light photograph of the specimen. **b**, Magnified BSE-SEM image of the box in **a**, focusing on the matrix near the fish eye. **c-k**, EDS elemental maps of **b**, showing phosphatized bones near the eye and an alumino-silicate rich matrix.

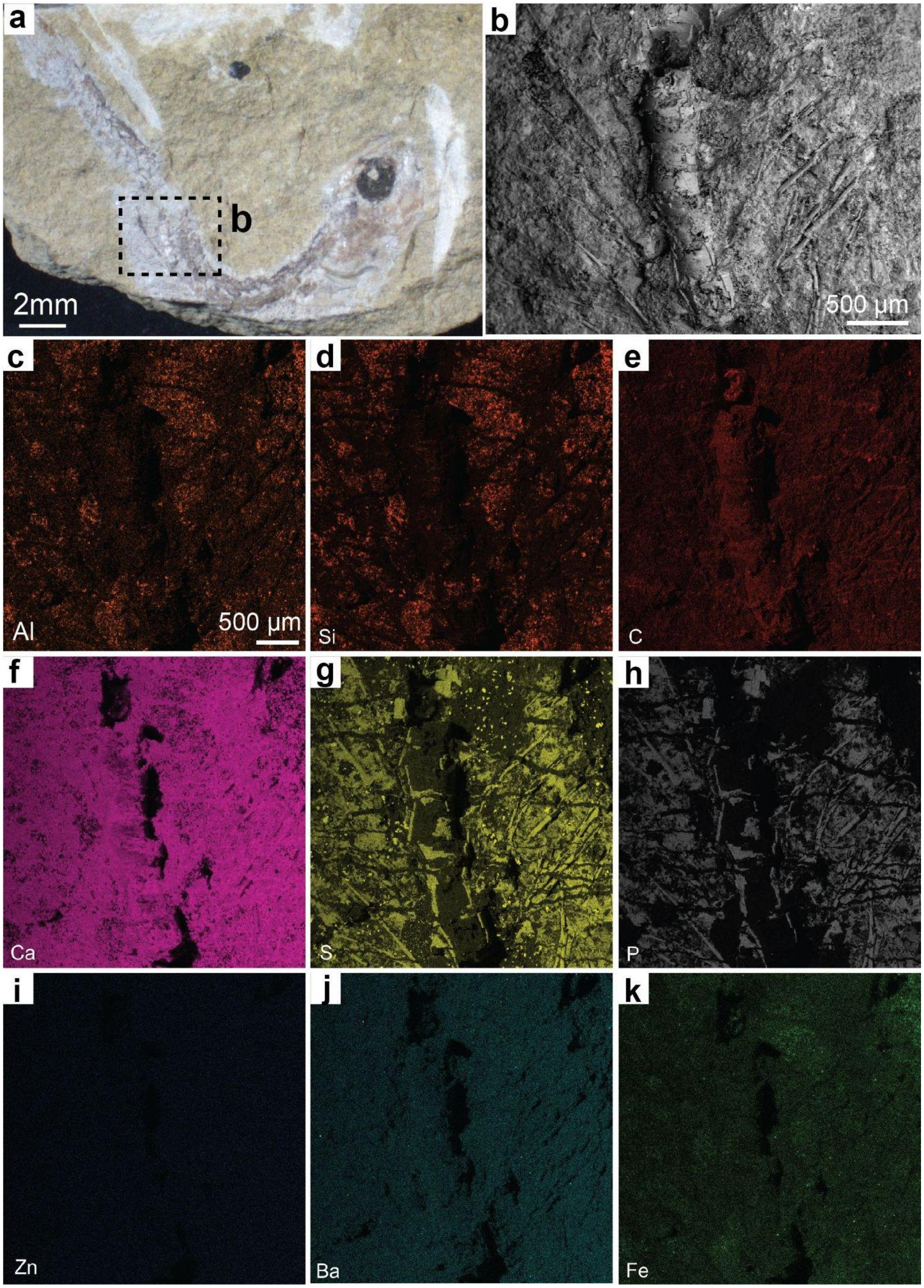

Supplementary Figure S16. Fish fossil *Leptolepis* (BRLSI.M1269) from the Strawberry Bank Lagerstätte (UK) in a limestone concretion. **a**, Reflected-light photograph of the specimen. **b** Magnified BSE-SEM image of box in **a**, showing the vertebral column and the phosphatized fish gut. **c**-**k**, EDS elemental maps of **b**, showing phosphatized, calcium and sulfur-rich gut.

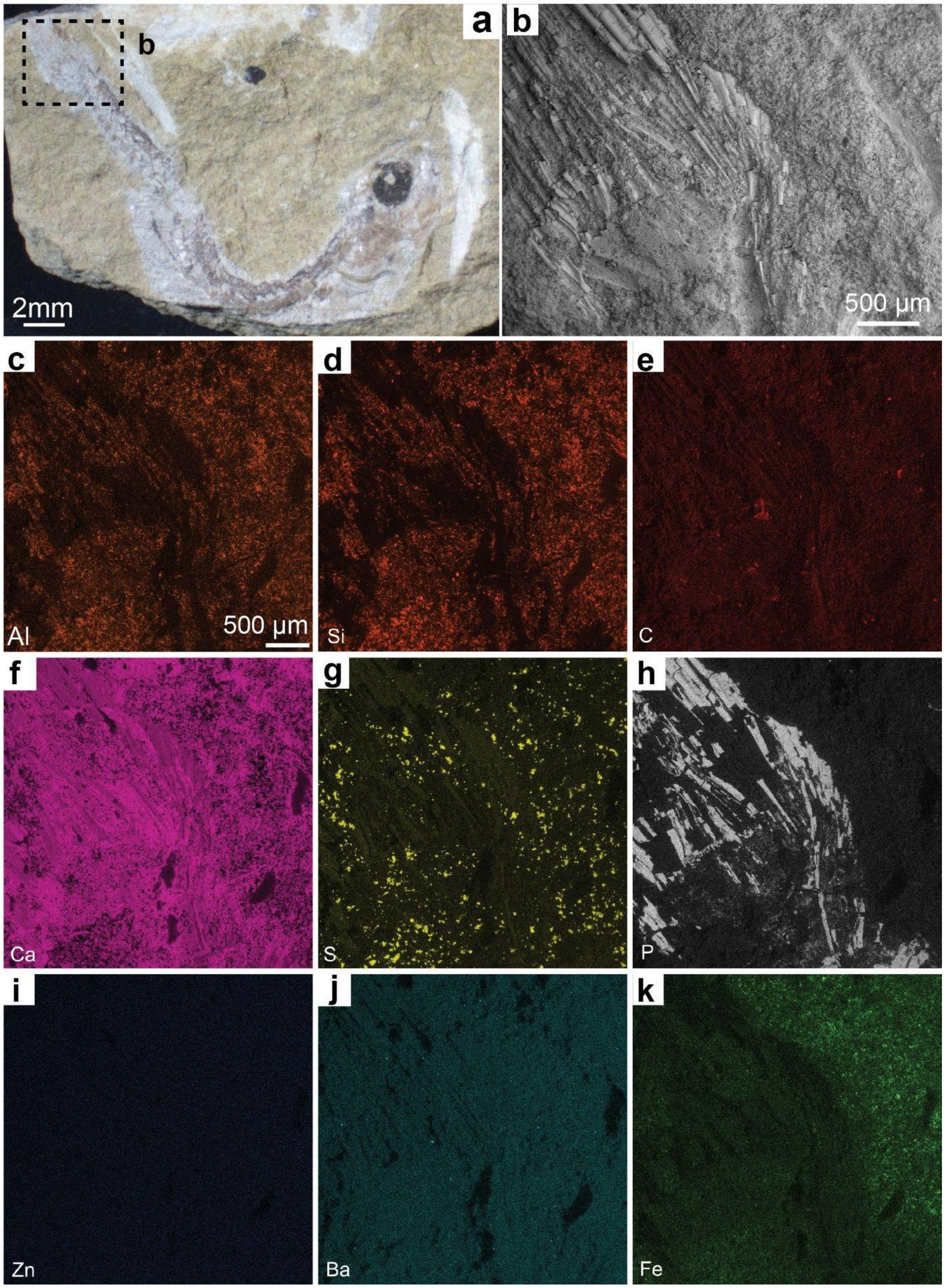

Supplementary Figure S17. Fish fossil *Leptolepis* (BRLSI.M1269) from the Strawberry Bank Lagerstätte (UK) in a limestone concretion. **a**, Reflected-light photograph of the specimen. **b**, Magnified BSE-SEM image of box in **a**, showing the phosphatized fish tail. **c-k**, EDS elemental maps of **b**.

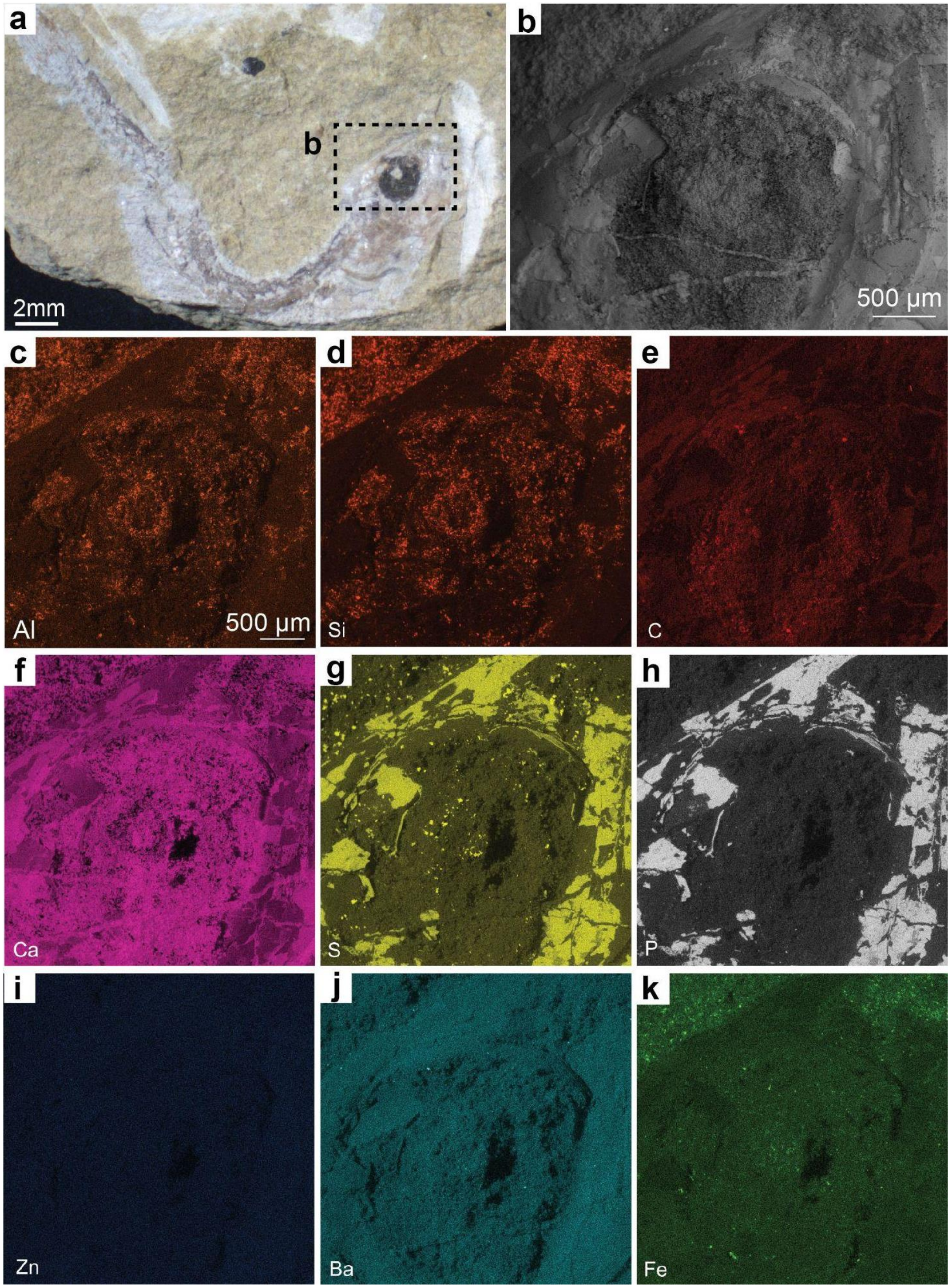

Supplementary Figure S18. Fish fossil *Leptolepis* (BRLSI.M1269) from the Strawberry Bank Lagerstätte (UK) in a limestone concretion. **a**, Reflected-light photograph of the specimen. **b**, Magnified BSE-SEM image of box in **a**, showing the fish eye. **c**-**k**, EDS elemental maps of **b**, showing phosphatized bones near the eye.

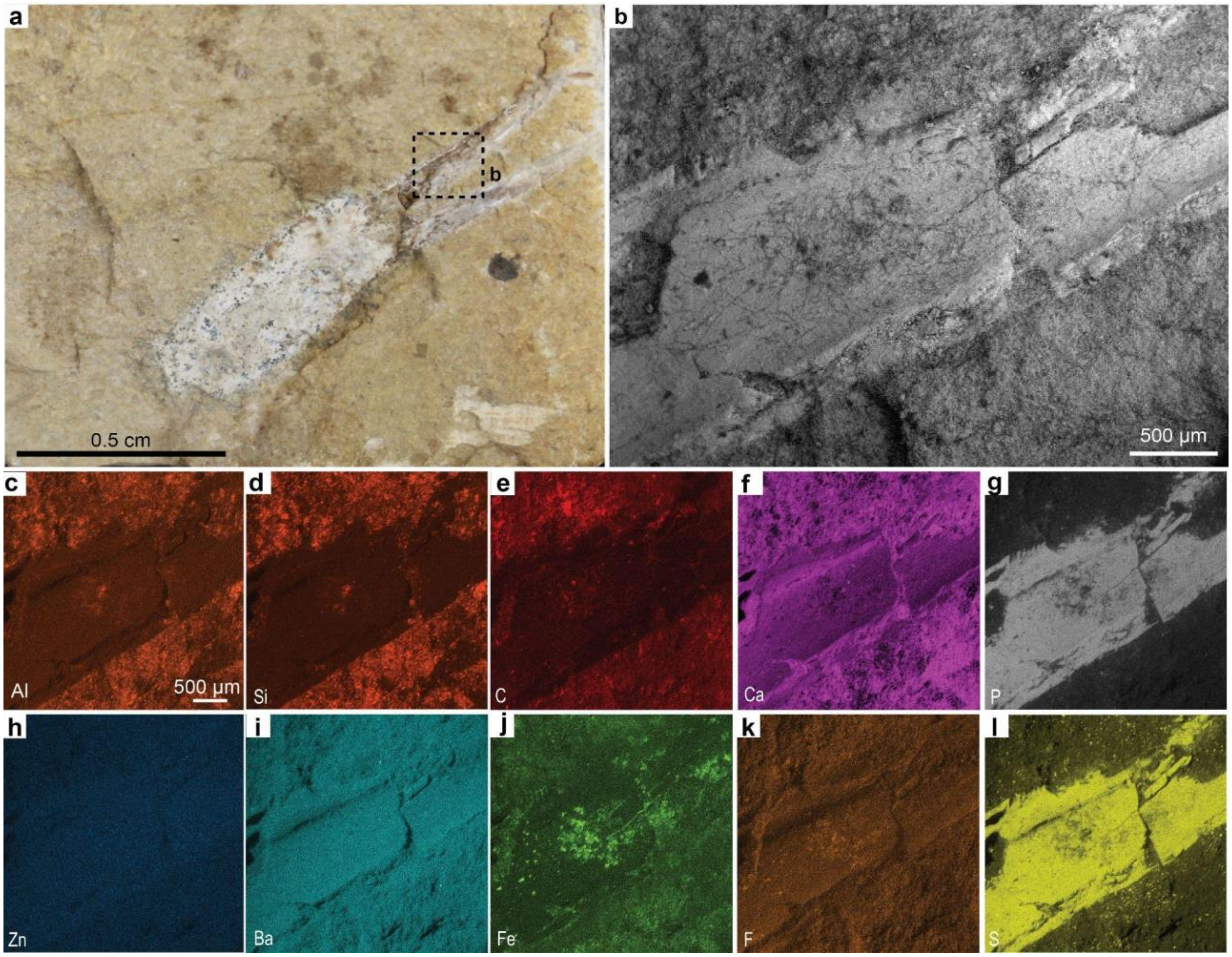

Supplementary Figure S19. Exoskeleton of the lobster *Proeryon* sp. (BRLSI.M1242) from the Strawberry Bank Lagerstätte (UK) in a limestone concretion. **a**, Reflected-light photograph of the specimen. **b**, Magnified BSE-SEM image of box in **a**, showing phosphatized carapace. **c-l**, EDS elemental maps of **b** showing that the exoskeleton is rich in phosphorus and sulfur, in contrast to the surrounding matrix, which is rich in aluminum, silicon, and traces of carbon as well as iron.

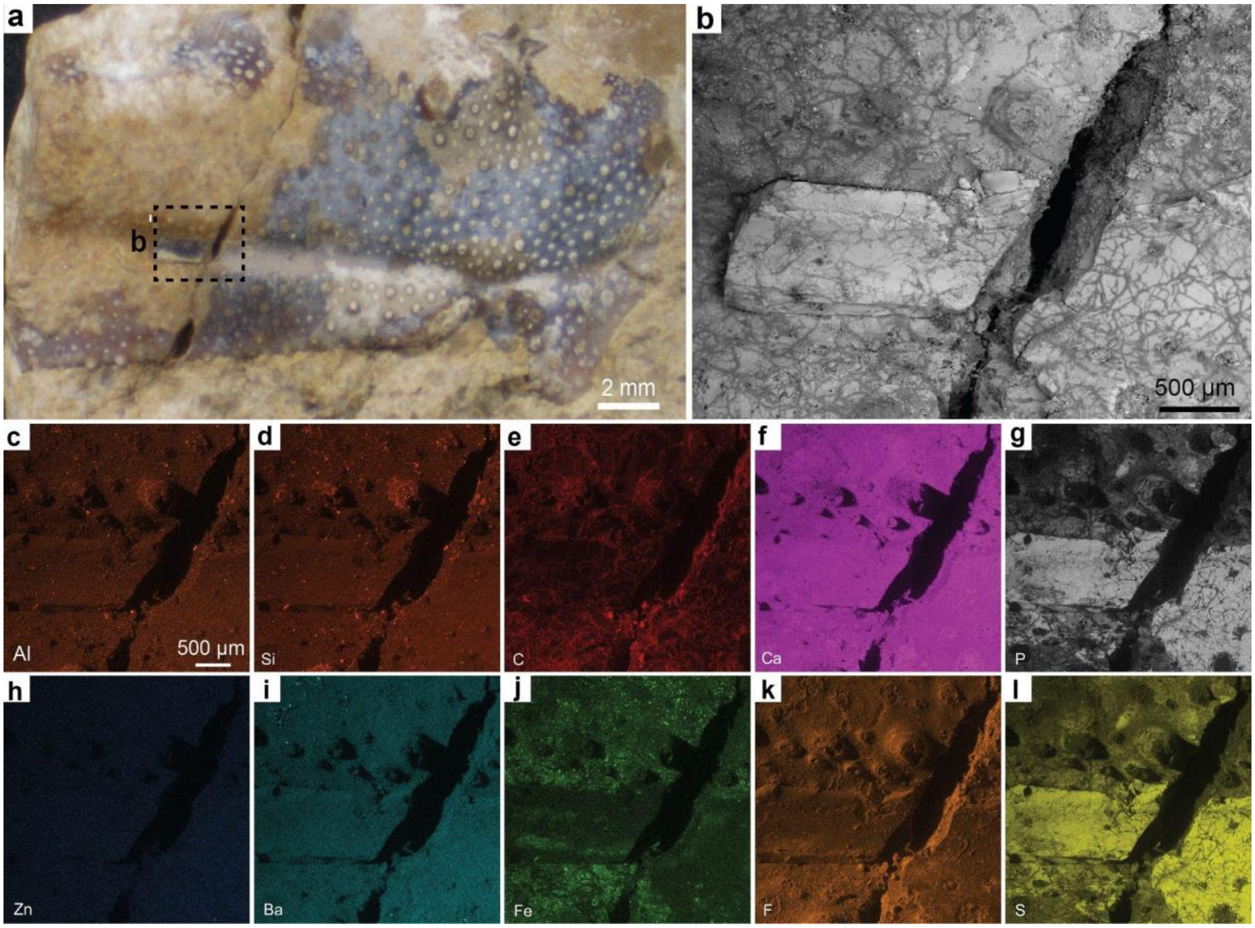

Supplementary Figure S20. Exoskeleton of crustacean (BRLSI.M1243A) from the Strawberry Bank Lagerstätte (UK) in a limestone concretion. **a**, Reflected-light photograph of the specimen. **b**, Magnified BSE-SEM image of the box in **a**, showing phosphatized carapace. **c-l**, EDS elemental maps of **b**, showing the phosphorus and sulfur-rich exoskeleton.

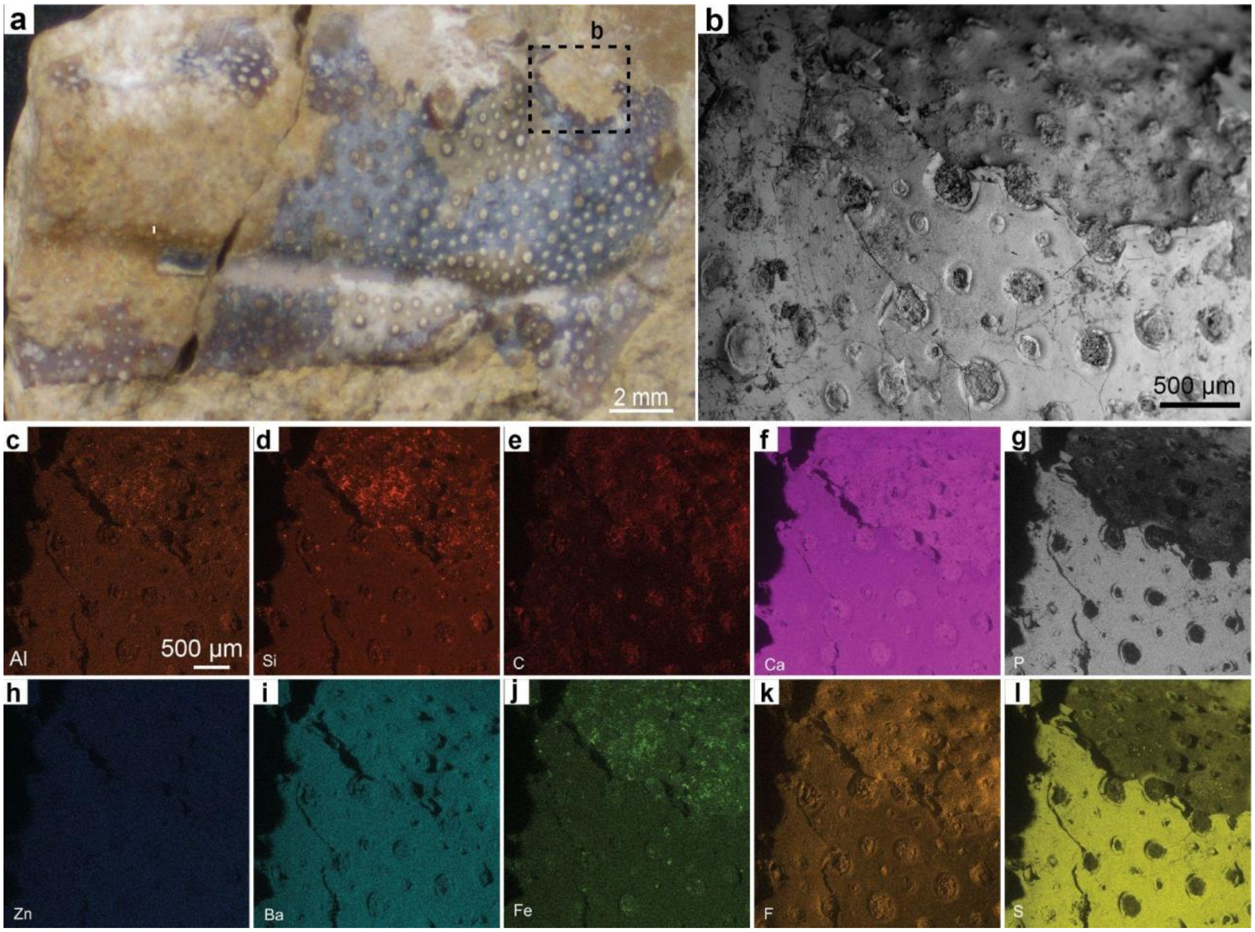

Supplementary Figure S21. Exoskeleton of a crustacean (BRLSI.M1243A) from the Strawberry Bank Lagerstätte (UK) in a limestone concretion. **a**, Reflected-light photograph of specimen. **b**, Magnified BSE-SEM image of box in **a**, showing phosphatized carapace. **c-l**, EDS elemental maps of **b**, showing the phosphorus and sulfur-rich exoskeleton.

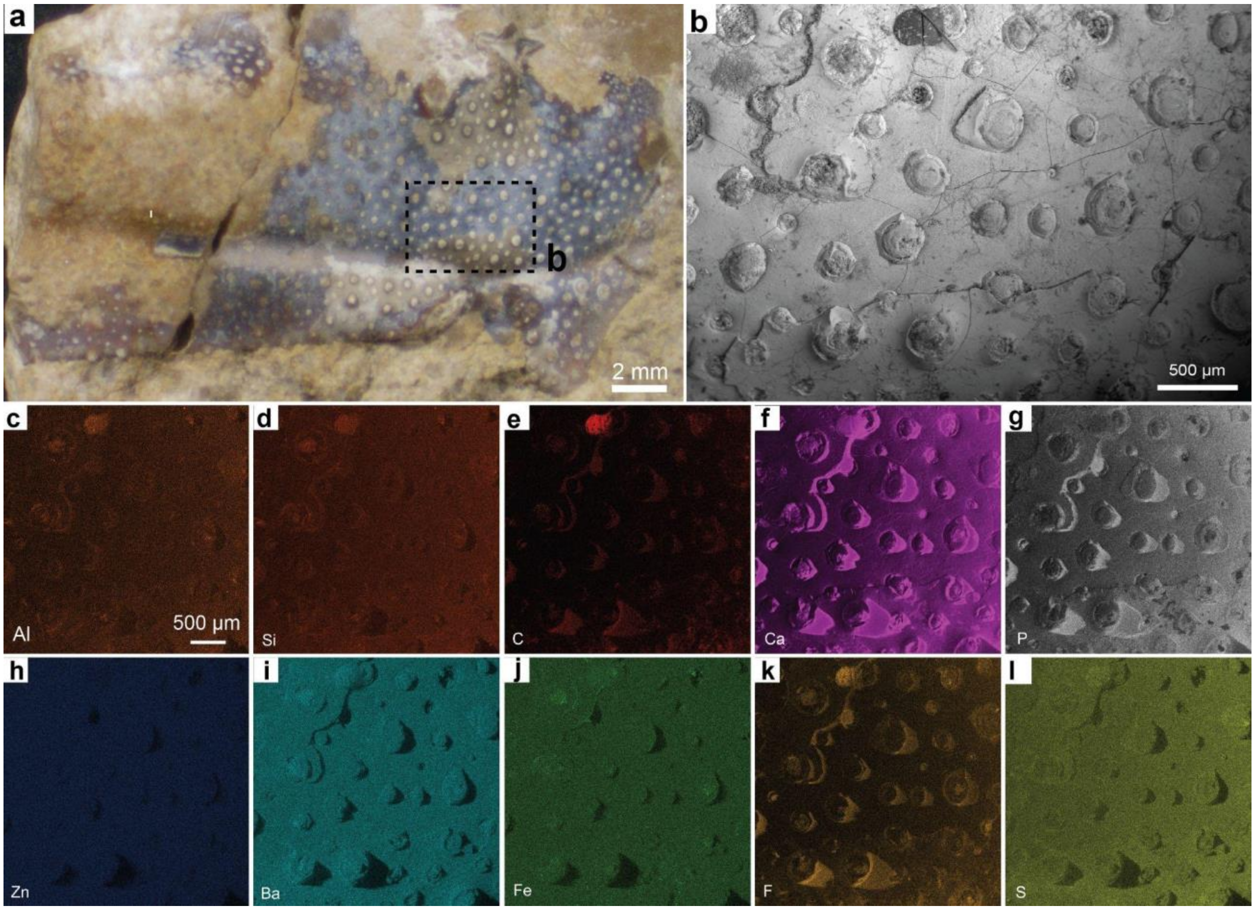

Supplementary Figure S22. Exoskeleton of a crustacean (BRLSI.M1243A) from the Strawberry Bank Lagerstätte (UK) in a limestone concretion. **a**, Reflected-light photograph of the specimen. **b**, Magnified BSE-SEM image of box in **a**, showing phosphatized carapace. **c-l**, EDS elemental maps of **b**.

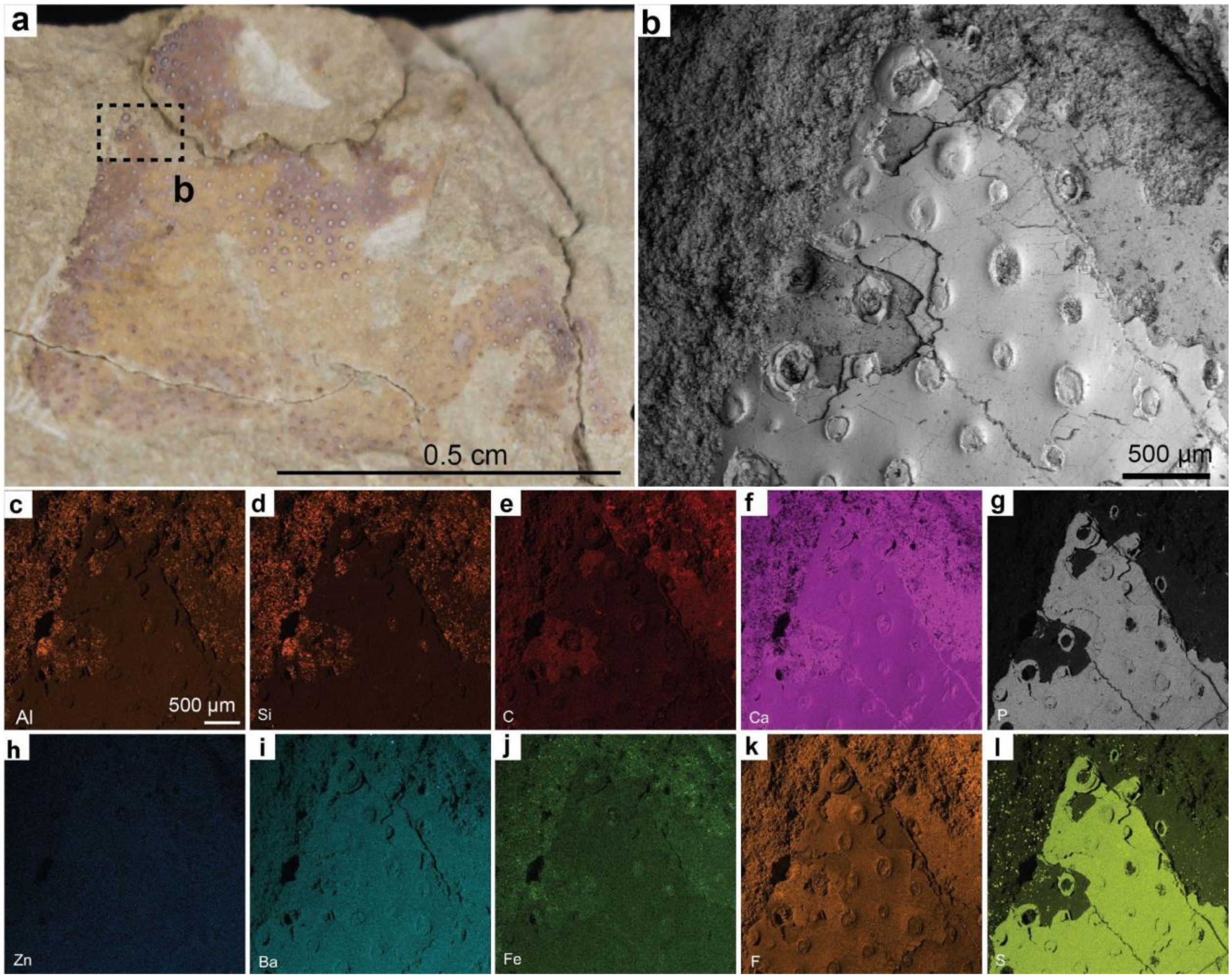

Supplementary Figure S23. Exoskeleton of a crustacean (BRLSI.M1243C) from the Strawberry Bank Lagerstätte (UK). **a**, Reflected-light photograph of the specimen. **b**, Magnified BSE-SEM image of box in **a**, showing phosphatized carapace. **c-l**, EDS elemental maps of **b**, showing the phosphorus and sulfur-rich exoskeleton in alumino-silicate rich limestone concretion.

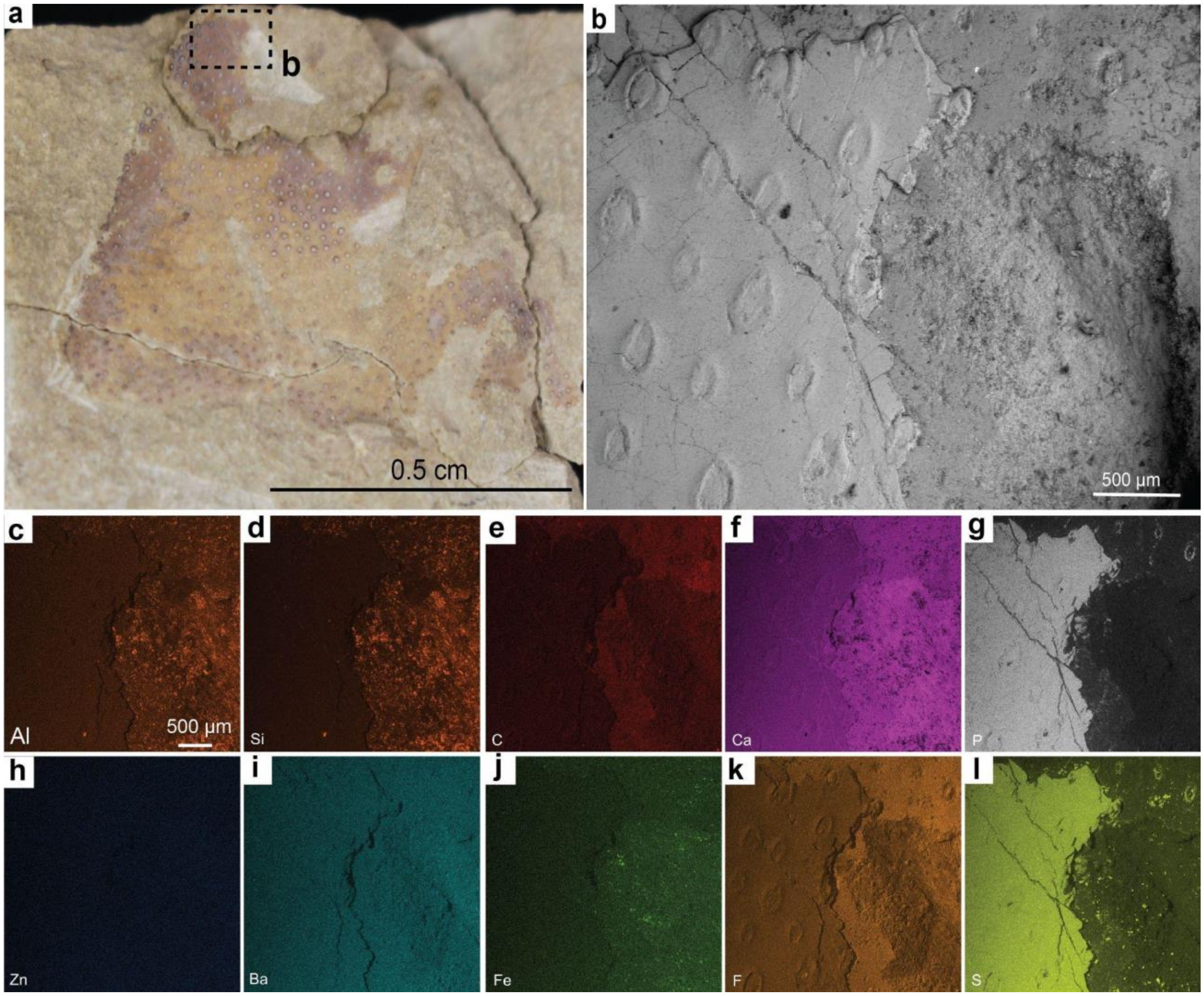

Supplementary Figure S24. Exoskeleton of a crustacean (BRLSI.M1243C) from the Strawberry Bank Lagerstätte (UK) in a limestone concretion. **a**, Reflected-light photograph of the specimen. **b**, Magnified BSE-SEM image of box in **a**, showing phosphatized carapace. **c-l**, EDS elemental maps of **b**, showing phosphorus and sulfur-rich exoskeleton in an alumino-silicate rich matrix.

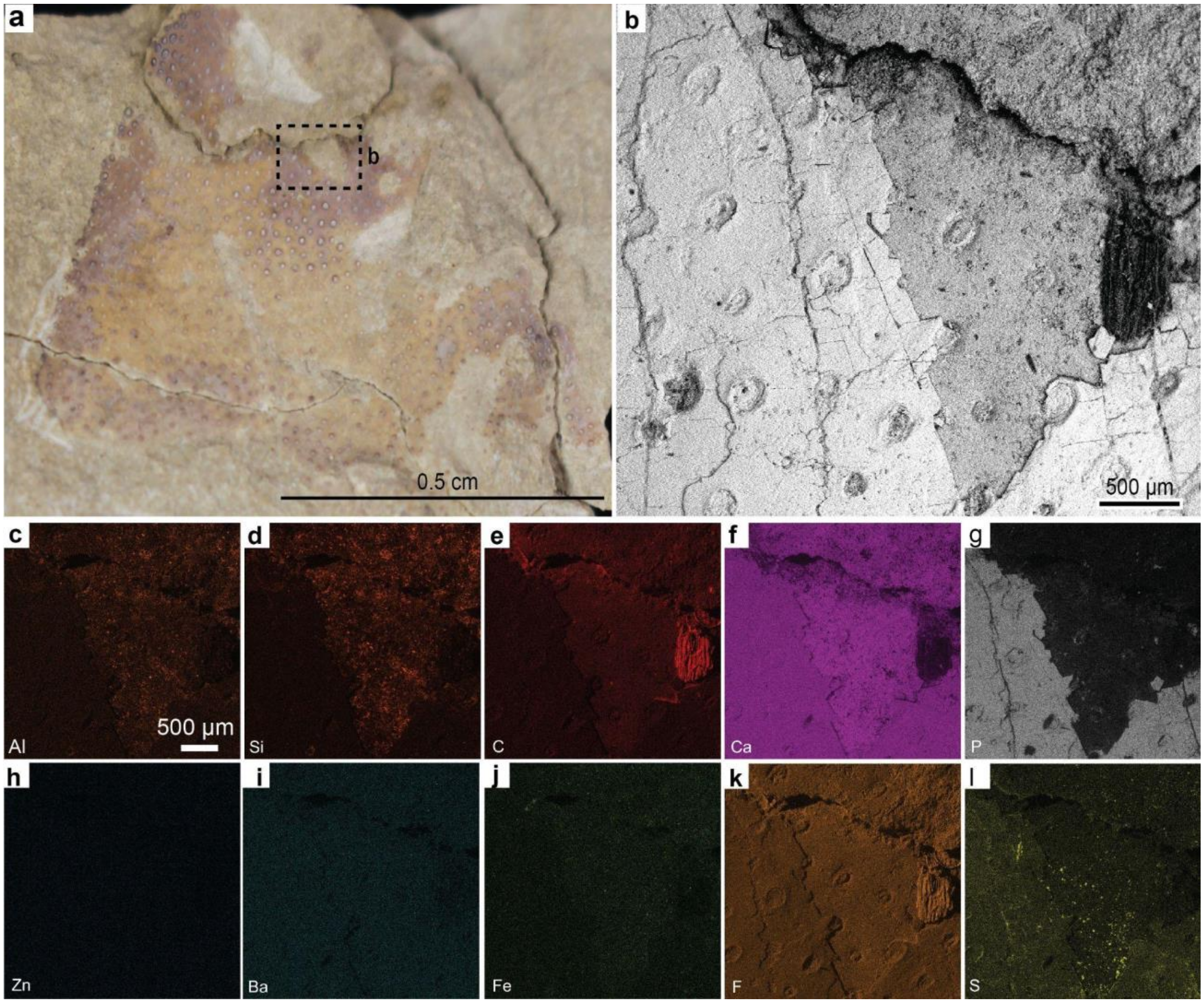

Supplementary Figure S25. Exoskeleton of a crustacean (BRLSI.M1243C) from the Strawberry Bank Lagerstätte (UK) in a limestone concretion. **a**, Reflected-light photograph of the specimen. **b**, Magnified BSE-SEM image of box in **a**, showing phosphatized carapace. **c-l**, EDS elemental maps of **b**.

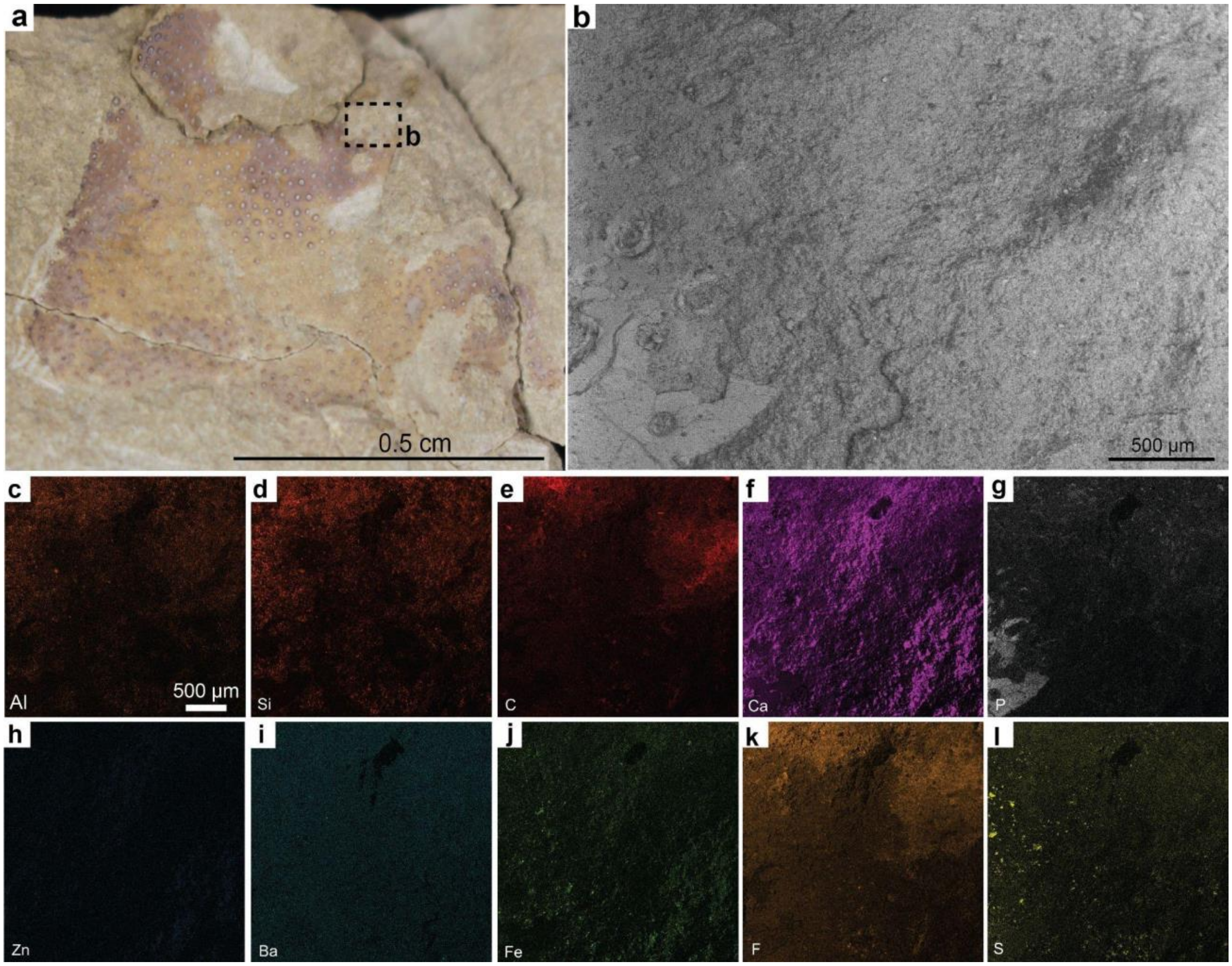

Supplementary Figure S26. Exoskeleton of a crustacean (BRLSI.M1243C) from the Strawberry Bank Lagerstätte (UK) in a limestone concretion. **a**, Reflected-light photograph of the specimen. **b**, Magnified BSE-SEM image of box in **a**, showing phosphatized carapace and aluminosilicate rich matrix. **c-l**, EDS elemental maps of **b**.

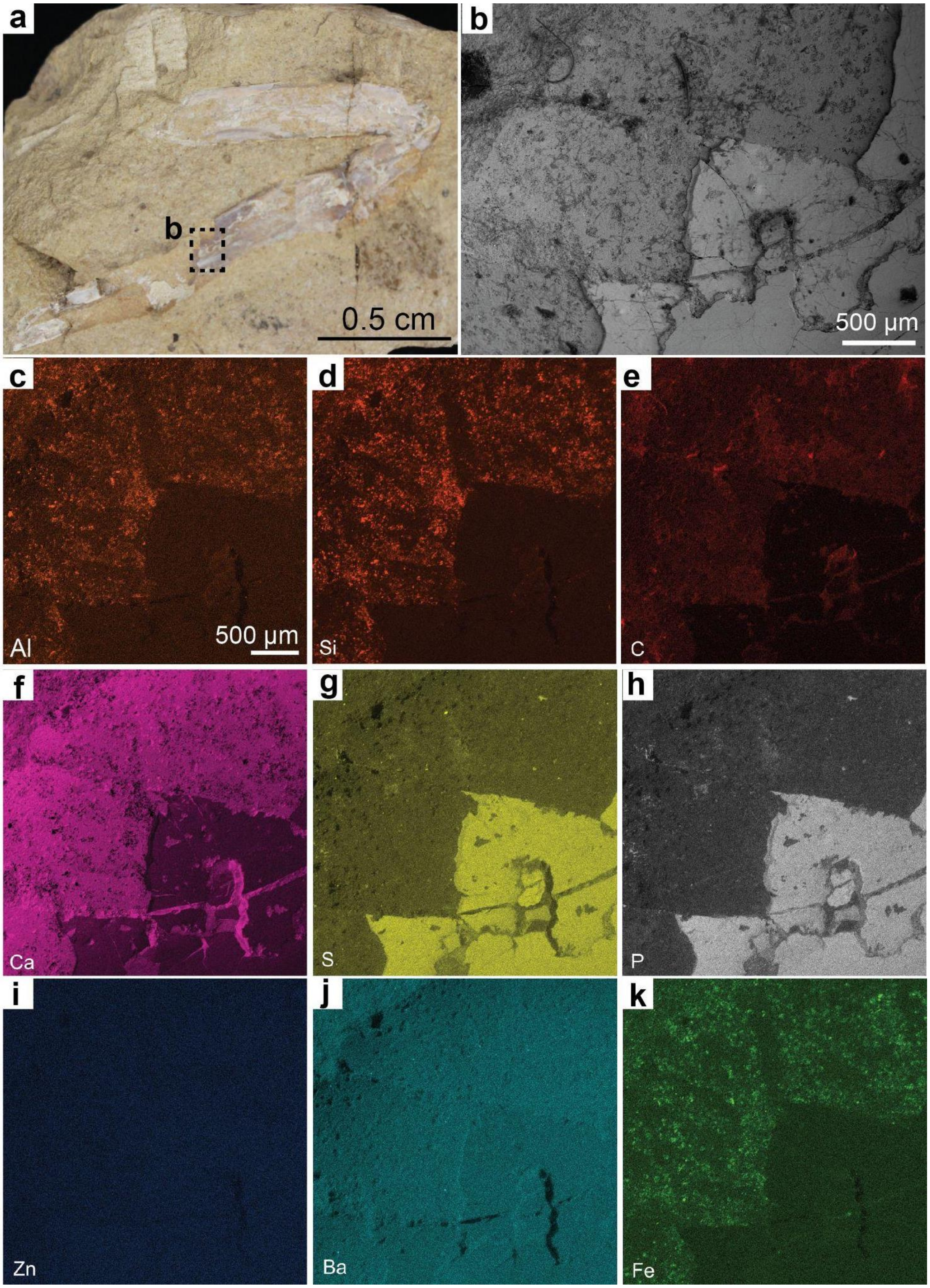

Supplementary Figure S27. Exoskeleton of a crustacean (BRLSI.M1245) from the Strawberry Bank Lagerstätte (UK) in a limestone concretion. **a**, Reflected-light photograph of the specimen. **b**, Magnified BSE-SEM image of box in **a**. **c-l**, EDS elemental maps of **b** showing high concentrations of calcium, phosphorus and sulfur in the carapace.

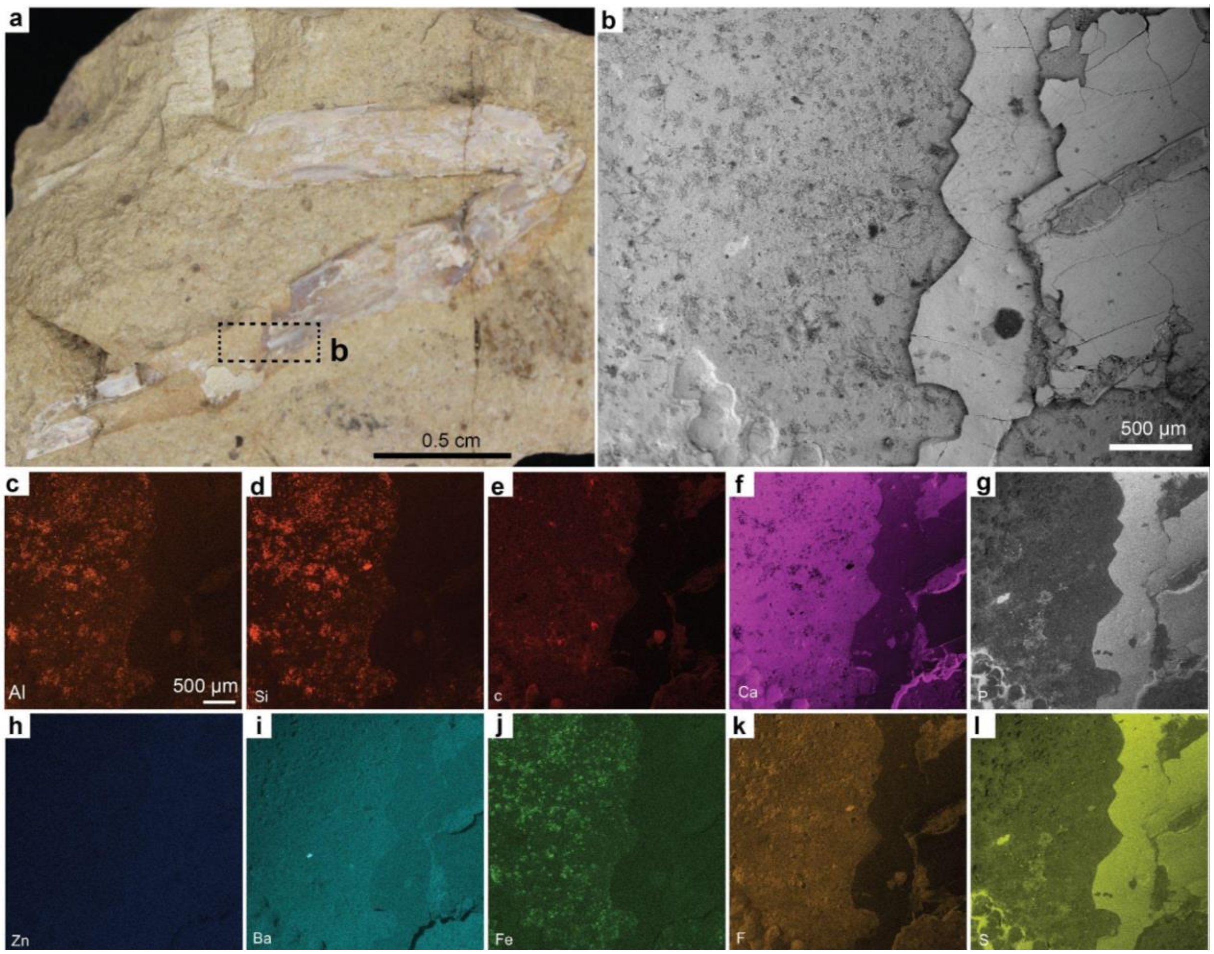

Supplementary Figure S28. Exoskeleton of a crustacean (BRLSI.M1245) from the Strawberry Bank Lagerstätte (UK) in a limestone concretion. **a**, Reflected-light photograph of the specimen. **b**, Magnified BSE-SEM image of box in **a**. **c-l**, EDS elemental maps of **b** showing high concentrations of phosphorus and sulfur in the carapace and alumino-silicate in the surrounding matrix.

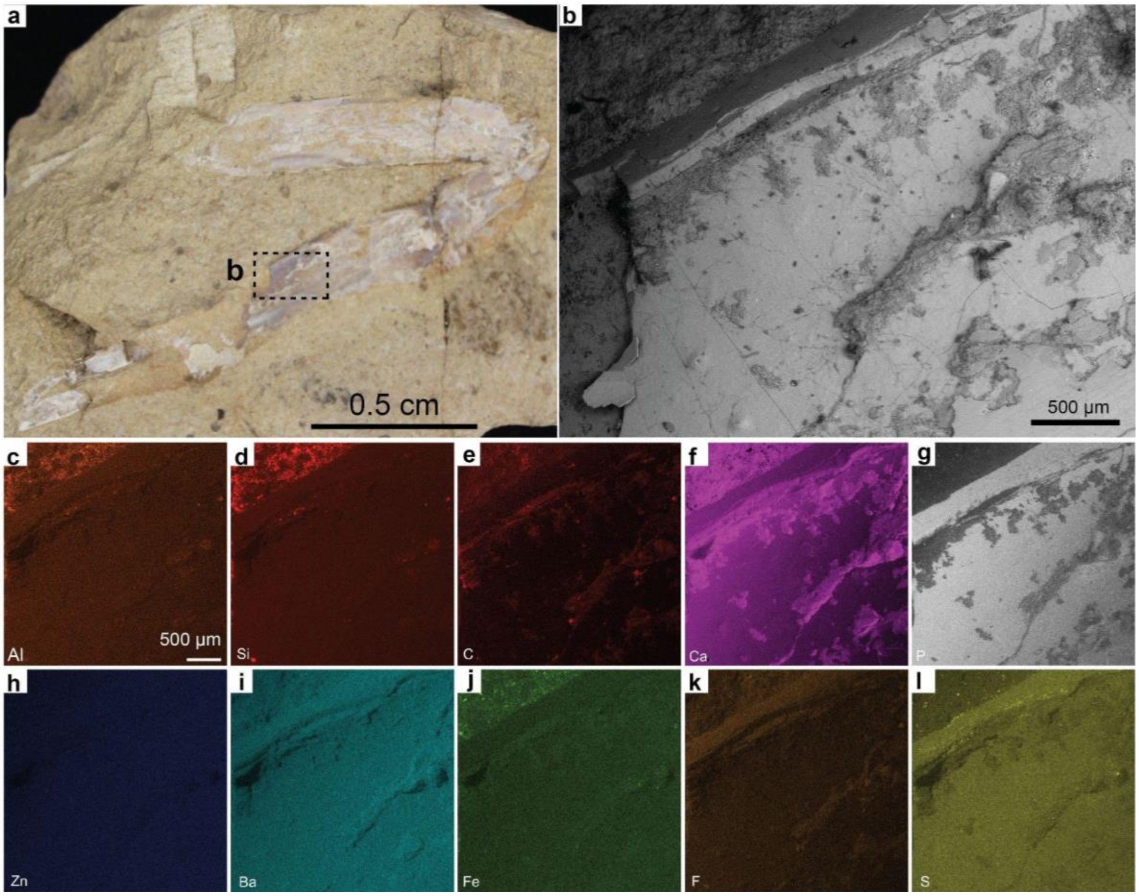

Supplementary Figure S29. Exoskeleton of a crustacean (BRLSI.M1245) from the Strawberry Bank Lagerstätte (UK) in a limestone concretion. **a**, Reflected-light photograph of the specimen. **b**, Magnified BSE-SEM image of box in **a**, showing phosphatized carapace. **c-l**, EDS elemental maps of **b** showing phosphorus-rich exoskeleton.

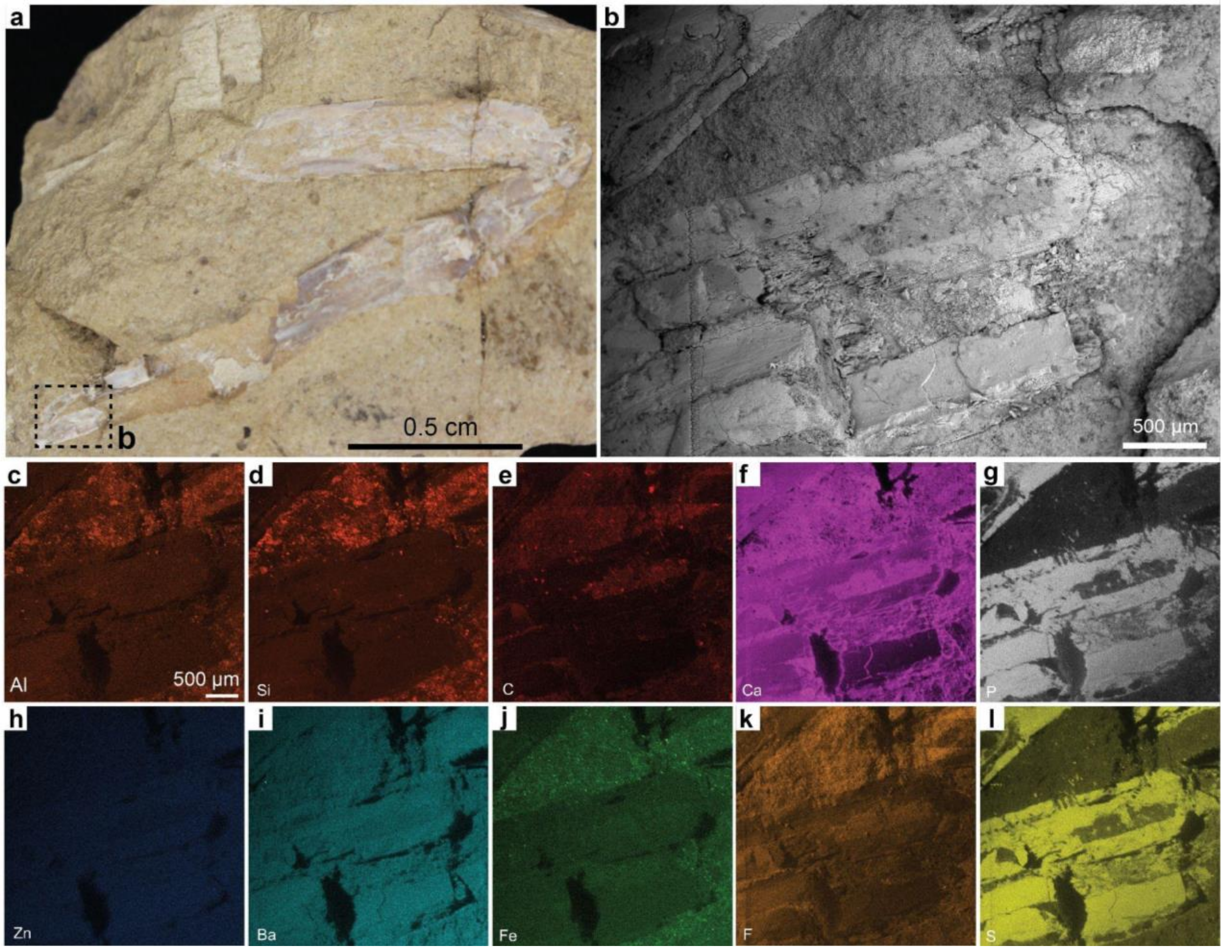

Supplementary Figure S30. Exoskeleton of a crustacean (BRLSI.M1245) from the Strawberry Bank Lagerstätte (UK) in a limestone concretion. **a**, Reflected-light photograph of the specimen. **b**, Magnified BSE-SEM image of box in **a**, showing phosphatized carapace. **c-l**, EDS elemental maps of **b**.

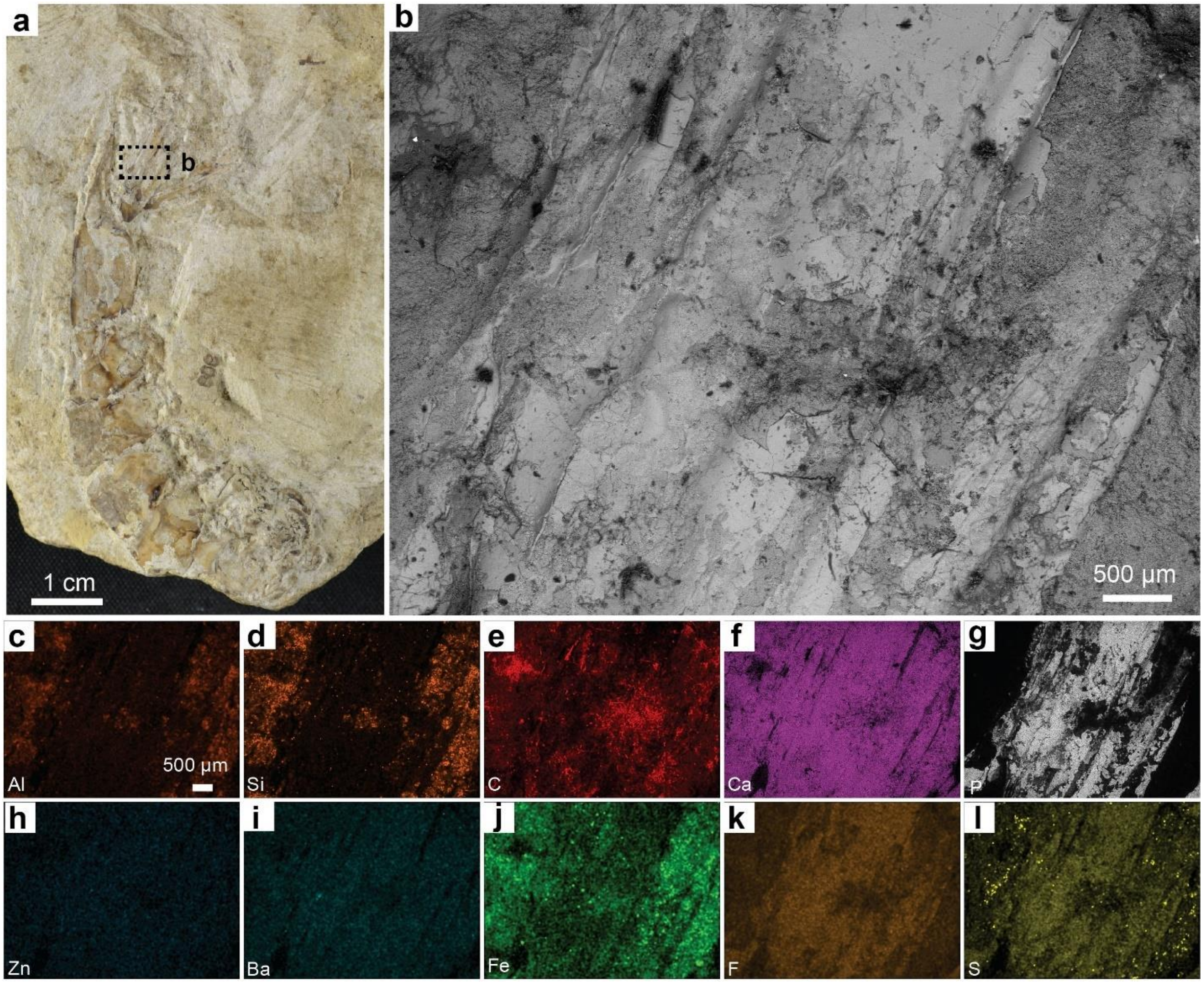

Supplementary Figure S31. Exoskeleton of a prawn (BRLSI.M1256) from the Strawberry Bank Lagerstätte (UK) in a limestone concretion. **a**, Reflected-light photograph of specimen. **b**, Magnified BSE-SEM image of box in **a**, showing phosphatized carapace. **c-l**, EDS elemental maps of **b**.

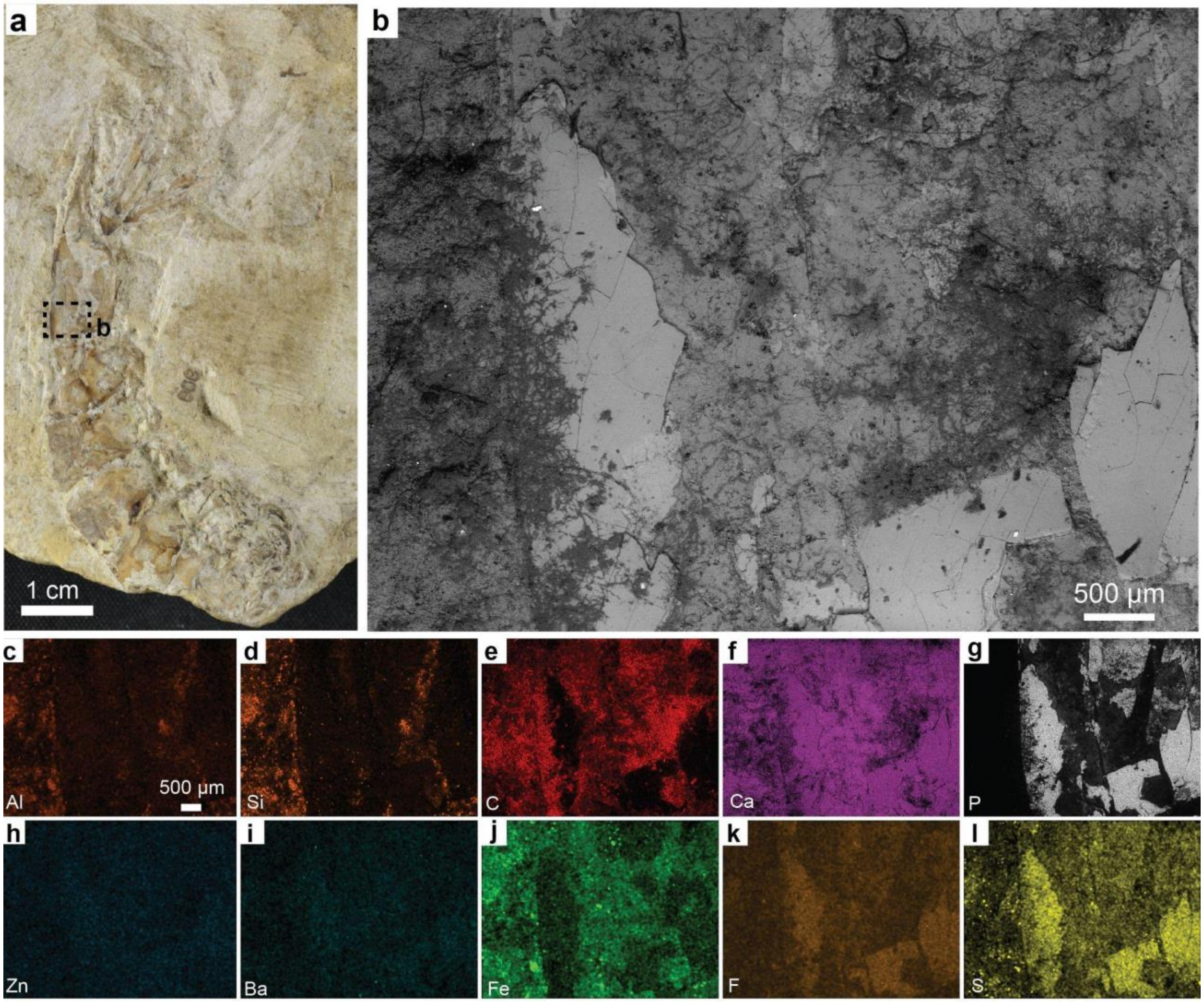

Supplementary Figure S32. Exoskeleton of a prawn (BRLSI.M1256) from the Strawberry Bank Lagerstätte (UK) in a limestone concretion. **a**, Reflected-light photograph of the specimen. **b**, Magnified BSE-SEM image of box in **a**, showing phosphatized carapace. **c-l**, EDS elemental maps of **b**.

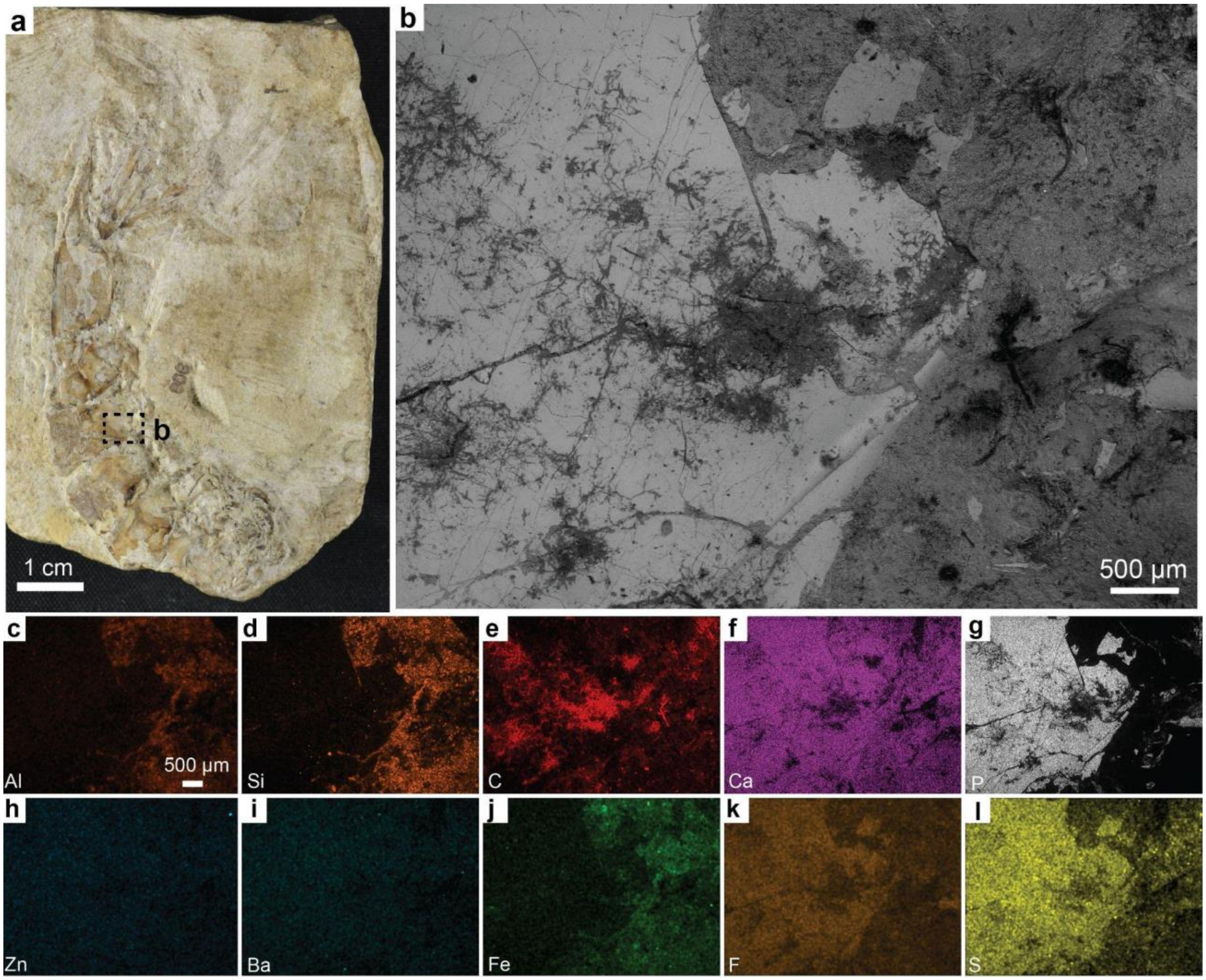

Supplementary Figure S33. Exoskeleton of a prawn (BRLSI.M1256) from the Strawberry Bank Lagerstätte (UK) in a limestone concretion. **a**, Reflected-light photograph of the specimen. **b**, Magnified BSE-SEM image of box in **a**, showing phosphatized carapace. **c-l**, EDS elemental maps of **b**.

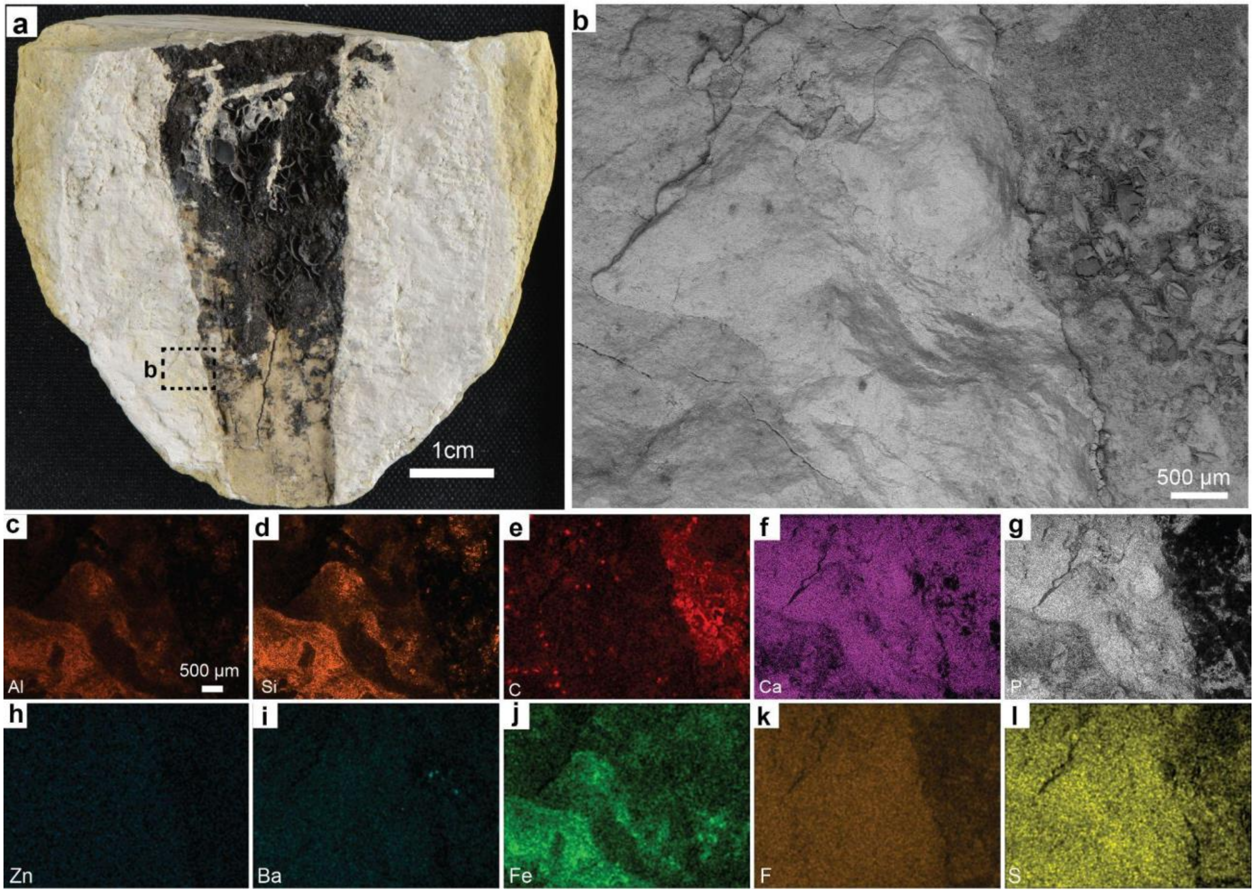

Supplementary Figure S34. Vampyropod specimen (BRLSI.M3917) from the Strawberry Bank Lagerstätte (UK) in a limestone concretion. **a**, Reflected-light photograph of the specimen. **b**, Magnified BSE-SEM image of box in **a**. **c-l**, EDS elemental maps of **b**, showing phosphatized mantle tissue and carbonaceous ink sac.

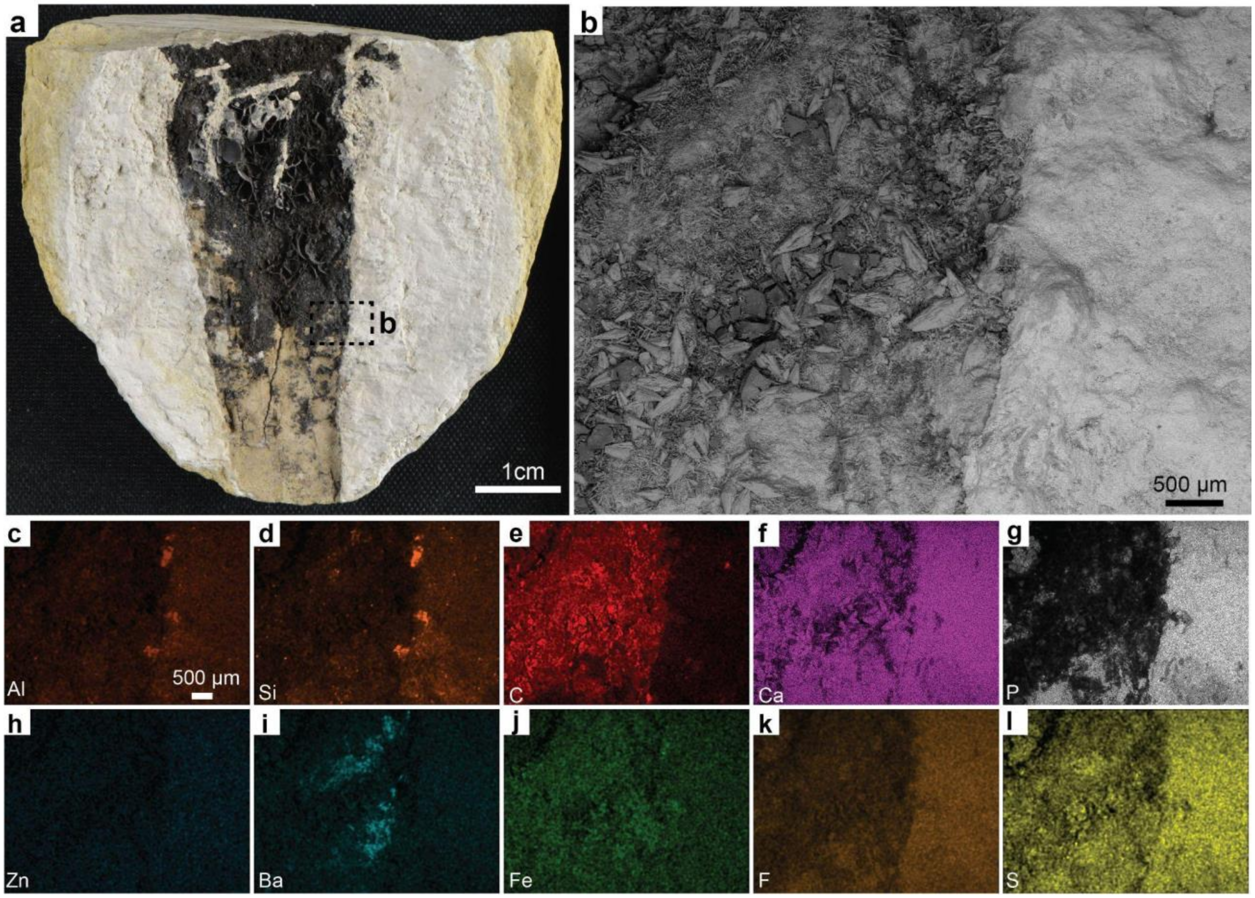

Supplementary Figure S35. Vampyropod specimen (BRLSI.M3917) from the Strawberry Bank Lagerstätte (UK) in a limestone concretion. **a**, Reflected-light photograph of the specimen. **b**, Magnified BSE-SEM image of box in **a**. **c-l**, EDS elemental maps of **b**, showing phosphatized mantle tissue and carbonaceous ink sac with minor barite filling voids in between the fossilized tissues.

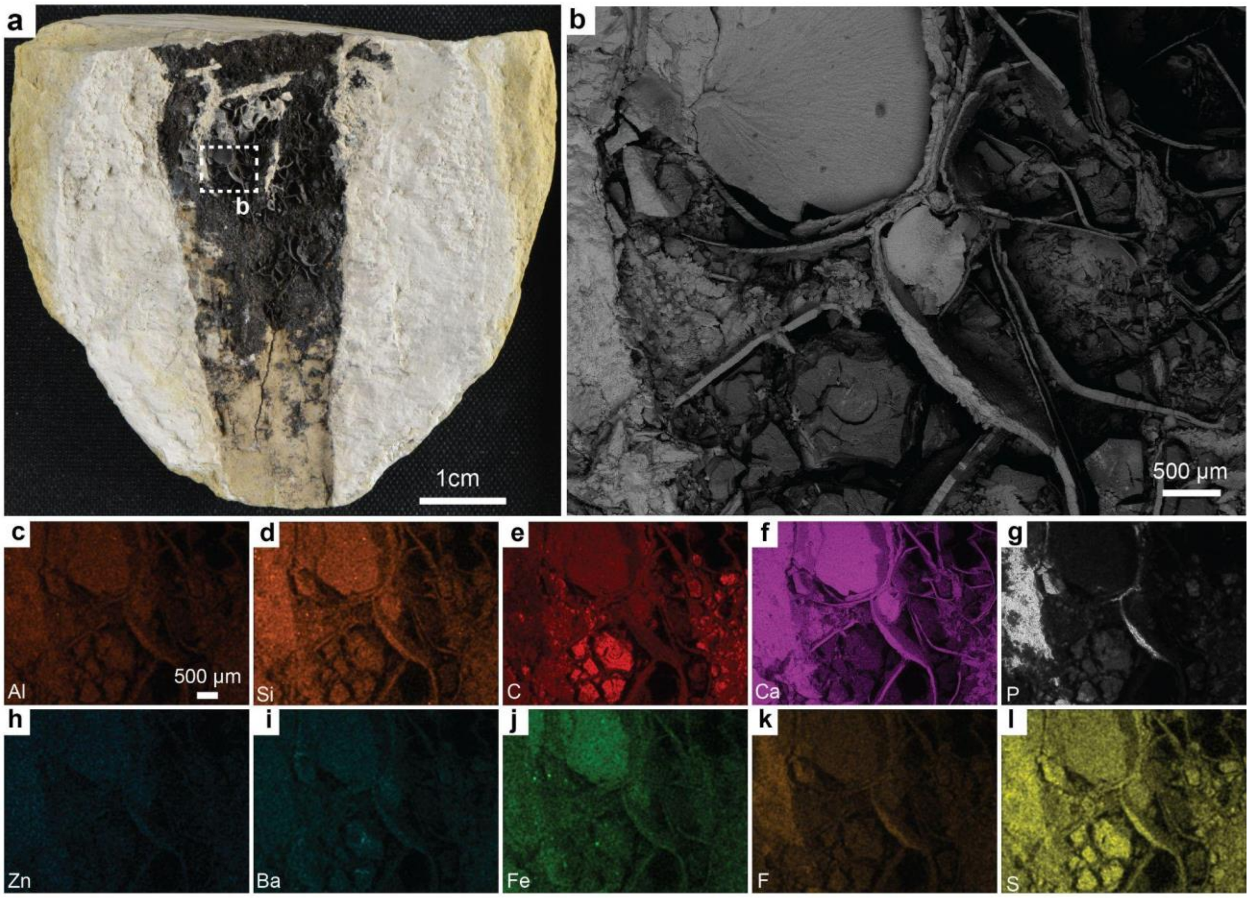

Supplementary Figure S36. Vampyropod specimen (BRLSI.M3917) from the Strawberry Bank Lagerstätte (UK) in a limestone concretion. **a**, Reflected-light photograph of the specimen. **b**, Magnified BSE-SEM image of box in **a**. **c-l**, EDS elemental maps of b, showing phosphatized mantle tissue and carbonaceous ink sac.

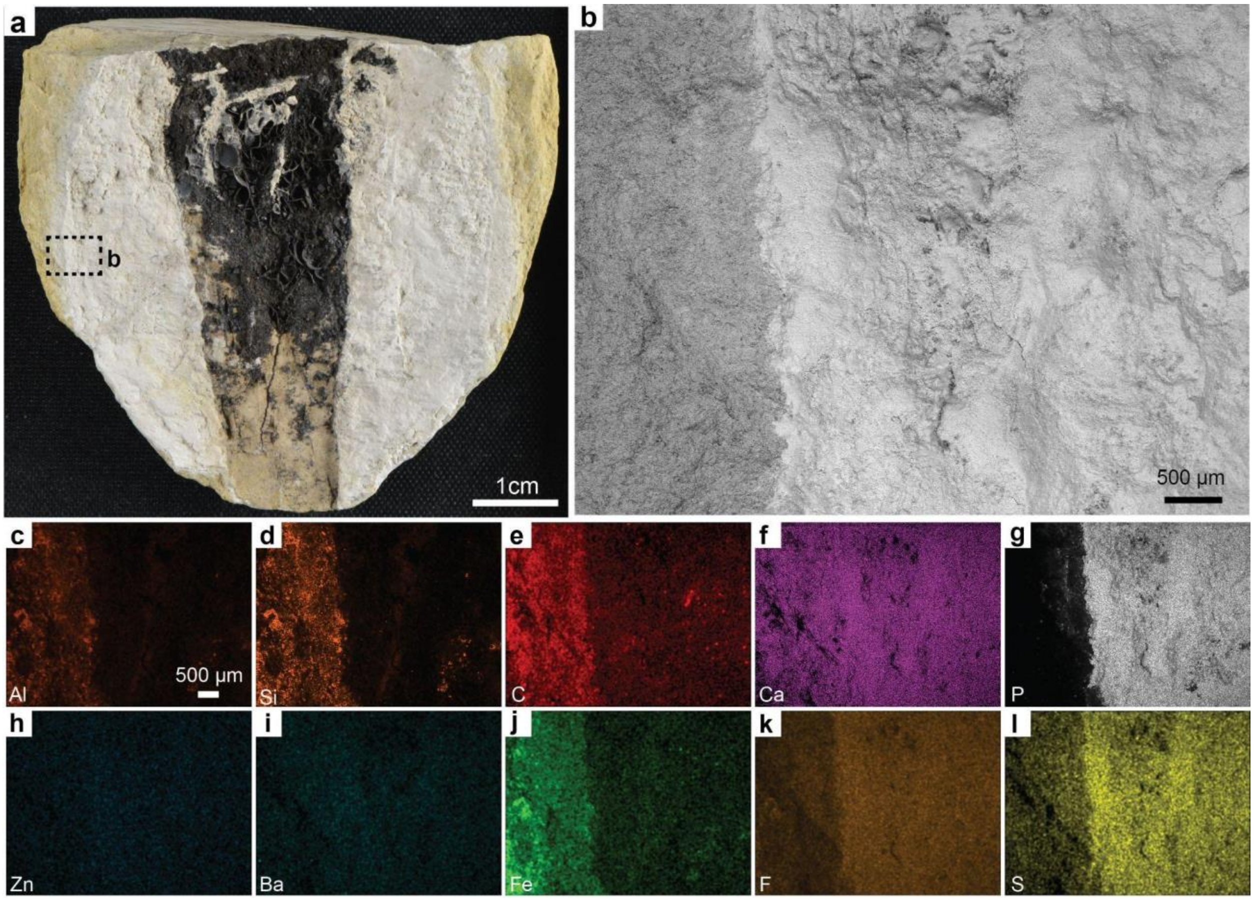

Supplementary Figure S37. Vampyropod specimen (BRLSI.M3917) from the Strawberry Bank Lagerstätte (UK) in a limestone concretion. **a**, Reflected-light photograph of the specimen. **b**, Magnified BSE-SEM image of box in **a**, showing the phosphatized mantle tissue. **c-l**, EDS elemental maps of **b**, showing phosphatized mantle tissue and carbonaceous ink sac.

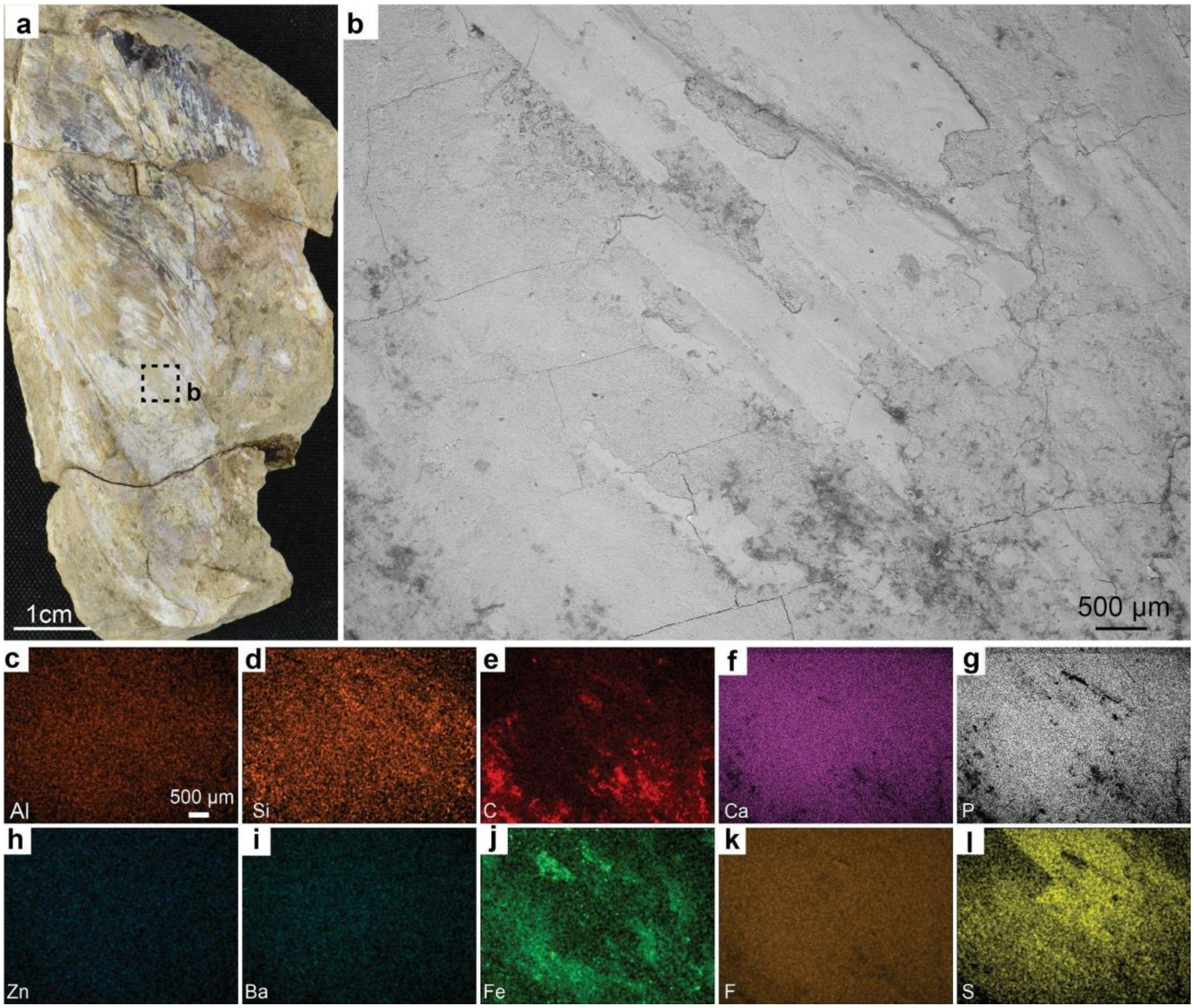

Supplementary Figure S38. Coleoid gladius (BRLSI.M1237 B) from the Strawberry Bank Lagerstätte (UK) in a limestone concretion. **a**, Reflected-light photograph of the specimen. **b**, Magnified BSE-SEM image of box in **a**, showing phosphatized gladius. **c-l**, EDS elemental maps of **b**.

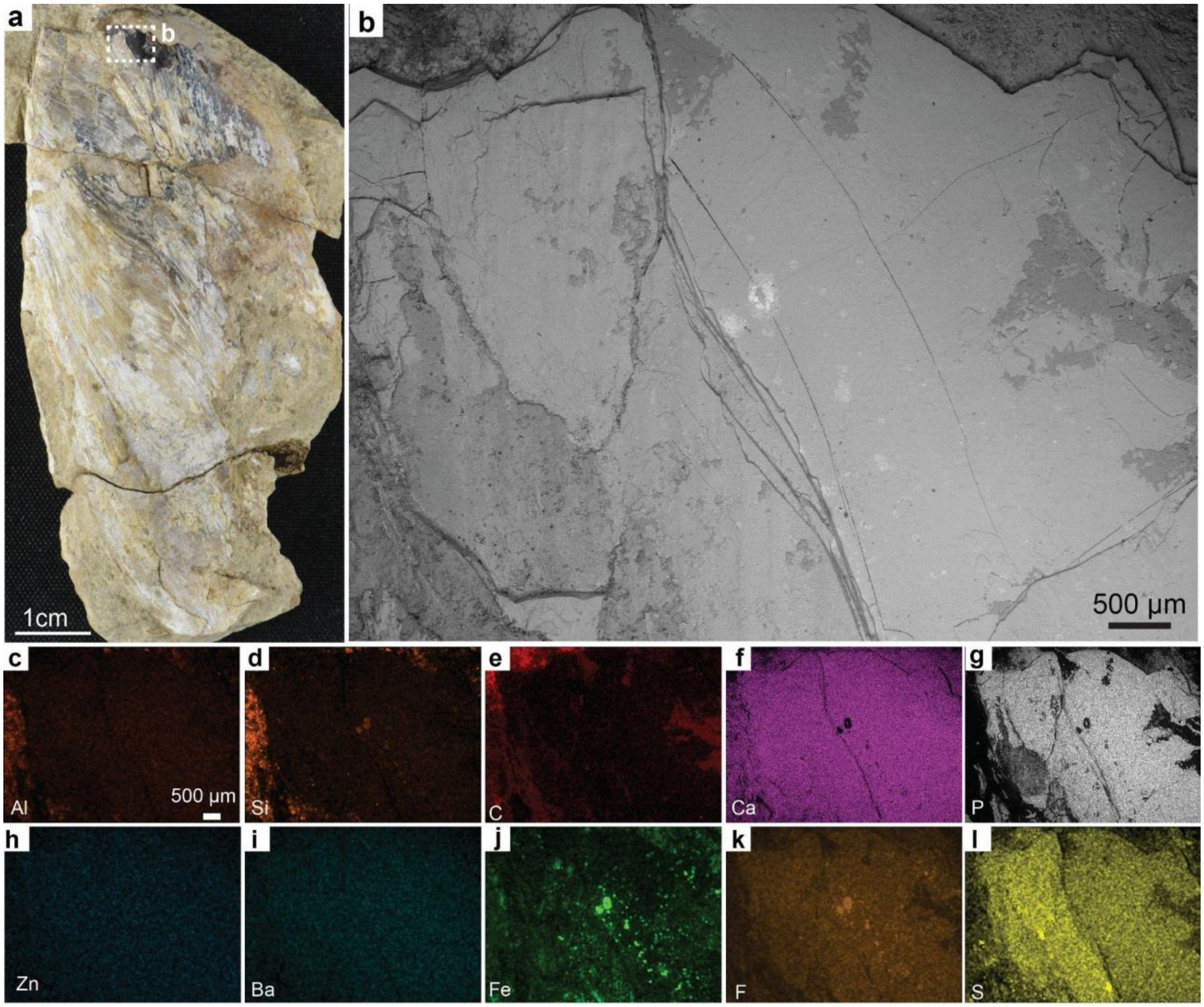

Supplementary Figure S39. Coleoid gladius (BRLSI.M1237 B) from the Strawberry Bank Lagerstätte (UK) in a limestone concretion. **a**, Reflected-light photograph of the specimen. **b**, Magnified BSE-SEM image of box in **a**, showing phosphatized gladius. **c-l**, EDS elemental maps of **b**.

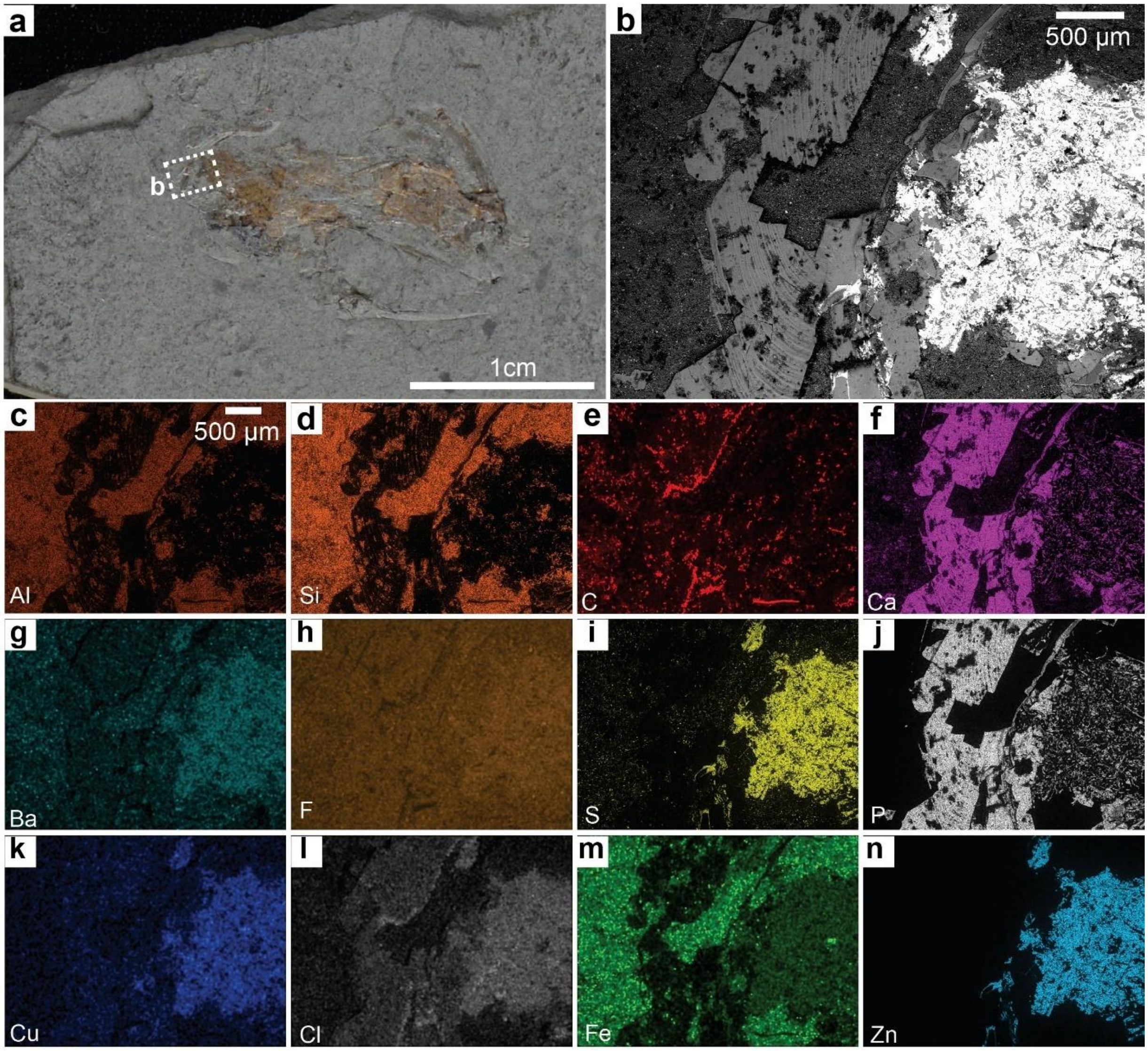

Supplementary Figure S40. Fish fossil (NPL00036036.000) from the Kromer Quarry of the Posidonia Shale Lagerstätte (Germany). **a**, Reflected-light photograph of the specimen. **b**, BSE-SEM image of the specimen. **c-n**, Elemental maps of **b** showing phosphatic skeletal material and sulfide minerals: pyrite and sphalerite.

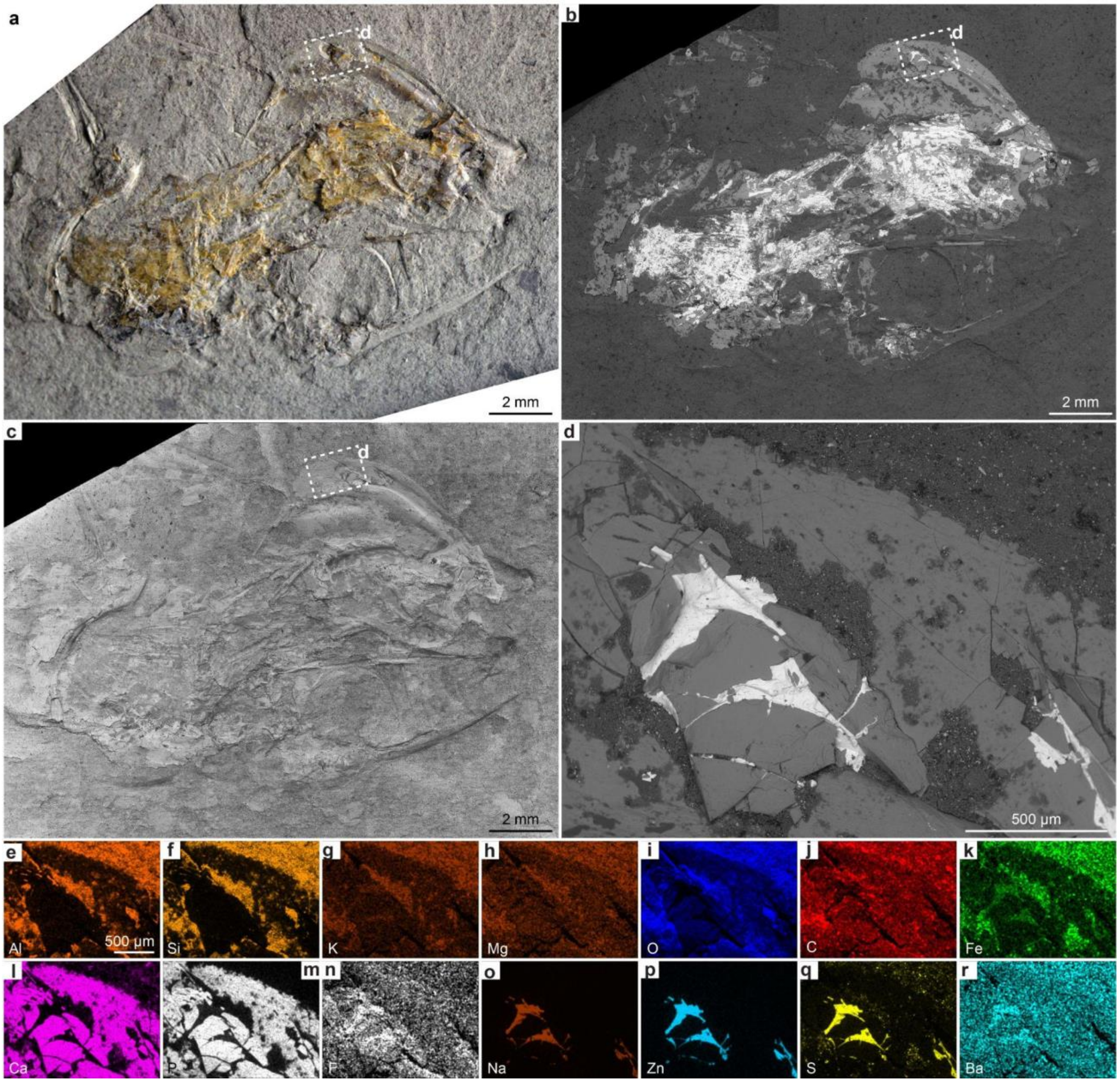

Supplementary Figure S41. Fish fossil (NPL00036036.000) from the Kromer Quarry of the Posidonia Shale Lagerstätte (Germany). **a**, Reflected-light photograph of the specimen. **b**, Mosaic BSE-SEM image of specimen. **c**, Mosaic SE-SEM image of specimen. **d**, Magnified BSE-SEM image of boxes in **a-c**, showing phosphatic skeletal material and sulfide minerals: pyrite and sphalerite. **e-r**, EDS elemental maps of **d**.

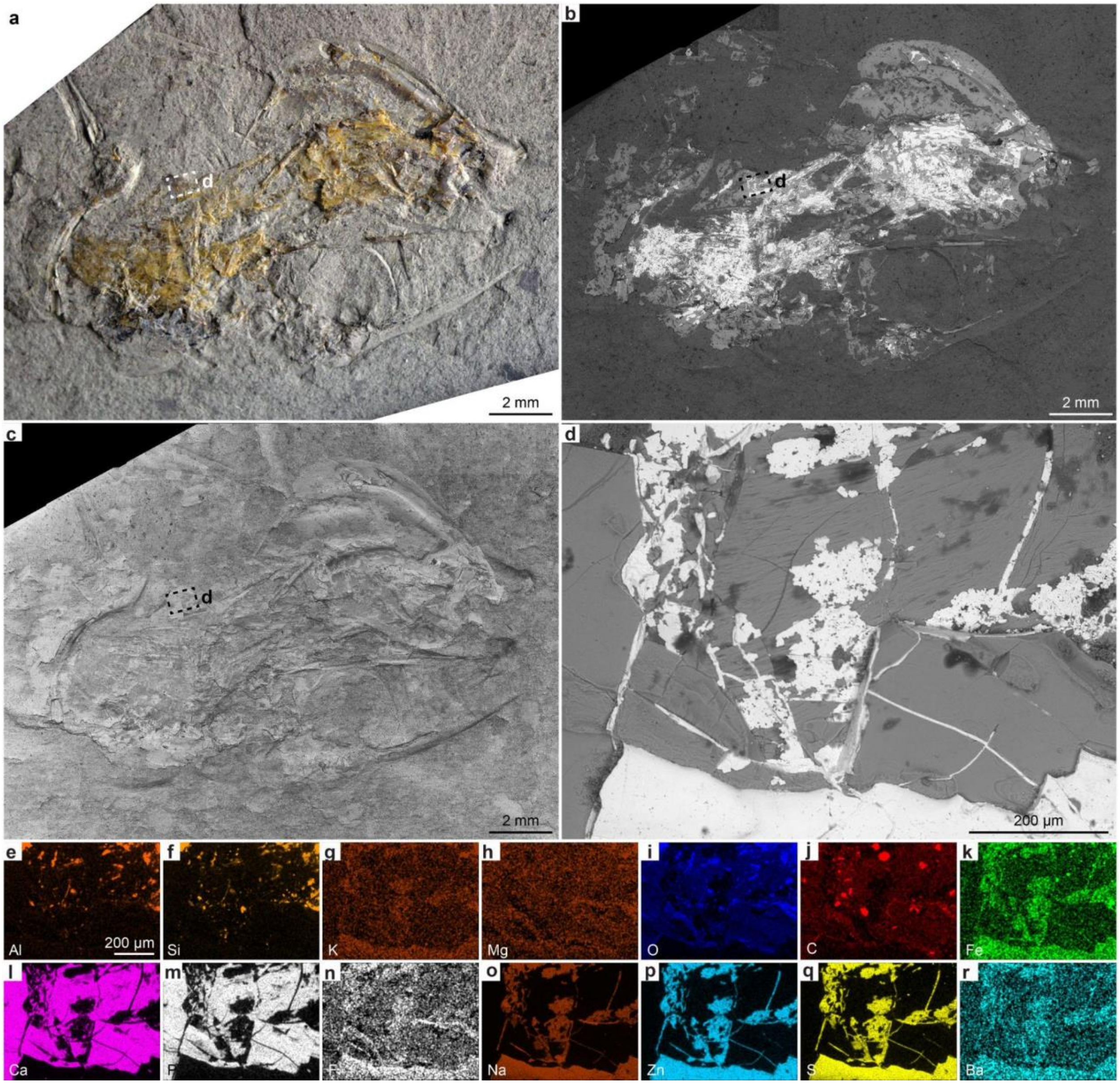

Supplementary Figure S42. Fish fossil (NPL00036036.000) from the Kromer Quarry of the Posidonia Shale Lagerstätte (Germany) with crack-filling sulfide minerals. **a**, Reflected-light photograph of specimen. **b**, Mosaic BSE-SEM image of specimen. **c**, Mosaic SE-SEM image of specimen. **d**, Magnified BSE-SEM image of boxes in **a-c**, showing phosphatic skeletal material and sulfide minerals: pyrite and sphalerite. **e-r**, EDS elemental maps of **d**.

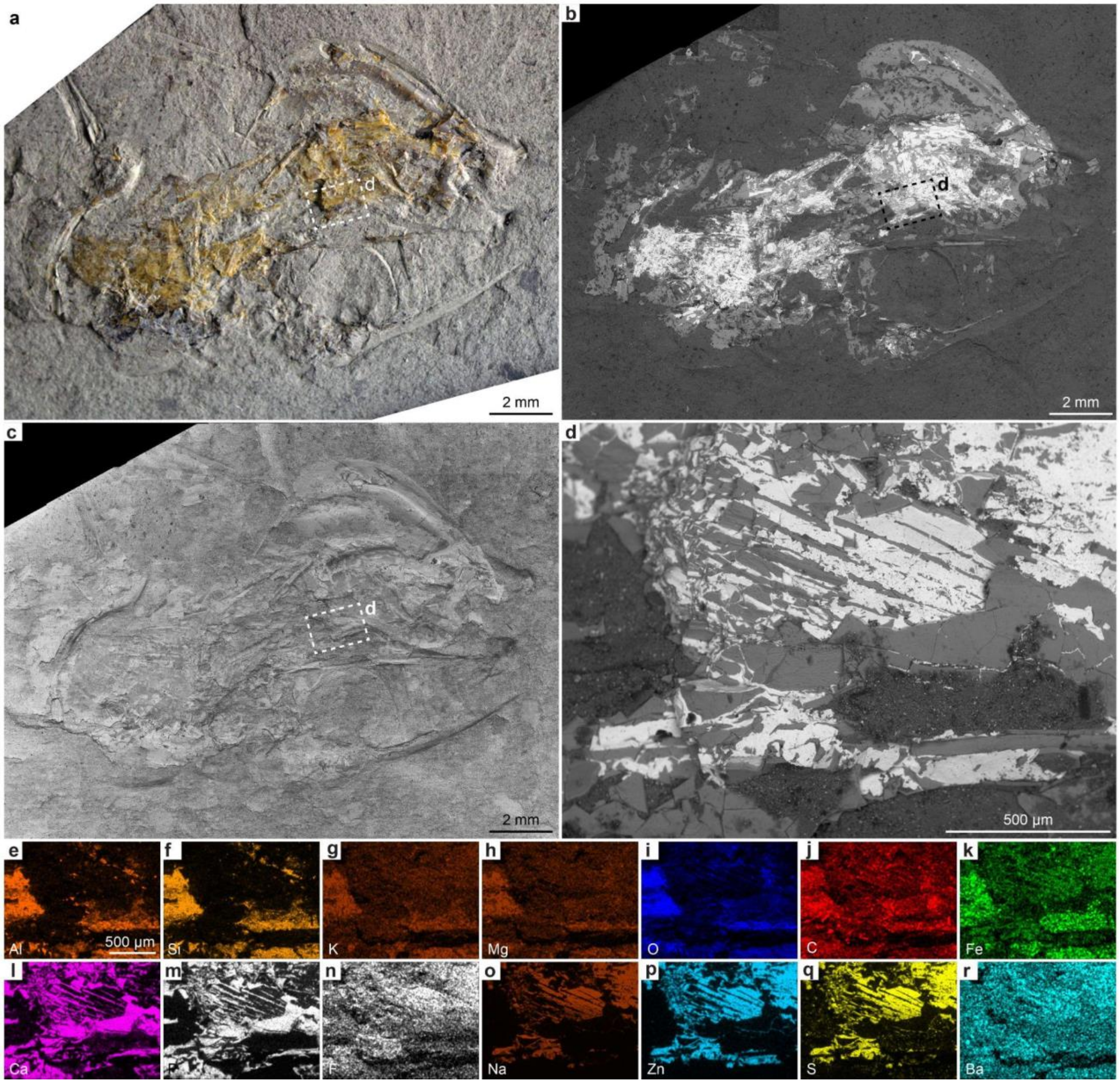

Supplementary Figure S43. Fish fossil (NPL00036036.000) from the Kromer Quarry of the Posidonia Shale Lagerstätte (Germany) with sulfide minerals. **a**, Reflected-light photograph of the specimen. **b**, Mosaic BSE-SEM image of specimen. **c**, Mosaic SE-SEM image of specimen. **d**, Magnified BSE-SEM image of boxes in **a-c**, showing phosphatic skeletal material and sulfide minerals: pyrite and sphalerite. **e-r**, EDS elemental maps of **d**.

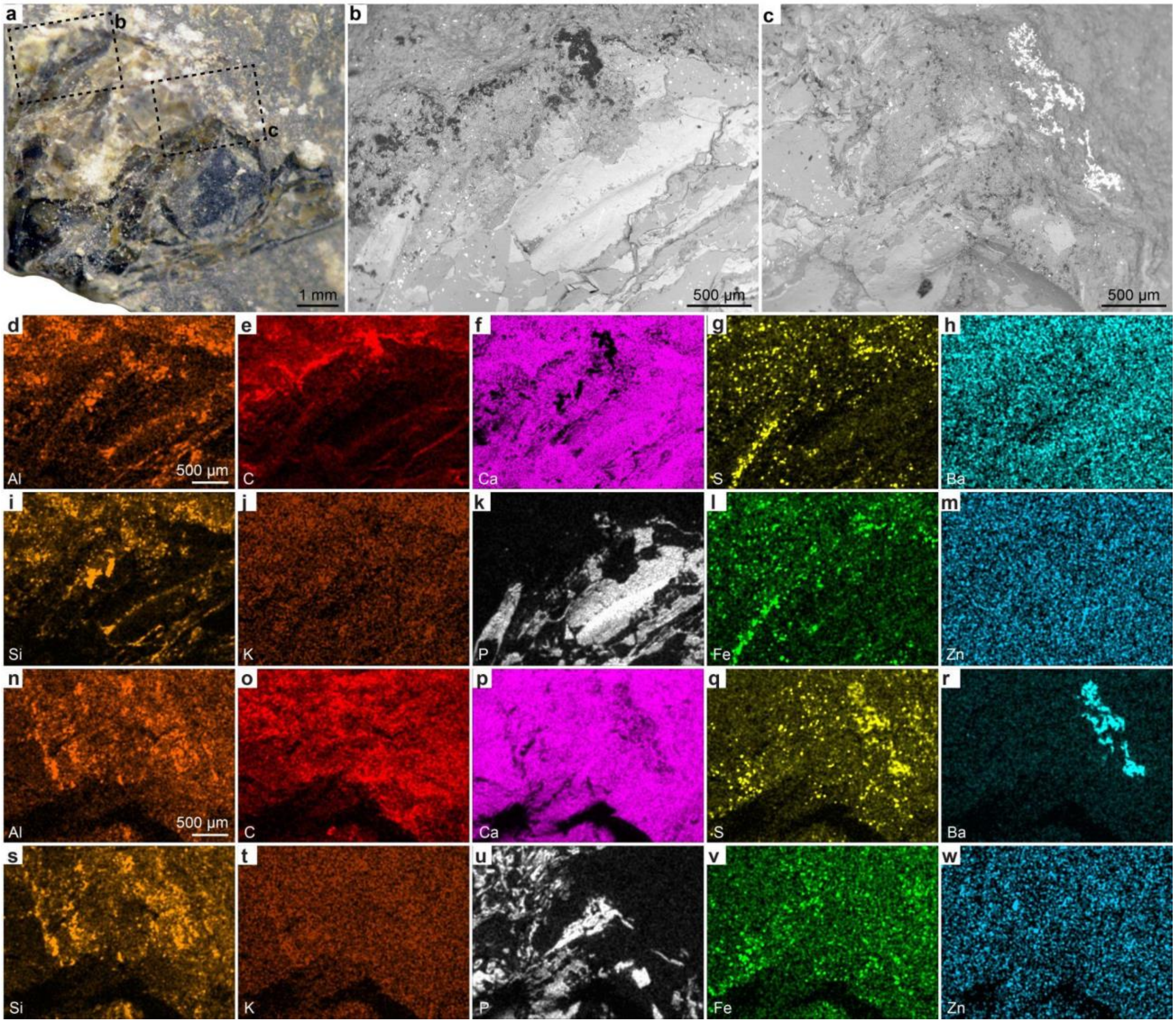

Supplementary Figure S44. Fish skull (NPL00094461.000) from the Posidonia Shale Lagerstätte at Dormettingen near Dotternhausen, Germany. **a**, Reflected-light photograph of the specimen. **b-c**, BSE-SEM images of the specimen. **b**, Magnified view of box in a, showing phosphatic skull material and euhedral, subhedral, and framboidal pyrite. **c**, Magnified view of box in **a**, showing phosphatic skull material along with barite in the shale matrix around the fossil. **d-m**, EDS elemental maps of **b**. **n-w,** EDS elemental maps of **c**.

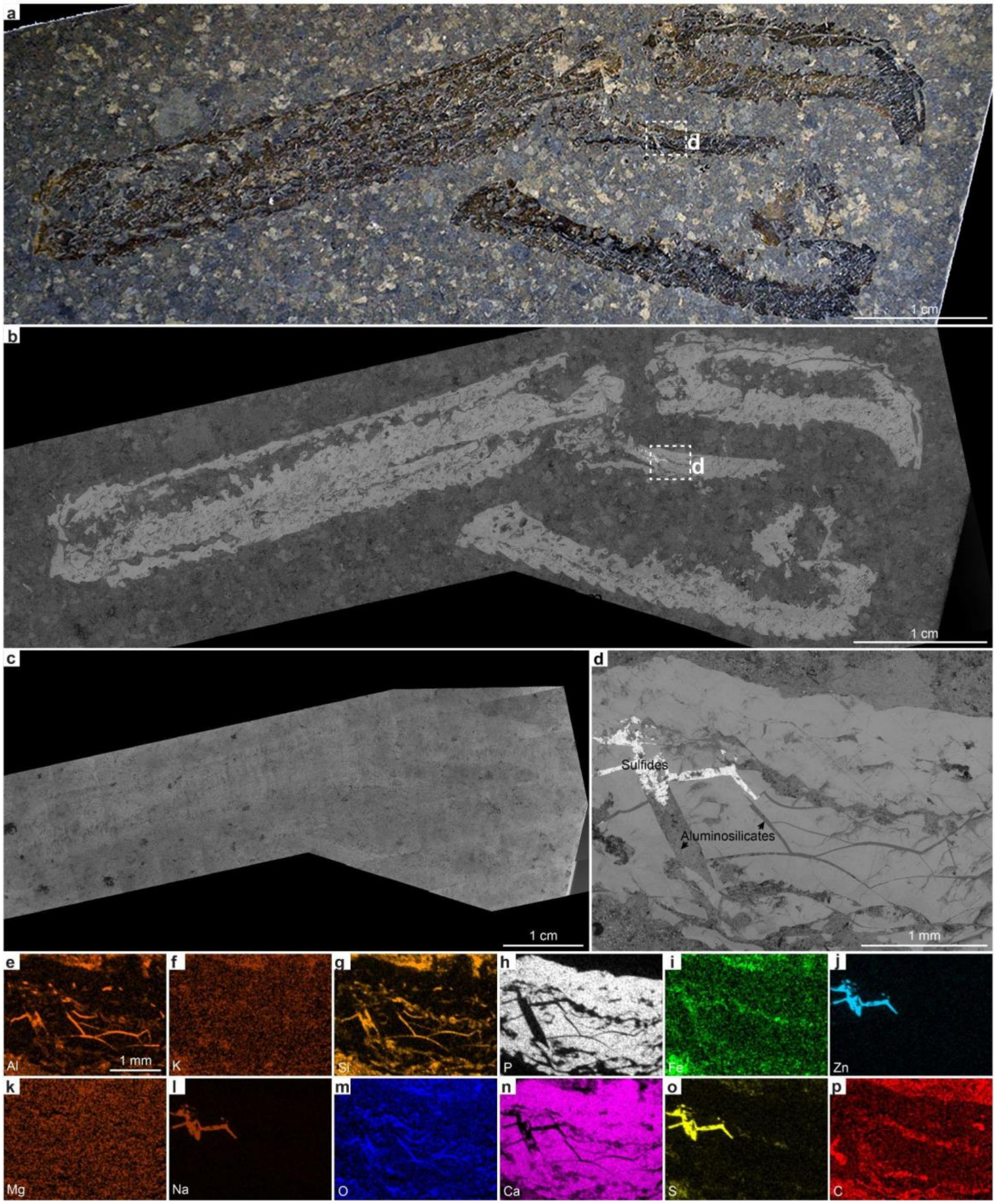

Supplementary Figure S45. Claw of *Uncina posidoniae* (NPL00036039.000) from the Posidonia Shale Lagerstätte (Fleins layer; Germany) prepared as a polished slab. **a**, Reflected-light photograph of the specimen. **b**, Mosaic BSE-SEM image of specimen. **c**, Mosaic SE-SEM image (with limited contrast due to lack of topographic relief). **d**, Magnified BSE-SEM image of boxes in **a** and **b**, showing phosphatic carapace, containing crack- and void-filling sulfide (i.e., pyrite and sphalerite) and aluminosilicate minerals, surrounded by shale matrix. **e-p**, EDS elemental maps of **d**.

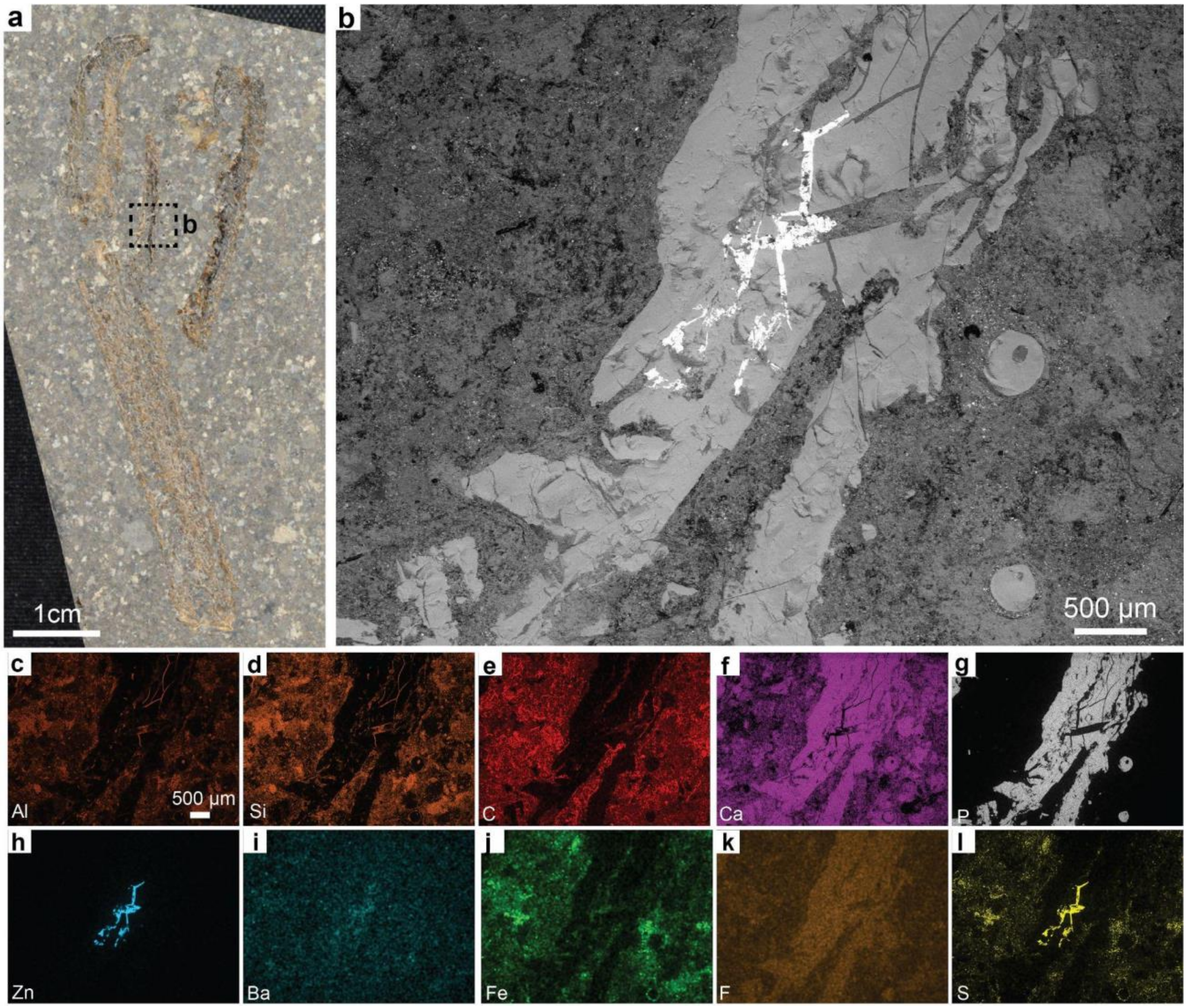

Supplementary Figure S46. Claw of *Uncina posidoniae* (NPL00036039.000) from the Posidonia Shale Lagerstätte (Fleins layer; Germany) prepared as a polished slab. **a**, Reflected-light photograph of the specimen. **b**, Magnified BSE-SEM image of the box in **a**. **c-l**, EDS elemental maps of **b** showing phosphatized carapace, void filling sphalerite in a shale-rich matrix.

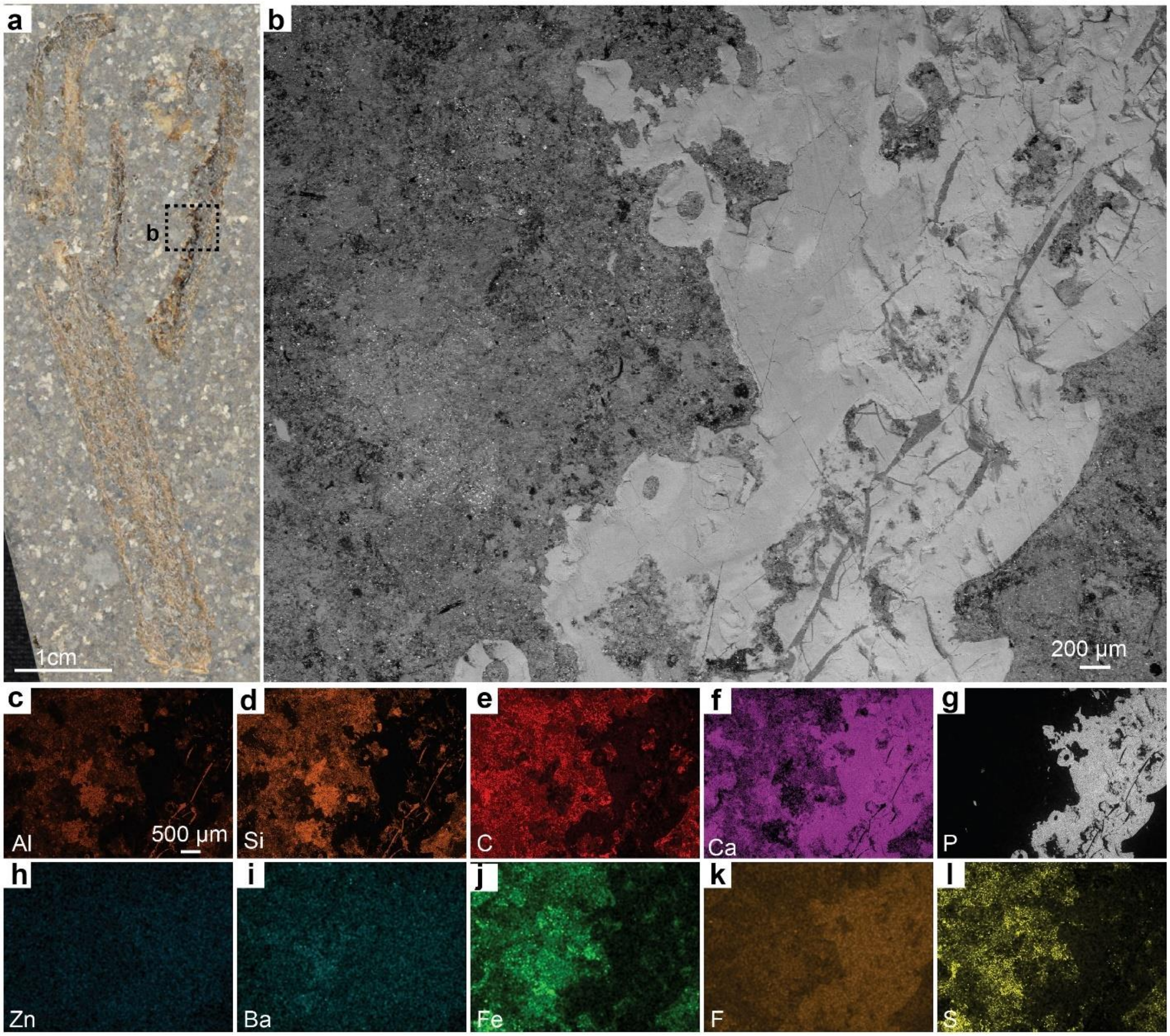

Supplementary Figure S47. Claw of *Uncina posidoniae* (NPL00036039.000) from the Posidonia Shale Lagerstätte (Fleins layer; Germany) prepared as a polished slab. **a**, Reflected-light photograph of specimen. **b**, Magnified BSE-SEM image of the box in **a**. **c-l**, EDS elemental maps of **b** showing phosphatized carapace.

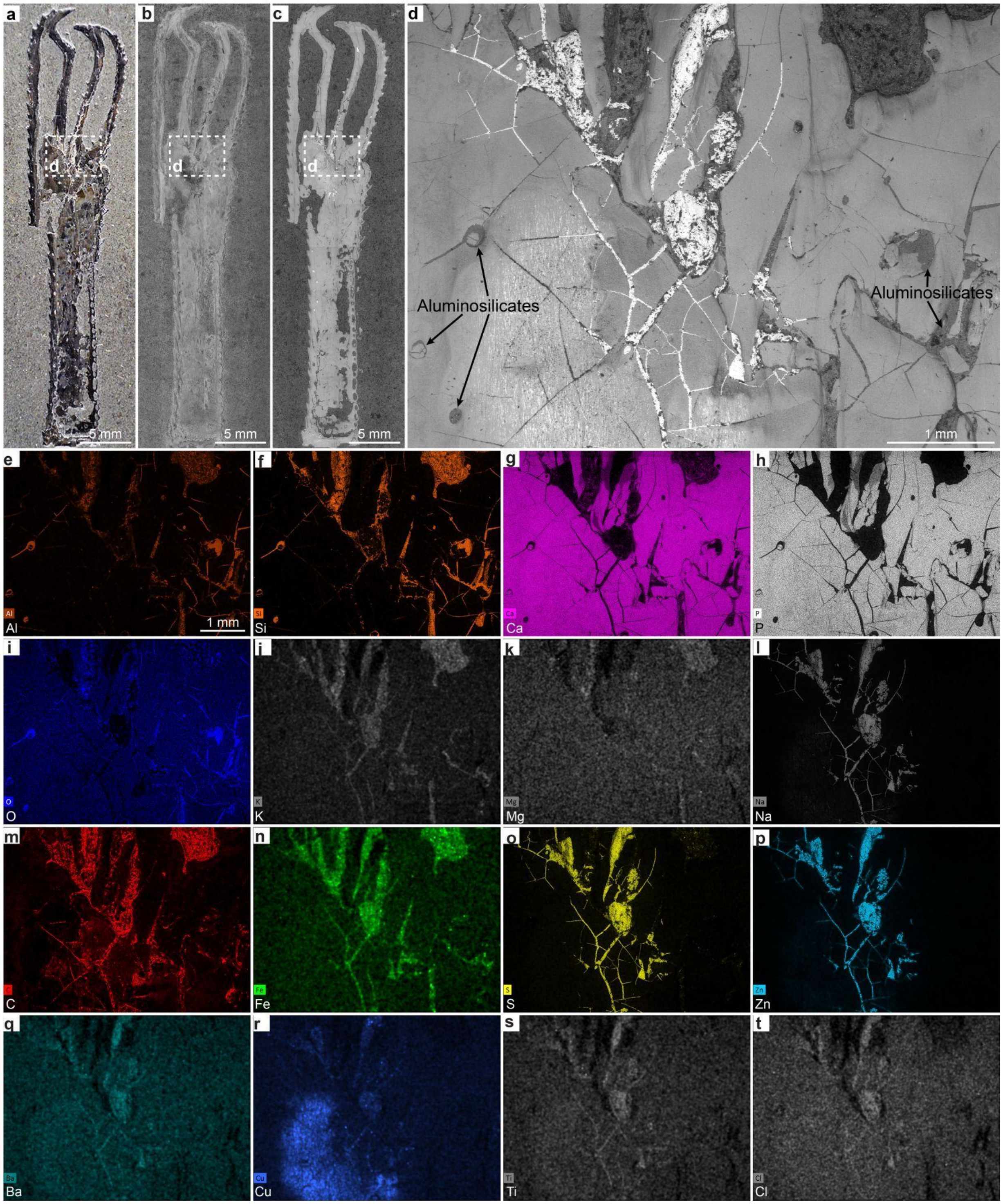

Supplementary Figure S48. Claw of *Uncina posidoniae* (NPL00036038.000) from the Posidonia Shale Lagerstätte (Holzmaden, Germany). **a**, Reflected-light photograph of the specimen. **b**, Mosaic SE-SEM image of the specimen. **a**, Mosaic BSE-SEM image of specimen. **d**, Magnified BSE-SEM image of boxes in **a-c**, showing crack-filling and void-filling sulfide (i.e., pyrite and sphalerite) and aluminosilicate minerals. **e-t**, EDS elemental maps of **d**.

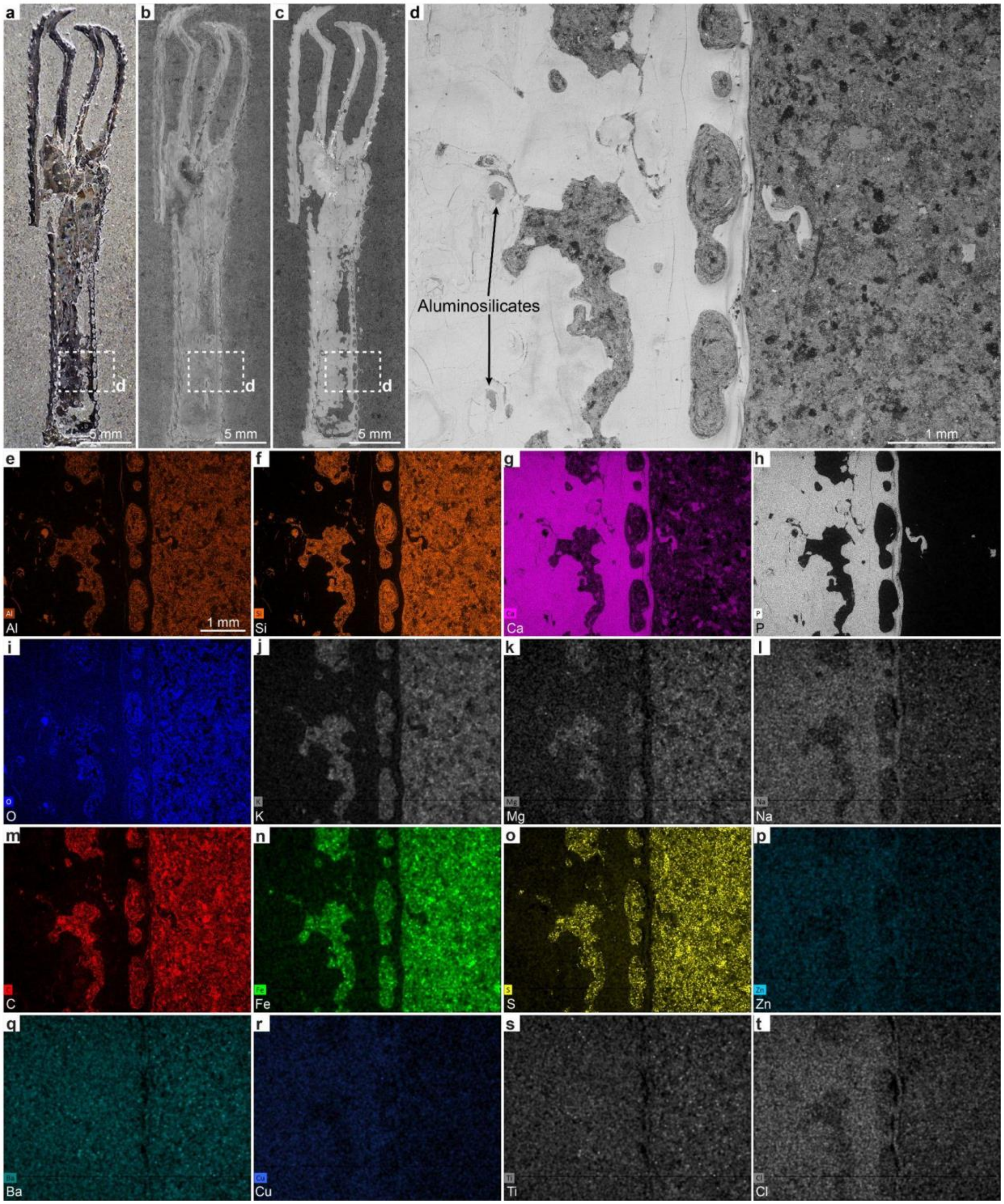

Supplementary Figure S49. Claw of *Uncina posidoniae* (NPL00036038.000) from the Posidonia Shale Lagerstätte (Holzmaden, Germany) with voids filled with aluminosilicate minerals and shale matrix. **a**, Reflected-light photograph of the specimen. **b**, Mosaic SE-SEM image of the specimen. **c**, Mosaic BSE-SEM image of the specimen. **d**, Magnified BSE-SEM image of boxes in **a-c**, showing void-filling aluminosilicate minerals and areas of carapace filled by shale matrix. **e-t**, EDS elemental maps of **d**.

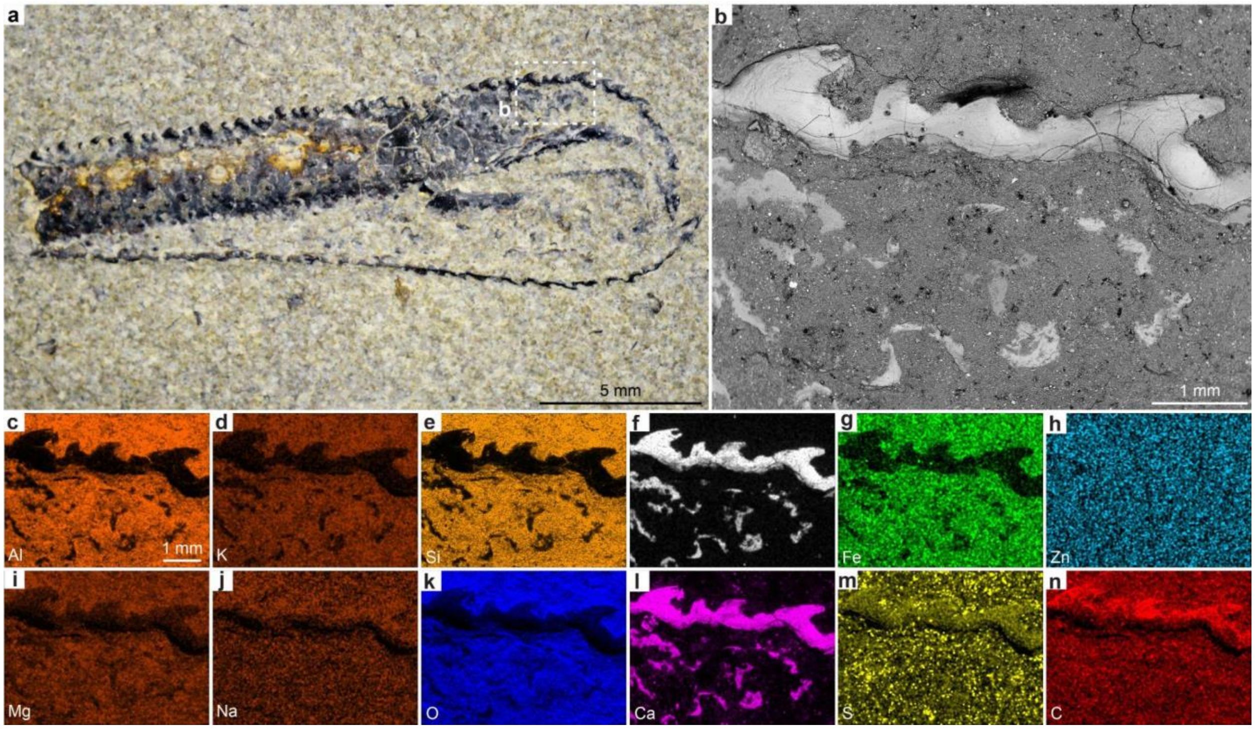

Supplementary Figure S50. Claw of *Uncina posidoniae* (NPL00094459.000) from the Posidonia Shale Lagerstätte (Unterer Schiefer layer; Dormettingen near Dotternhausen, Germany). **a**, Reflected-light photograph of the specimen. **b**, Magnified BSE-SEM image of box in **a**, showing phosphatized carapace in a shale matrix. **c-n**, EDS elemental maps of **b**.

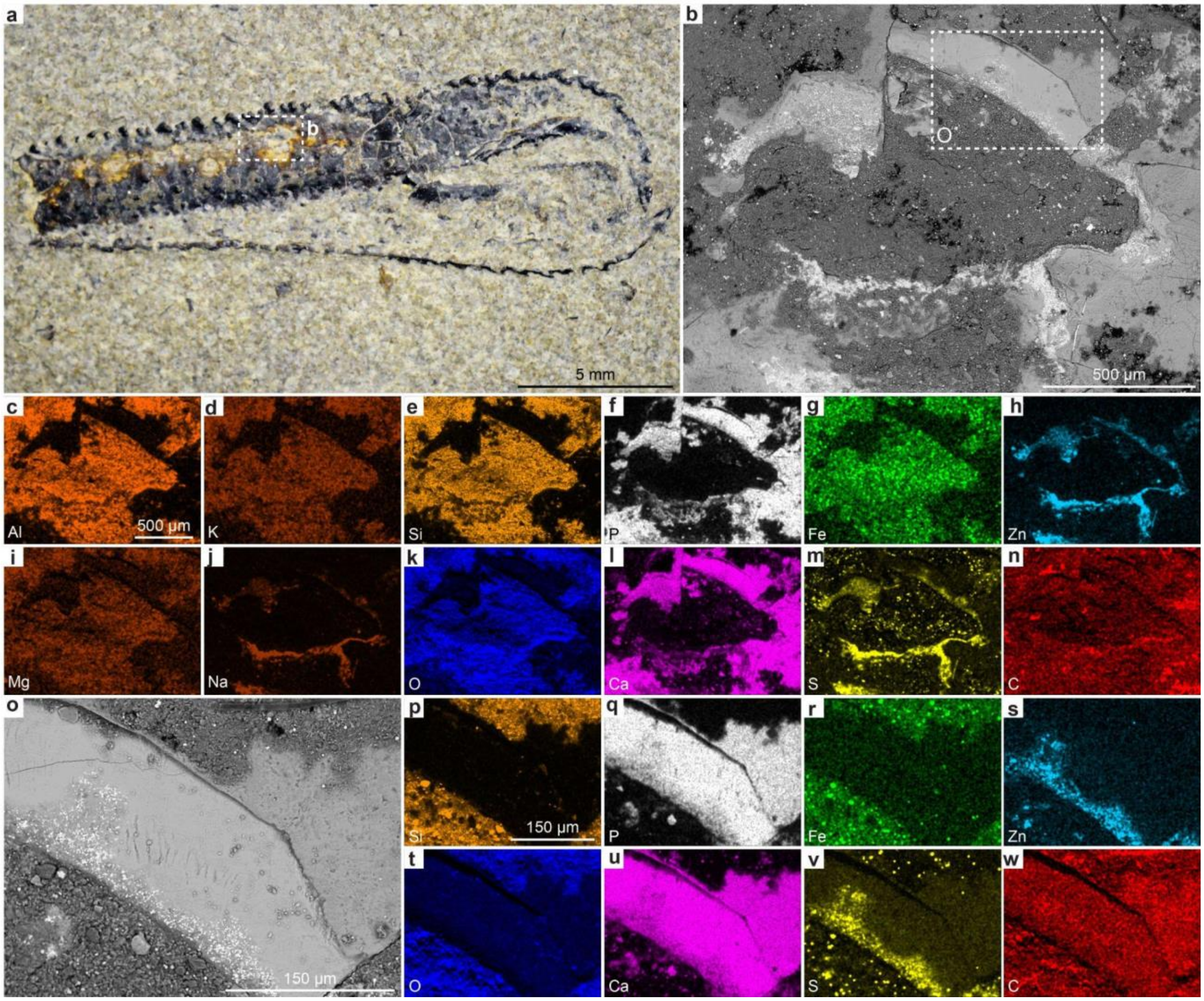

Supplementary Figure S51. Claw of *Uncina posidoniae* (NPL00094459.000) from the Posidonia Shale Lagerstätte (Unterer Schiefer layer; Dormettingen near Dotternhausen, Germany) with sulfide minerals within phosphatized carapace. **a**, Reflected-light photograph of specimen. **b**, Magnified BSE-SEM image of box in **a**, showing phosphatized carapace in shale matrix. **c-n**, EDS elemental maps of **b**. **o**, Magnified BSE-SEM image of box in **b**, showing sulfide minerals (i.e., sphalerite and pyrite) within the phosphatic carapace. **p-w**, EDS elemental maps of **o**.

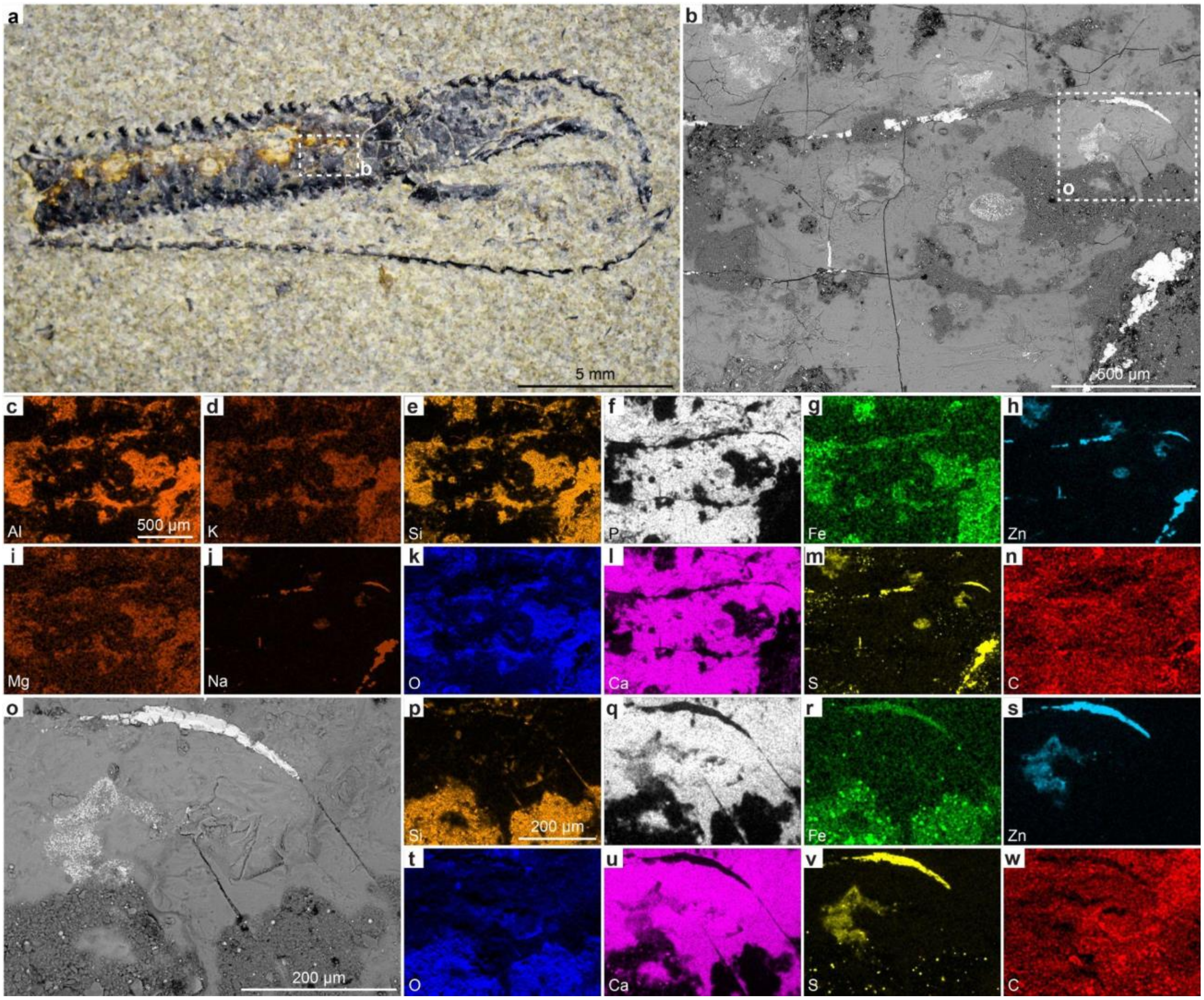

Supplementary Figure S52. Claw of *Uncina posidoniae* (NPL00094459.000) from the Posidonia Shale Lagerstätte (Unterer Schiefer layer; Dormettingen near Dotternhausen, Germany) with crack-filling sulfide minerals. **a**, Reflected-light photograph of the specimen. **b**, Magnified BSE-SEM image of box in **a**, showing phosphatized carapace in shale matrix. **c-n**, EDS elemental maps of **b**. **o**, Magnified BSE-SEM image of box in **b**, showing cracking-filling sulfide minerals (i.e., sphalerite and pyrite) as well as sulfide minerals within the phosphatic carapace. **p-w**, EDS elemental maps of **o**.

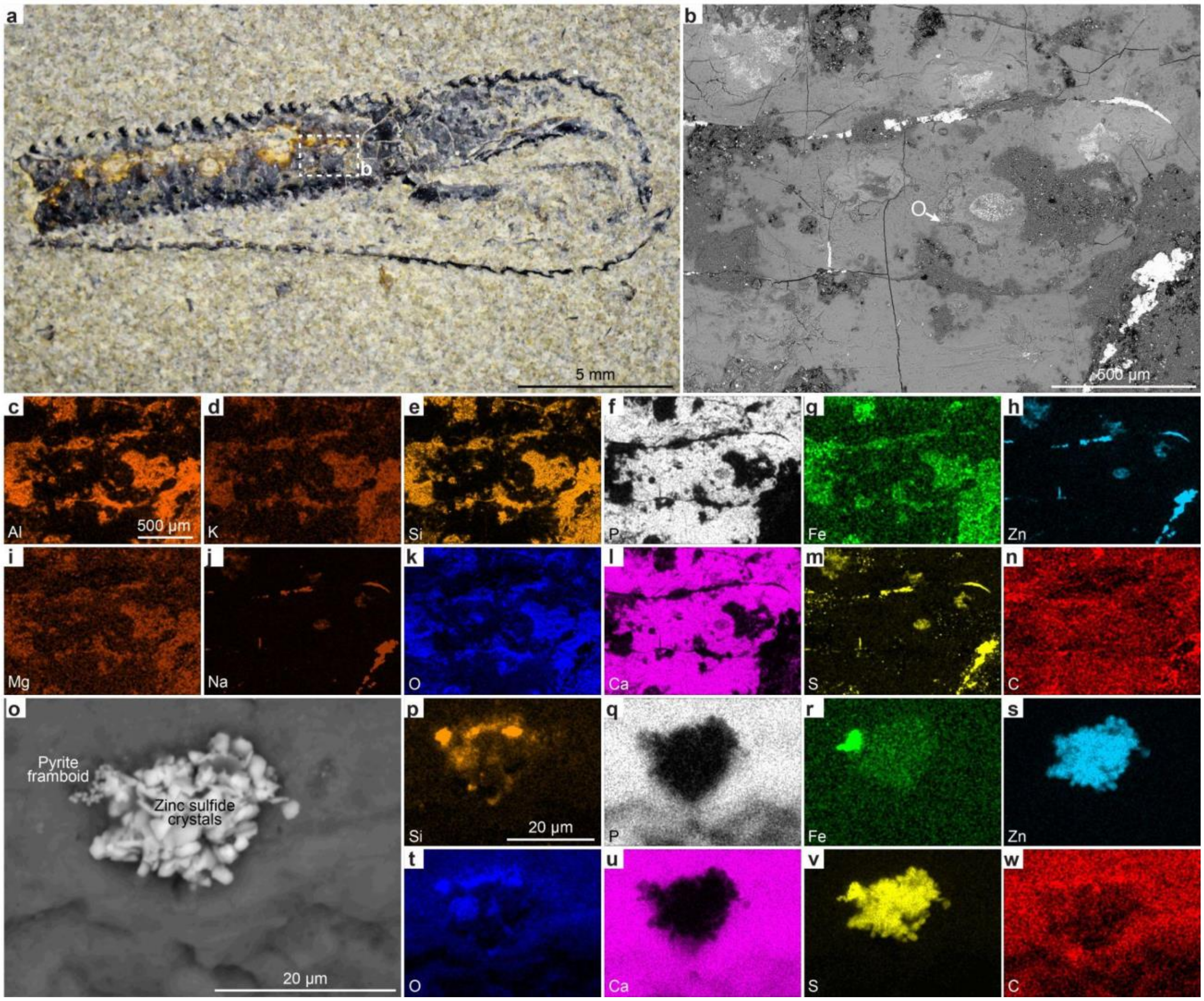

Supplementary Figure S53. Claw of *Uncina posidoniae* (NPL00094459.000) from the Posidonia Shale Lagerstätte (Unterer Schiefer layer; Dormettingen near Dotternhausen, Germany) with pyrite framboids and euhedral/subhedral crystals of sphalerite (zinc sulfide). **a**, Reflected-light photograph of the specimen. **b**, Magnified BSE-SEM image of box in **a**, showing phosphatized carapace in shale matrix. **c-n**, EDS elemental maps of **b**. **o**, Magnified BSE-SEM image of point identified in **b**, showing pyrite framboid and euhedral/subhedral crystals of sphalerite on the phosphatic carapace. **p-w**, EDS elemental maps of **o**.

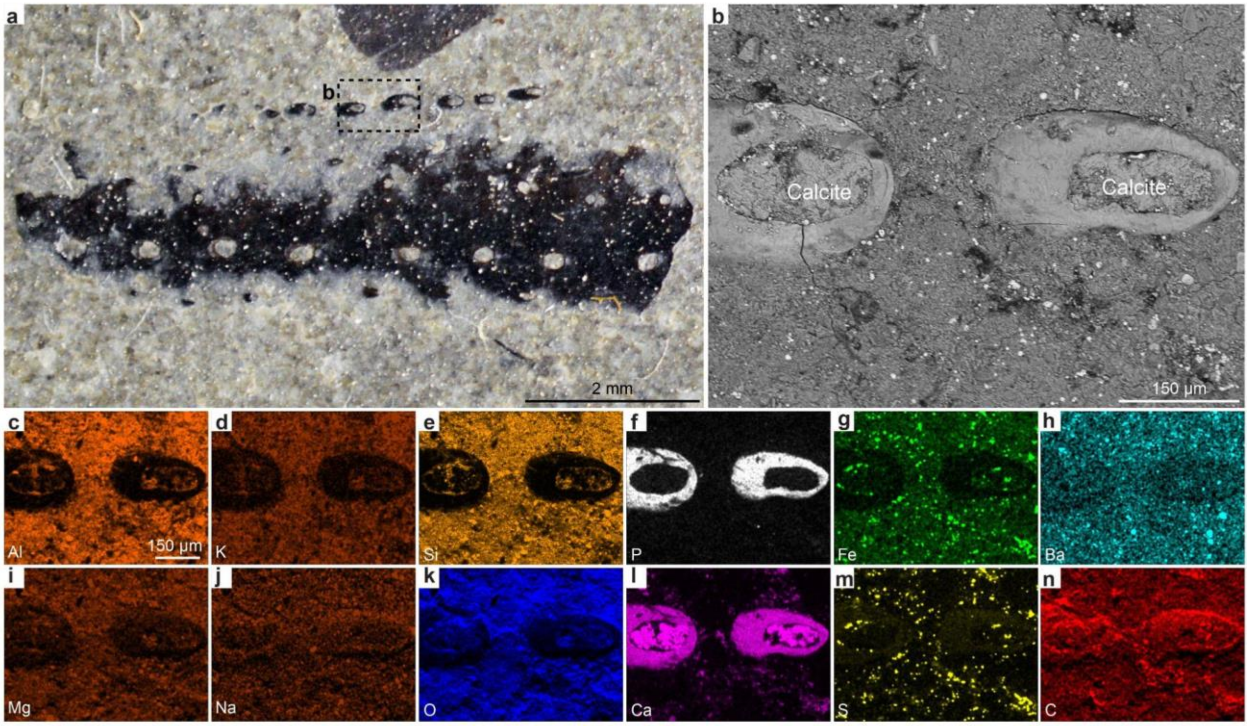

Supplementary Figure S54. Claw of *Uncina posidoniae* (NPL00094458.000) from the Posidonia Shale Lagerstätte (Unterer Schiefer layer; Dormettingen near Dotternhausen, Germany) filled with blocky calcite. **a**, Reflected-light photograph of the specimen. **b**, Magnified BSE-SEM image of box in **a**, showing phosphatized carapace, filled with blocky calcite, in shale matrix. **c-n**, EDS elemental maps of **b**.

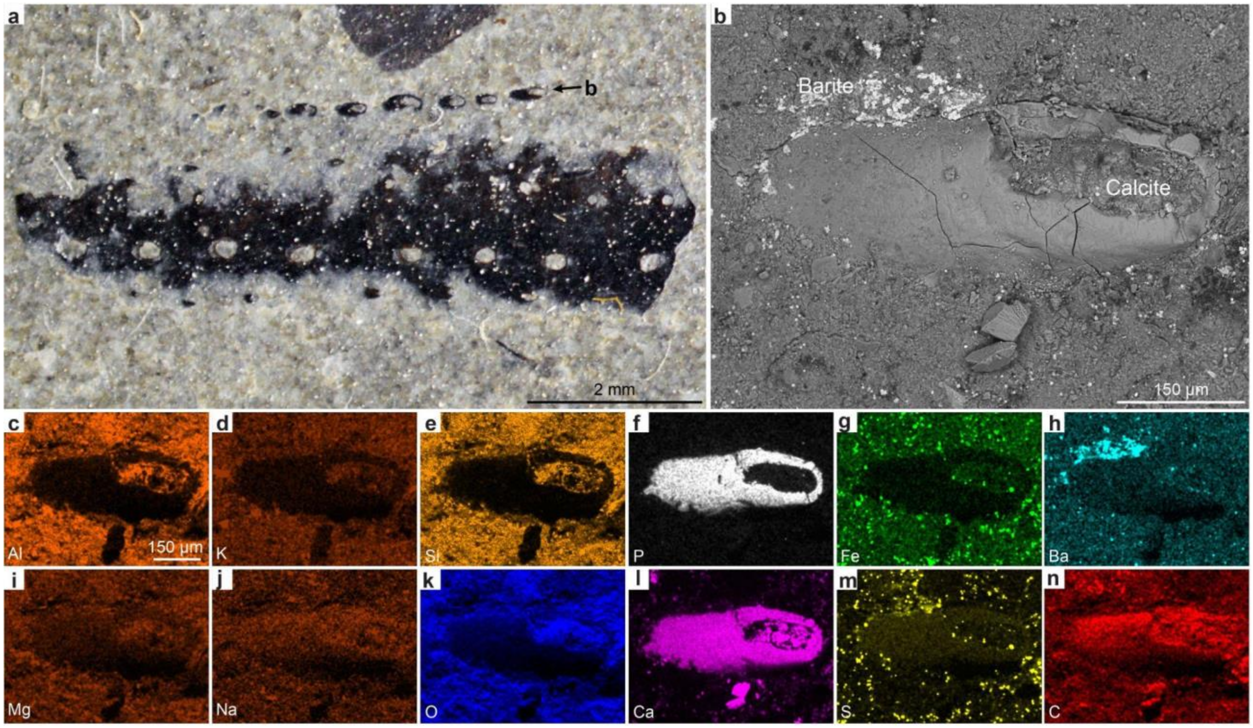

Supplementary Figure S55. Claw of *Uncina posidoniae* (NPL00094458.000) from the Posidonia Shale (Unterer Schiefer layer; Dormettingen near Dotternhausen, Germany) filled with blocky calcite and encrusted by barite. **a**, Reflected-light photograph of specimen. **b**, Magnified BSE-SEM image of box in **a**, showing phosphatized carapace, filled with blocky calcite and encrusted by barite, in shale matrix. **c-n**, EDS elemental maps of **b**.

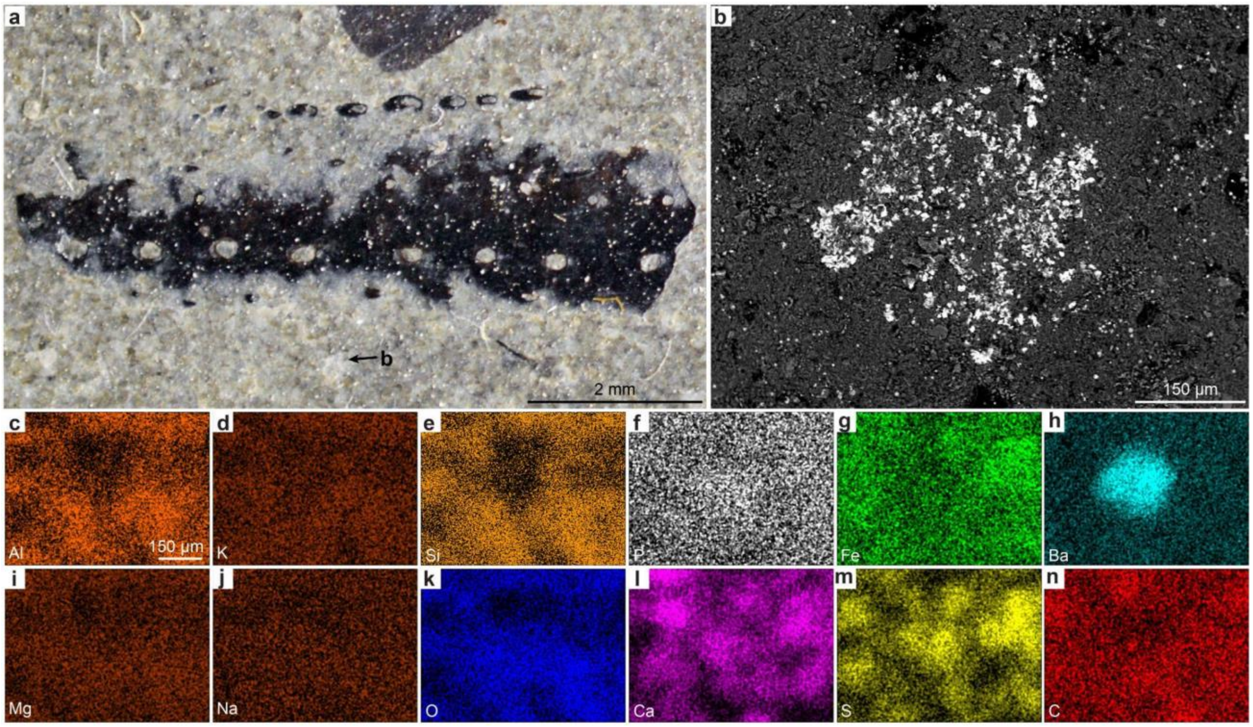

Supplementary Figure S56. Claw of *Uncina posidoniae* (NPL00094458.000) from the Posidonia Shale Lagerstätte (Unterer Schiefer layer; Dormettingen near Dotternhausen, Germany) surrounded by shale matrix with barite. **a**, Reflected-light photograph of the specimen. **b**, Magnified BSE-SEM image of point identified in **a**, showing phosphatized carapace surrounded by shale matrix containing barite. **c-n**, EDS elemental maps of **b**.

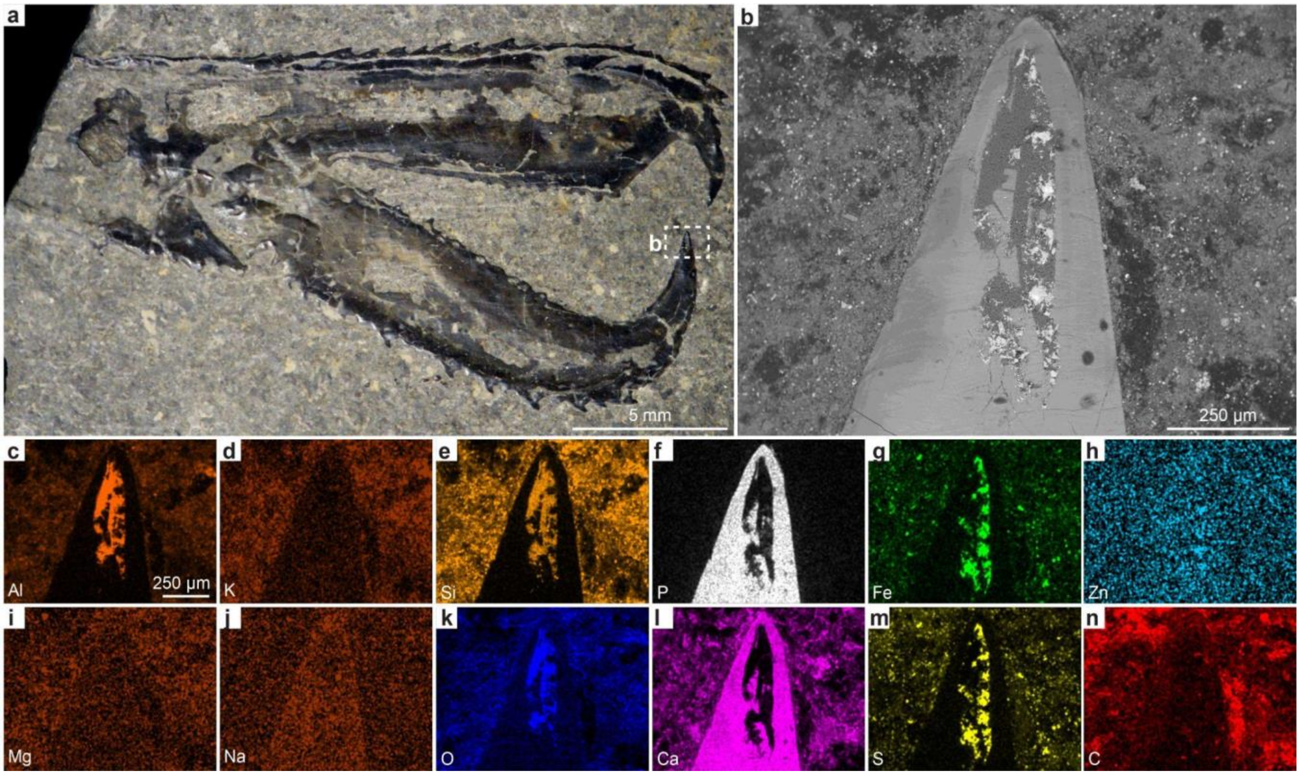

Supplementary Figure S57. Claw of *Uncina posidoniae* (NPL00094457.000) from the Posidonia Shale Lagerstätte (Ohmden, Germany) with void-filling minerals. **a**, Reflected-light photograph of the specimen. **b**, Magnified BSE-SEM image of box in **a**, showing phosphatized carapace, filled by sulfide (e.g. pyrite and sphalerite) and aluminosilicate minerals, surrounded by shale matrix. **c**-**n**, EDS elemental maps of **b**.

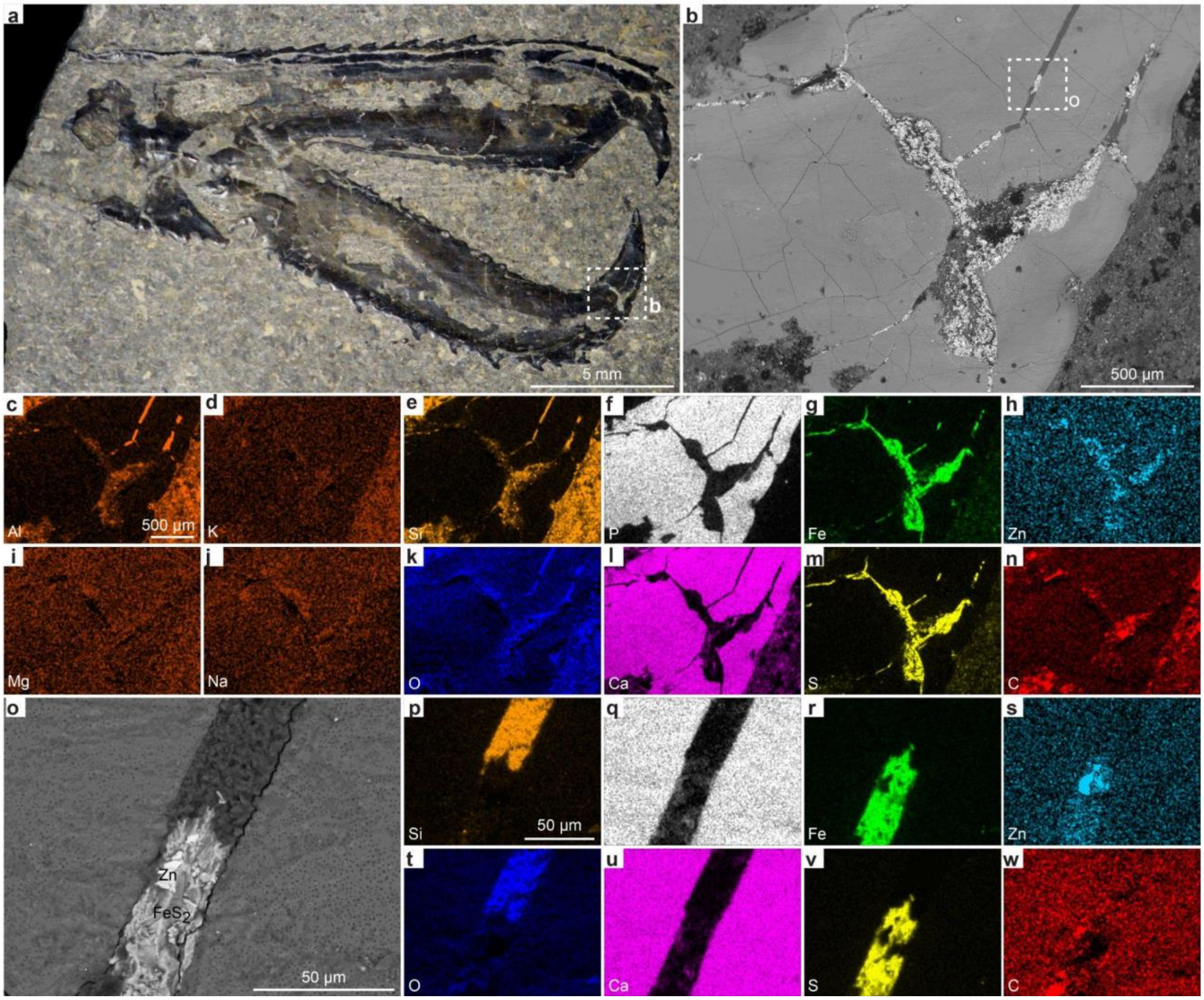

Supplementary Figure S58. Claw of *Uncina posidoniae* (NPL00094457.000) from the Posidonia Shale Lagerstätte (Ohmden, Germany) with crack- and void-filling minerals. **a**, Reflected-light photograph of the specimen. **b**, Magnified BSE-SEM image of box in **a**, showing phosphatized carapace, containing void- and crack-filling sulfide (e.g., pyrite and sphalerite) and aluminosilicate minerals, surrounded by shale matrix. **c-n**, EDS elemental maps of **b**. **o**, Magnified BSE-SEM image of box in **b**, showing crack in phosphatized carapace filled with zinc sulfide, iron sulfide, and aluminosilicate minerals. **p-w**, EDS elemental maps of **o**.

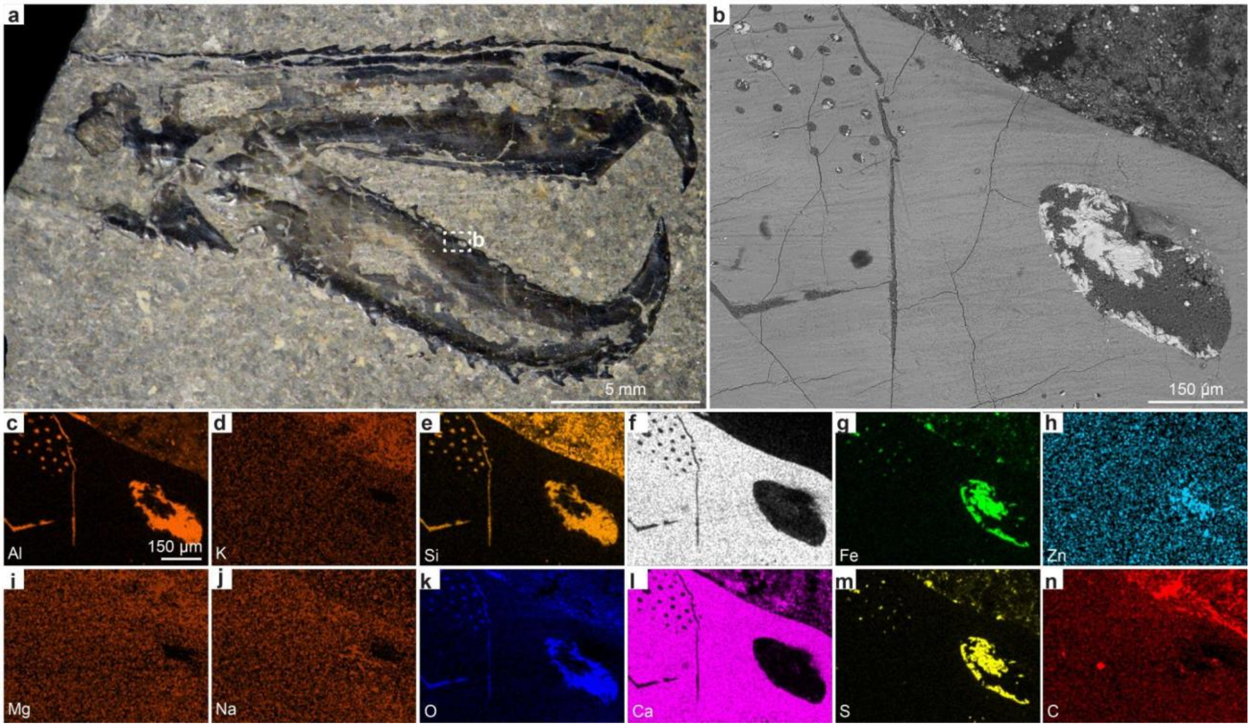

Supplementary Figure S59. Claw of *Uncina posidoniae* (NPL00094457.000) from the Posidonia Shale Lagerstätte (Ohmden, Germany) with ultrastructural and morphological features filled with minerals. **a**, Reflected-light photograph of the specimen. **b**, Magnified BSE-SEM image of box in **a**, showing phosphatized carapace, filled by sulfide (e.g., pyrite and sphalerite) and aluminosilicate minerals, surrounded by shale matrix. **c-n**, EDS elemental maps of **b**.

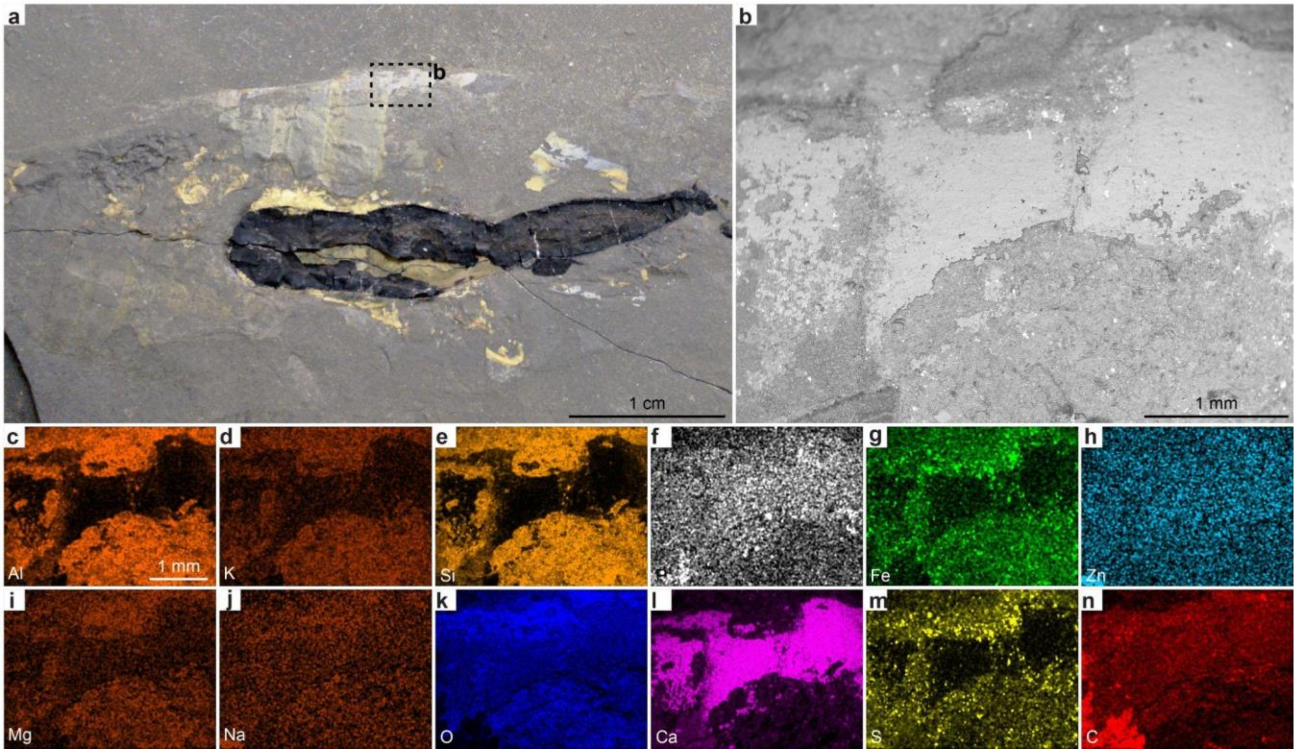

Supplementary Figure S60. Vampyropod *Clarkeiteuthis* sp. (NPL00094460.000) gladius with ink sac from the Posidonia Shale Lagerstätte (Koblenzer or Hainzen layer at Holzmaden, Germany). **a**, Reflected-light photograph of the specimen. **b**, Magnified BSE-SEM image of box in **a**, showing calcified gladius material. **c-n**, EDS elemental maps of **b**.

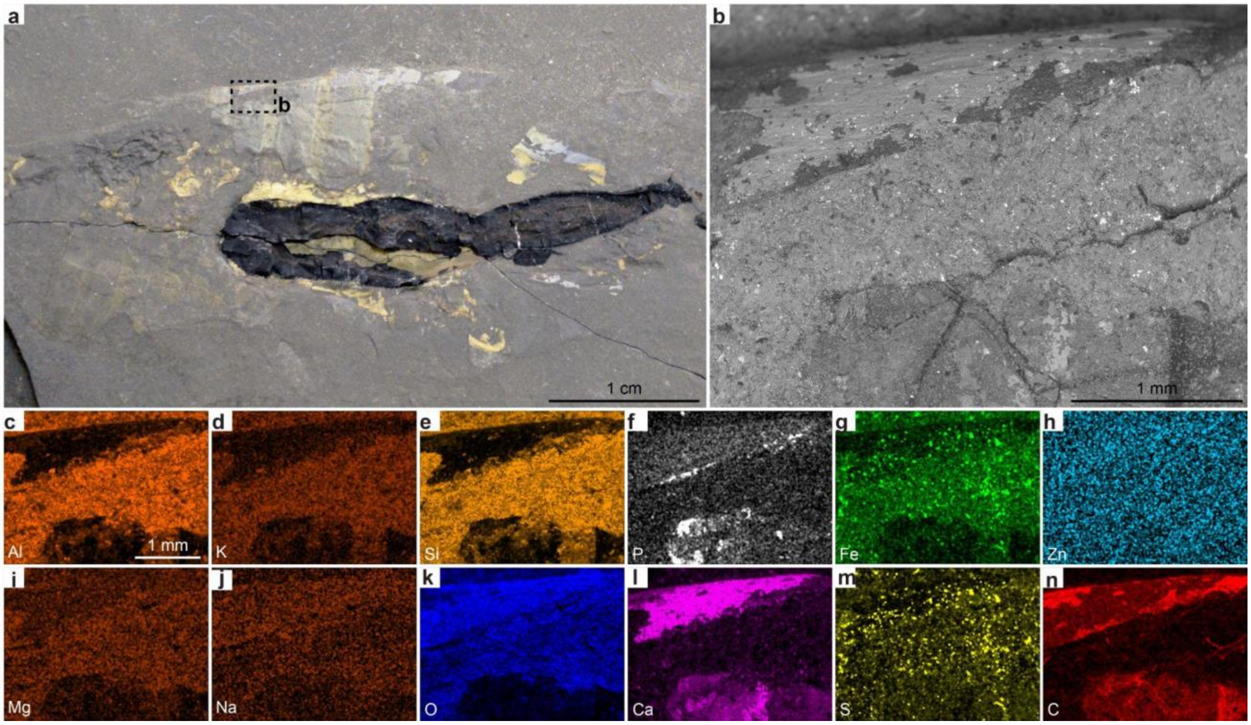

Supplementary Figure S61. Vampyropod *Clarkeiteuthis* sp. (NPL00094460.000) gladius with ink sac from the Posidonia Shale Lagerstätte (Koblenzer or Hainzen layer at Holzmaden, Germany). **a**, Reflected-light photograph of the specimen. **b**, Magnified BSE-SEM image of box in **a**, showing calcified gladius material associated with carbonaceous material. **c-n**, EDS elemental maps of **b**.

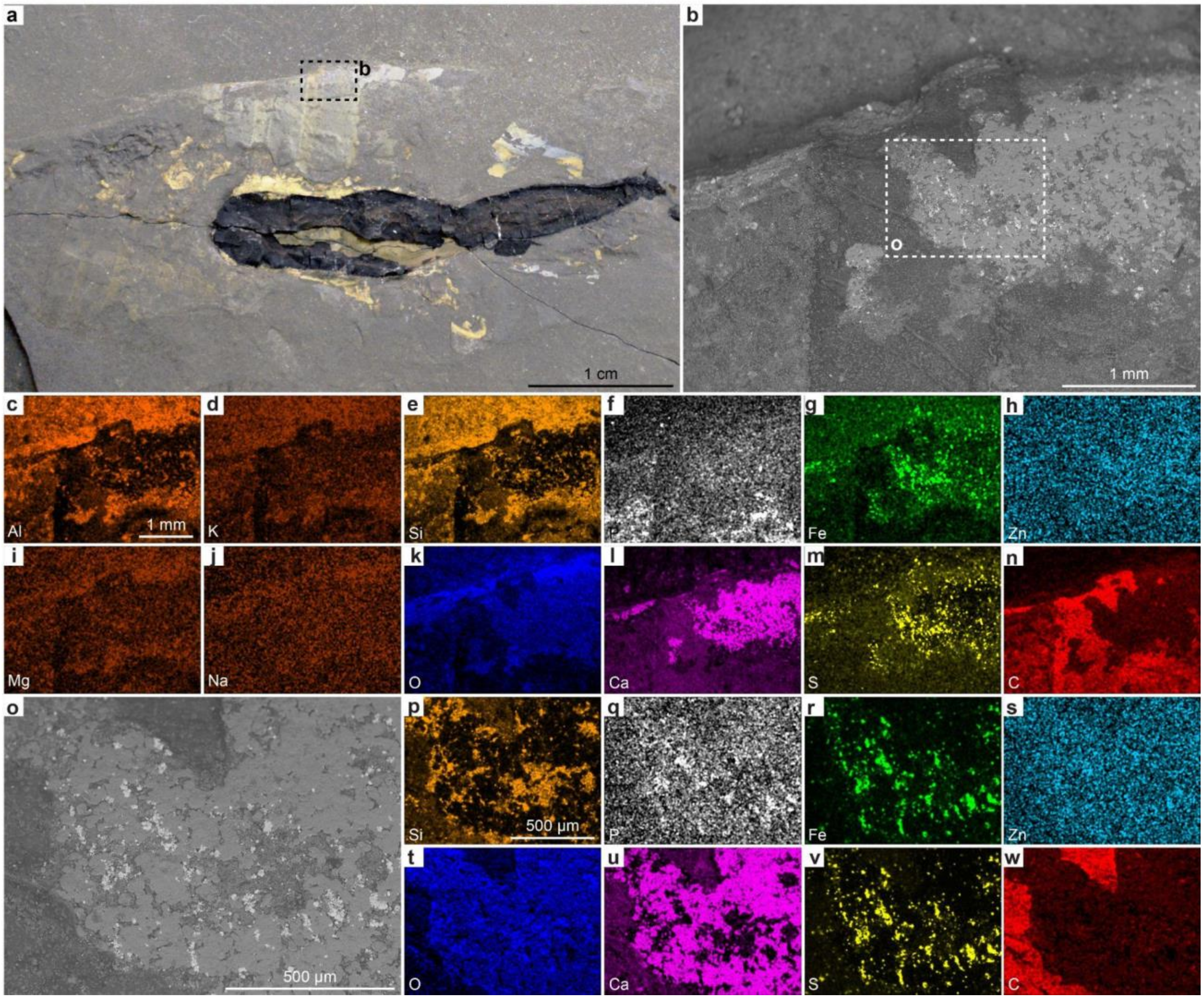

Supplementary Figure S62. Vampyropod *Clarkeiteuthis* sp. (NPL00094460.000) gladius with ink sac from the Posidonia Shale Lagerstätte (Koblenzer or Hainzen layer at Holzmaden, Germany). **a**, Reflected-light photograph of the specimen. **b**, Magnified BSE-SEM image of box in **a**, showing calcified gladius material. **c-n**, EDS elemental maps of **b**. **o**, Magnified BSE-SEM image of box in **b**, showing calcified gladius material containing sulfide minerals (i.e., euhedral/subhedral pyrite and sphalerite). **p-w**, EDS elemental maps of **o**.

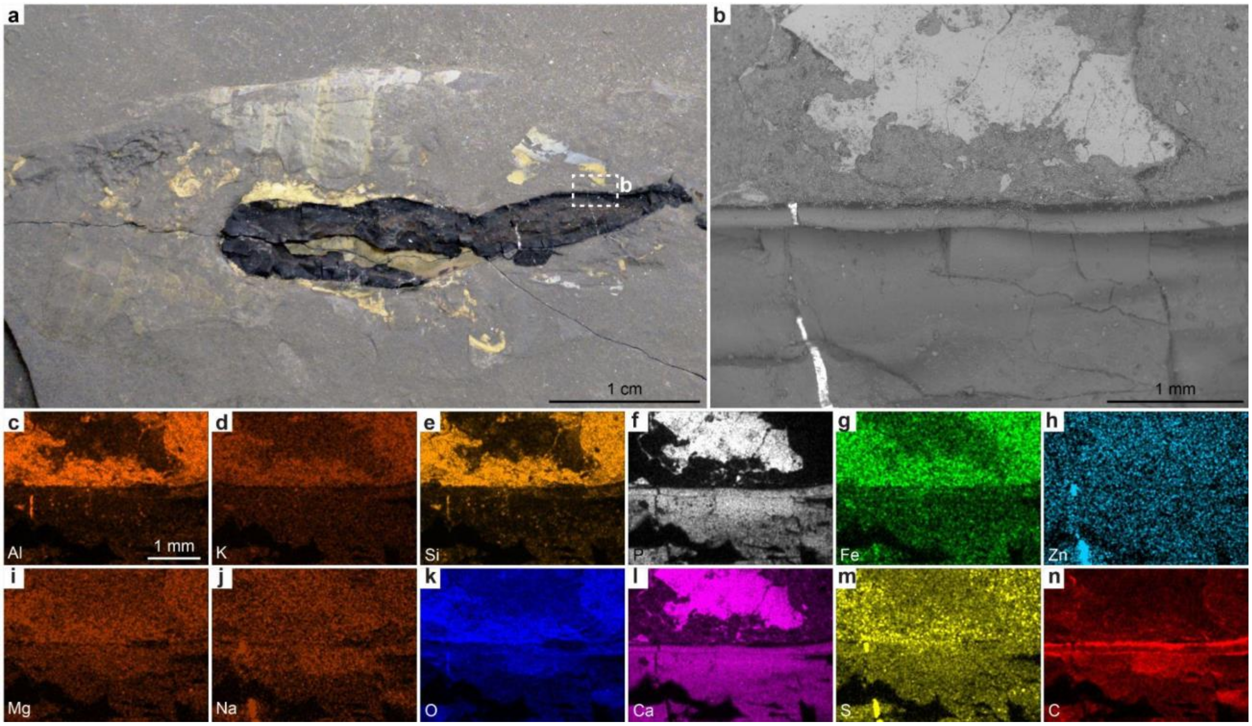

Supplementary Figure S63. Vampyropod *Clarkeiteuthis* sp. (NPL00094460.000) gladius with ink sac from the Posidonia Shale Lagerstätte (Koblenzer or Hainzen layer at Holzmaden, Germany). **a**, Reflected-light photograph of the specimen. **b**, Magnified BSE-SEM image of box in **a**, showing phosphatized gladius and ink sac material. Phosphatized ink sac contains crack-filling sulfide minerals (i.e., pyrite and sphalerite) and void-filling carbonaceous material. **c-n**, EDS elemental maps of **b**.

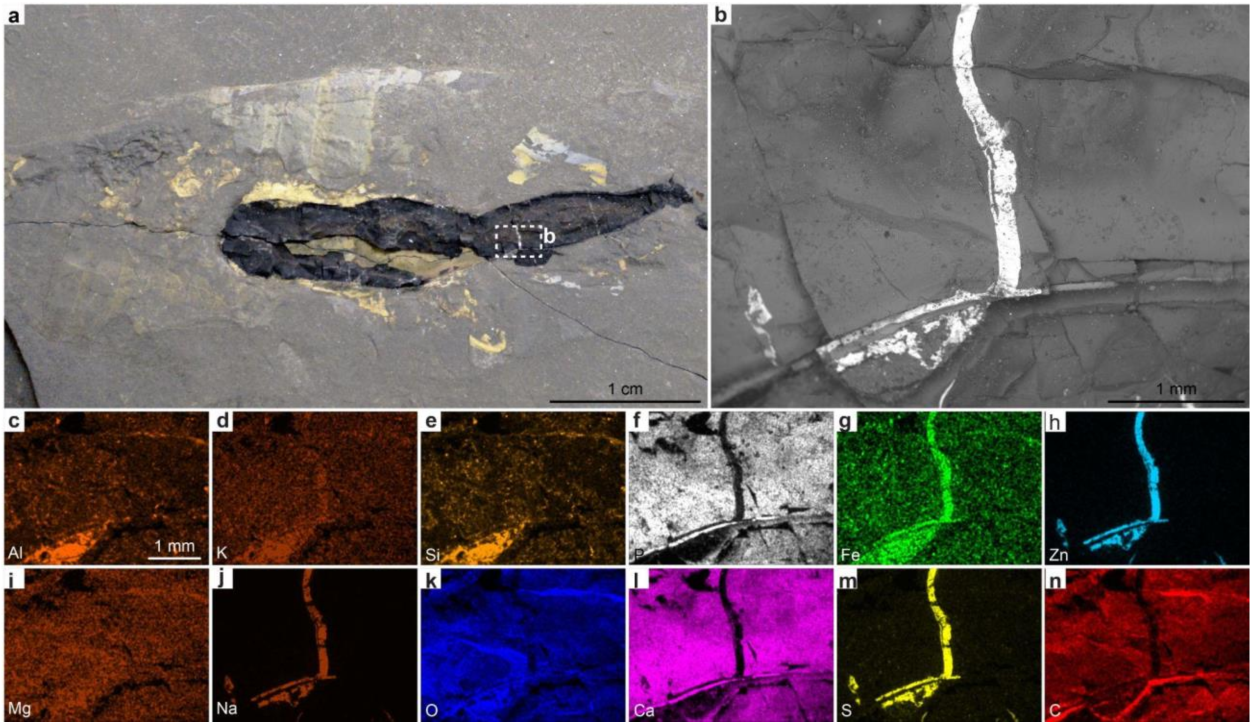

Supplementary Figure S64. Vampyropod *Clarkeiteuthis* sp. (NPL00094460.000) gladius with ink sac from the Posidonia Shale Lagerstätte (Koblenzer or Hainzen layer at Holzmaden, Germany). **a**, Reflected-light photograph of the specimen. **b**, Magnified BSE-SEM image of box in **a**, showing phosphatized ink sac containing crack-filling sulfide minerals (i.e. pyrite and sphalerite). **c-n**, EDS elemental maps of **b**.

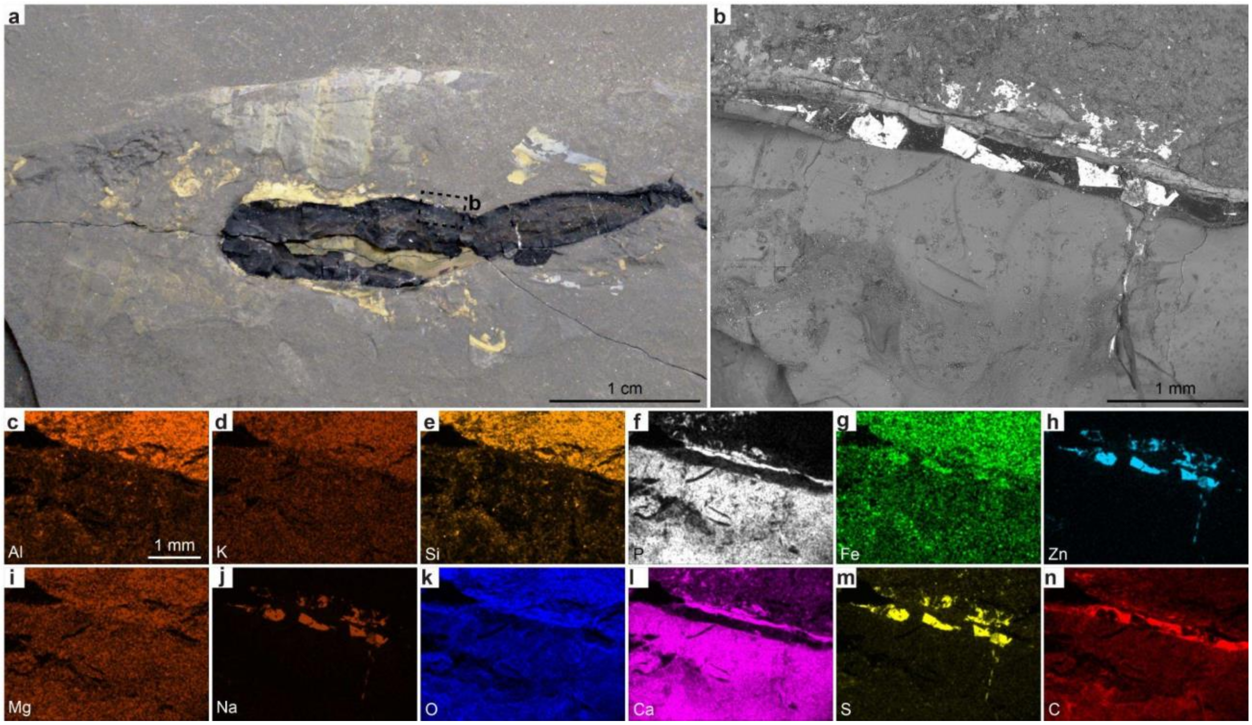

Supplementary Figure S65. Vampyropod *Clarkeiteuthis* sp. (NPL00094460.000) gladius with ink sac from the Posidonia Shale Lagerstätte (Koblenzer or Hainzen layer at Holzmaden, Germany). **a**, Reflected-light photograph of the specimen. **b**, Magnified BSE-SEM image of box in **a**, showing phosphatized ink sac, containing crack-filling sulfide minerals (i.e. pyrite and sphalerite) and void-filling carbonaceous material. **c-n**, EDS elemental maps of **b**.

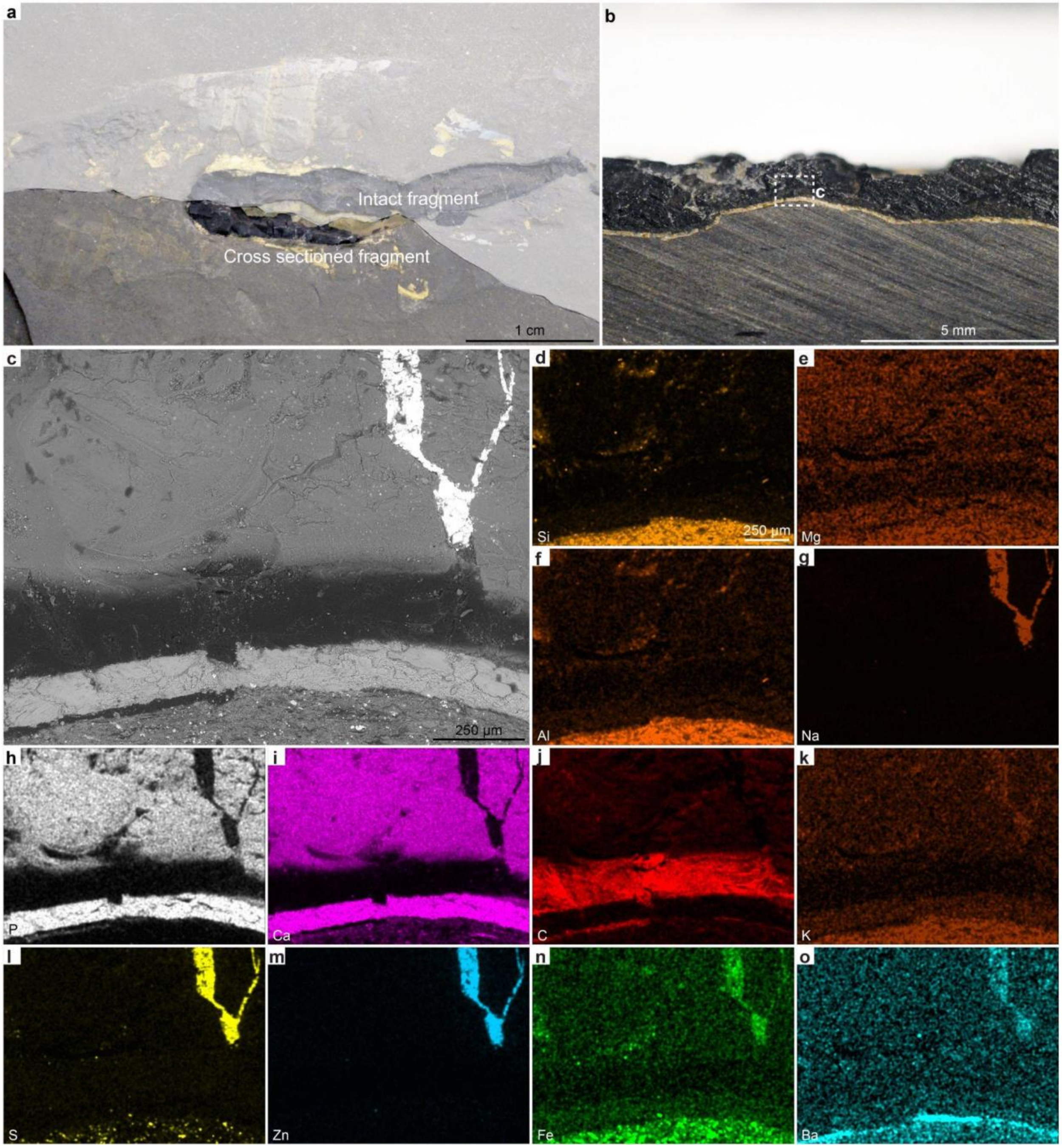

Supplementary Figure S66. Vampyropod *Clarkeiteuthis* sp. (NPL00094460.000) gladius with ink sac prepared as a polished transverse cross section from the Posidonia Shale Lagerstätte (Koblenzer or Hainzen layer at Holzmaden, Germany). **a**, Reflected-light photograph of an unprepared specimen. **b**, Reflected-light image of polished transverse cross section of ink sac. **c**, Magnified BSE-SEM image of box in **b**, showing phosphatized ink sac, containing crack-filling sulfide minerals (i.e., pyrite and sphalerite) and void-filling carbonaceous material. **d-o**, EDS elemental maps of **c**.

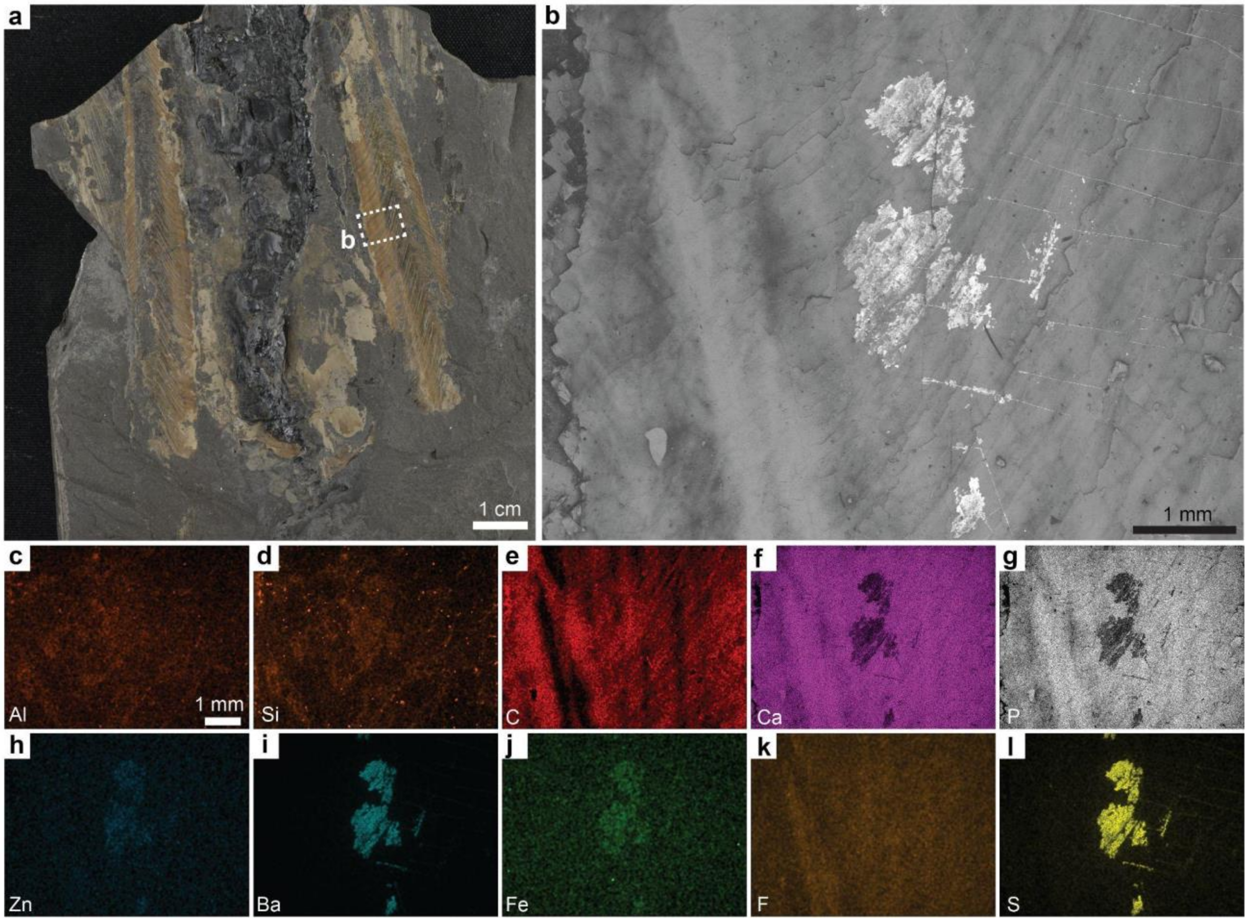

Supplementary Figure S67. Vampyropod gladius with ink sac of *Loligosepia aalensis* (NPL00036037.000), encrusted by barite from the Posidonia Shale Lagerstätte (Ohmden, Germany). **a**, Reflected-light photograph of the specimen. **b**, BSE-SEM image of the box in **a**. **c-l**, EDS elemental maps of **b** showing phosphatized gladius encrusted by barite, and minor pyrite and sphalerite.

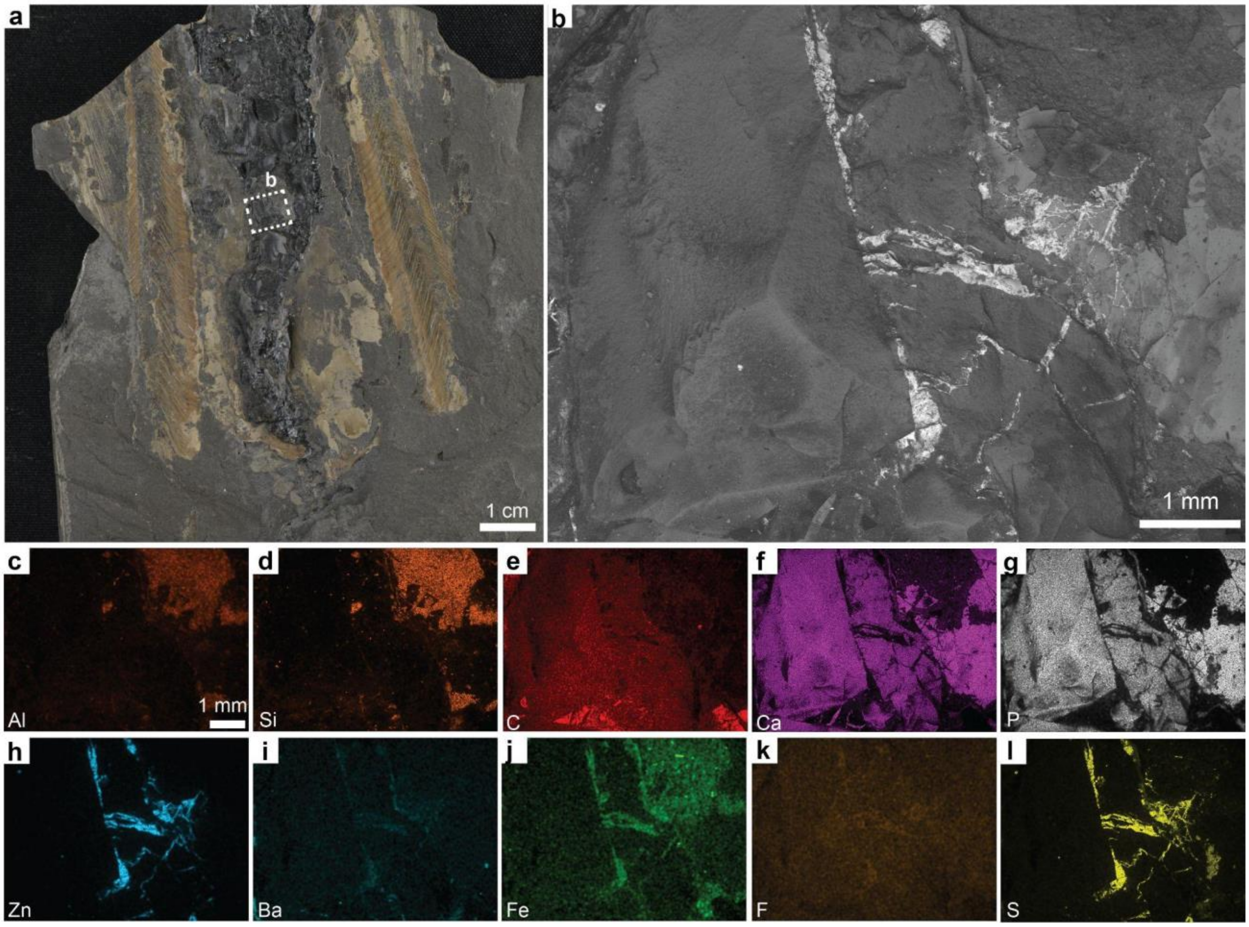

Supplementary Figure S68. Vampyropod gladius with ink sac of *Loligosepia aalensis* (NPL00036037.000), containing cracking-filling sulfide minerals from the Posidonia Shale Lagerstätte (Ohmden, Germany). **a**, Reflected-light photograph of the specimen. **b**, BSE-SEM image of the box in **a**. **c-l**, EDS elemental maps of **b** showing phosphatized ink sac containing crack-filling sulfide minerals (i.e., pyrite and sphalerite).

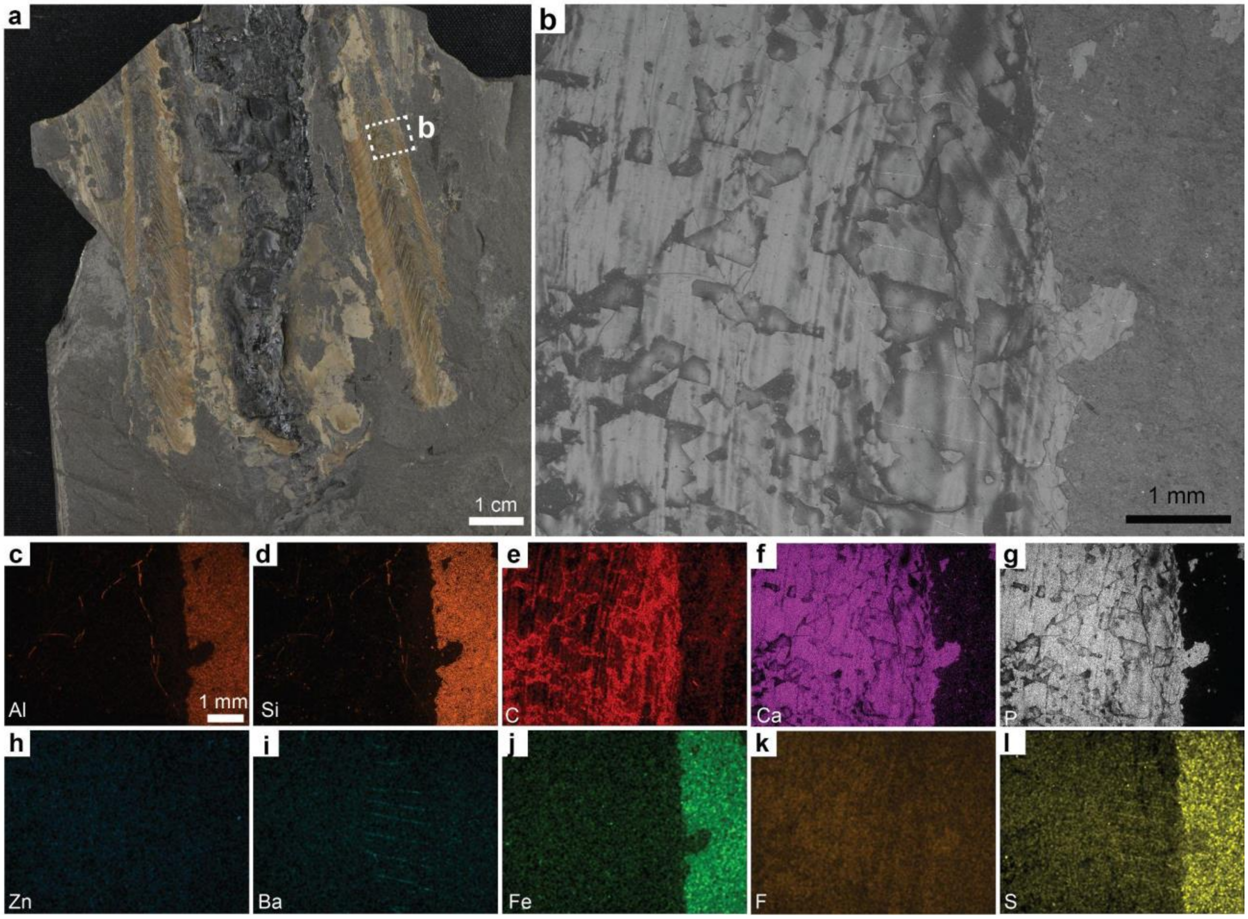

Supplementary Figure S69. Vampyropod *Loligosepia aalensis* gladius with ink sac (NPL00036037.000) from the Posidonia Shale Lagerstätte (Ohmden, Germany). **a**, Reflected-light photograph of the specimen. **b**, BSE-SEM images of the box in **a**. **c-l**, EDS elemental maps of **b** showing phosphatized gladius containing carbonaceous material.

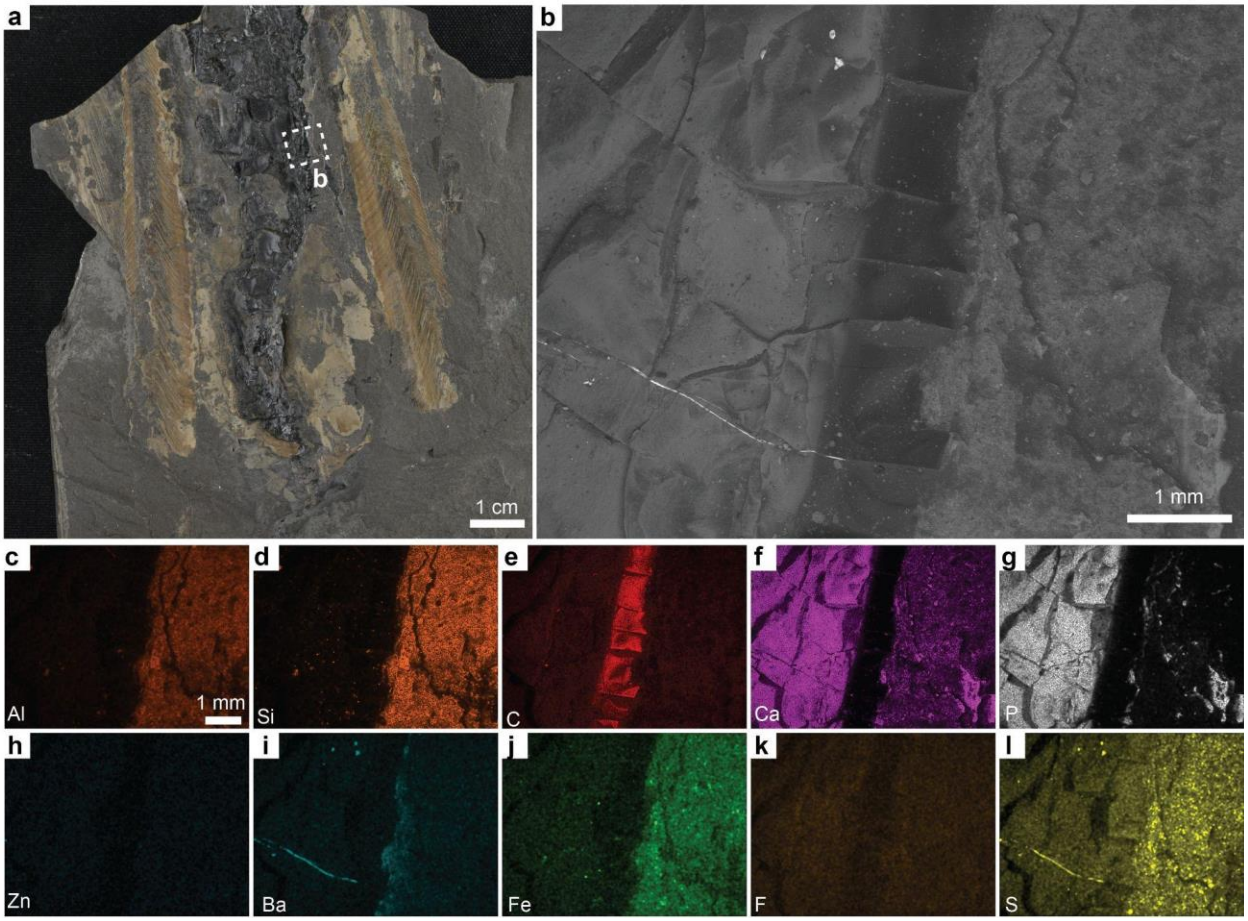

Supplementary Figure S70. Vampyropod *Loligosepia aalensis* gladius with ink sac (NPL00036037.000), surrounded by carbonaceous material from the Posidonia Shale Lagerstätte (Ohmden, Germany). **a**, Reflected-light photograph of the specimen. **b**, Magnified view of BSE-SEM image of the box in **a**. **c-l**, EDS elemental maps of **b** showing phosphatized ink sac surrounded by carbonaceous material.

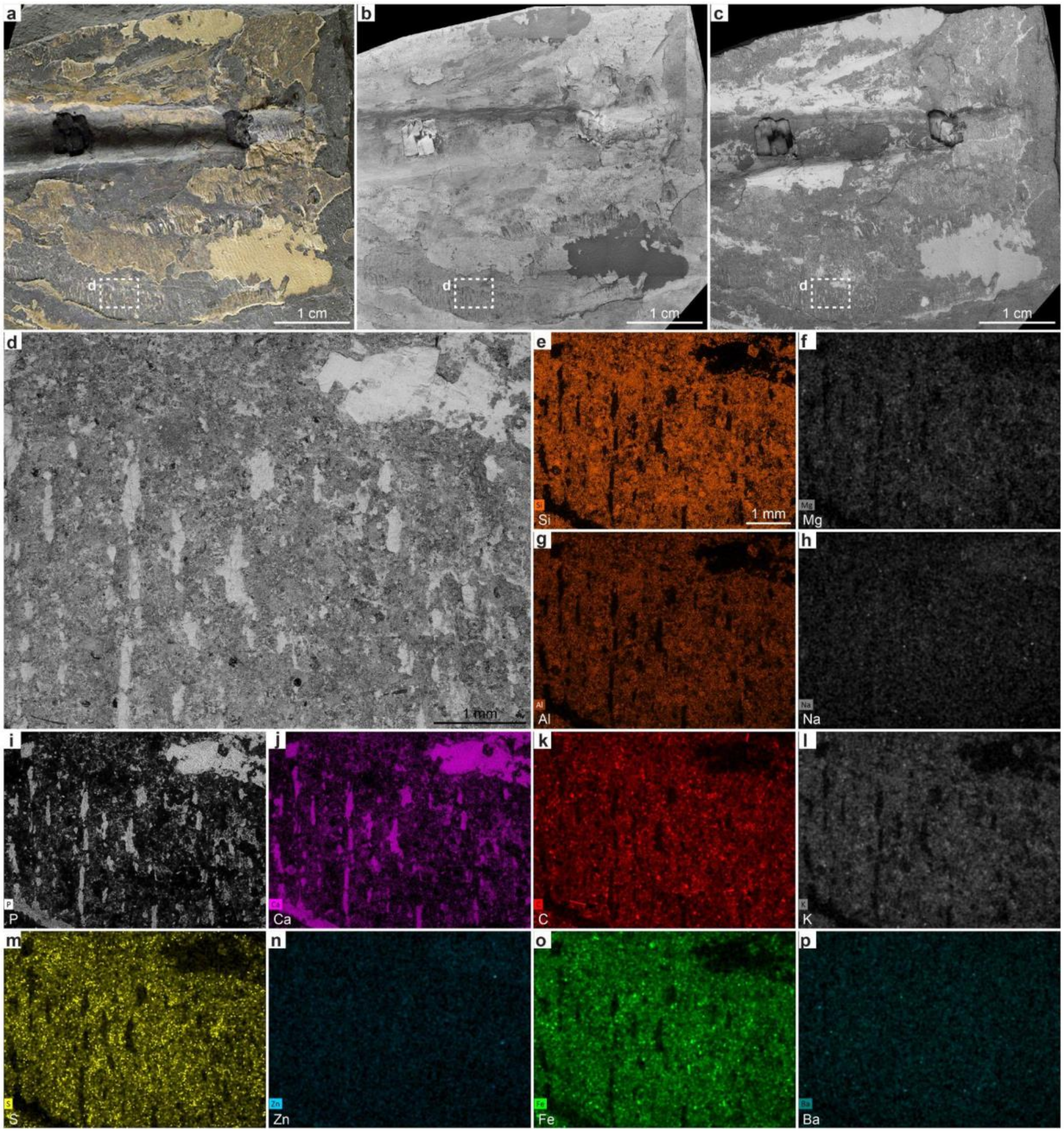

Supplementary Figure S71. Vampyropod *Loligosepia* sp. gladius (NPL00036035.000) with pieces of ink sac from the Posidonia Shale Lagerstätte (Holzmaden, Germany). **a**, Reflected-light photograph of specimen. **b**, Mosaic SE-SEM image of specimen. **c-d**, BSE-SEM images. **c**, Mosaic image of specimen. **d**, Magnified view of boxes in **a-c**, showing phosphatized material of gladius. **e-p**, EDS elemental maps of **d**.

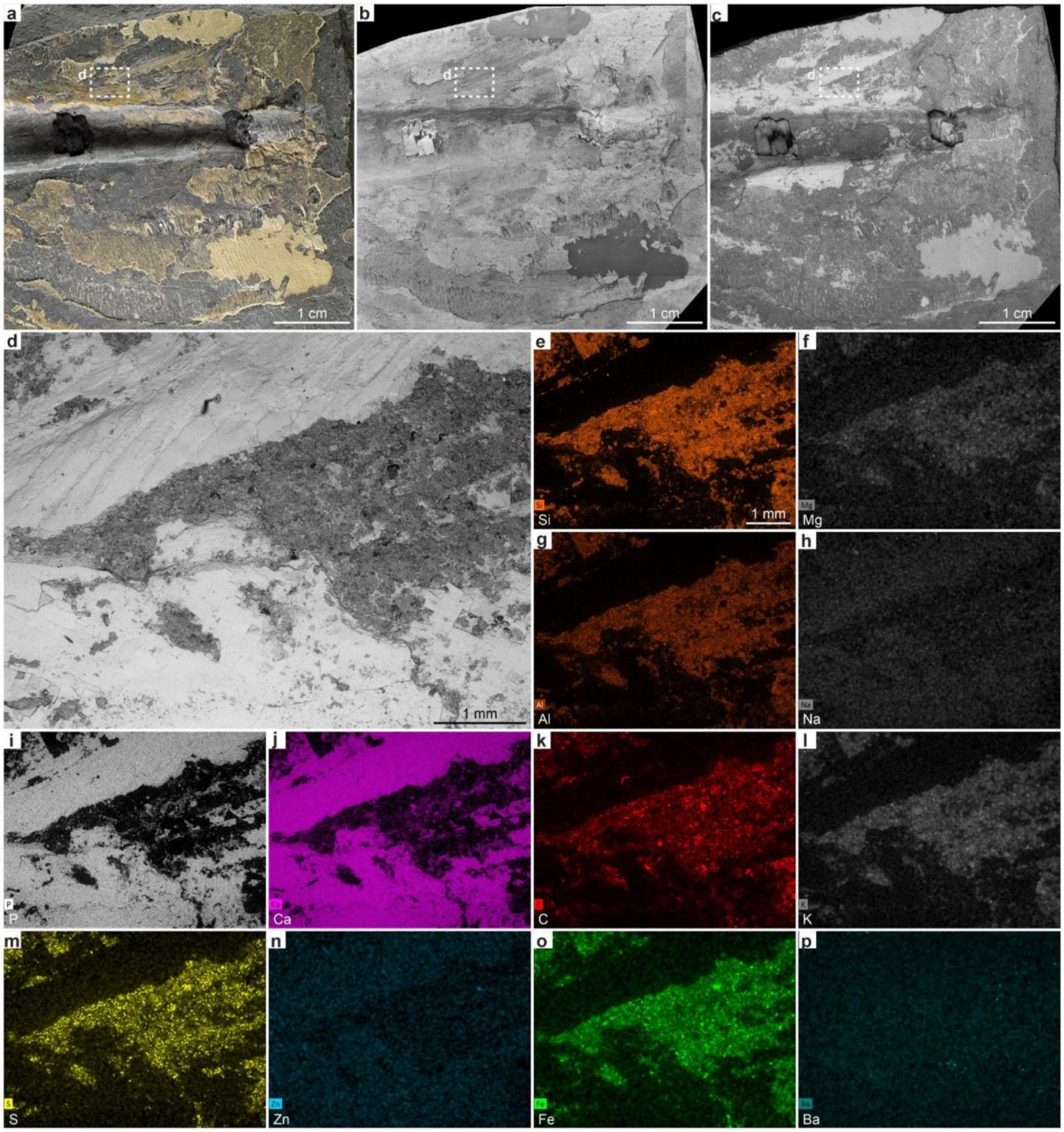

Supplementary Figure S72. Vampyropod *Loligosepia* sp. gladius (NPL00036035.000) with pieces of ink sac from the Posidonia Shale Lagerstätte (Holzmaden, Germany). **a**, Reflected-light photograph of the specimen. **b**, Mosaic SE-SEM image of the specimen. **c-d**, BSE-SEM images. **c**, Mosaic image of the specimen. **d**, Magnified view of boxes in **a-c**, showing phosphatized material of the gladius. **e-p**, EDS elemental maps of **d**.

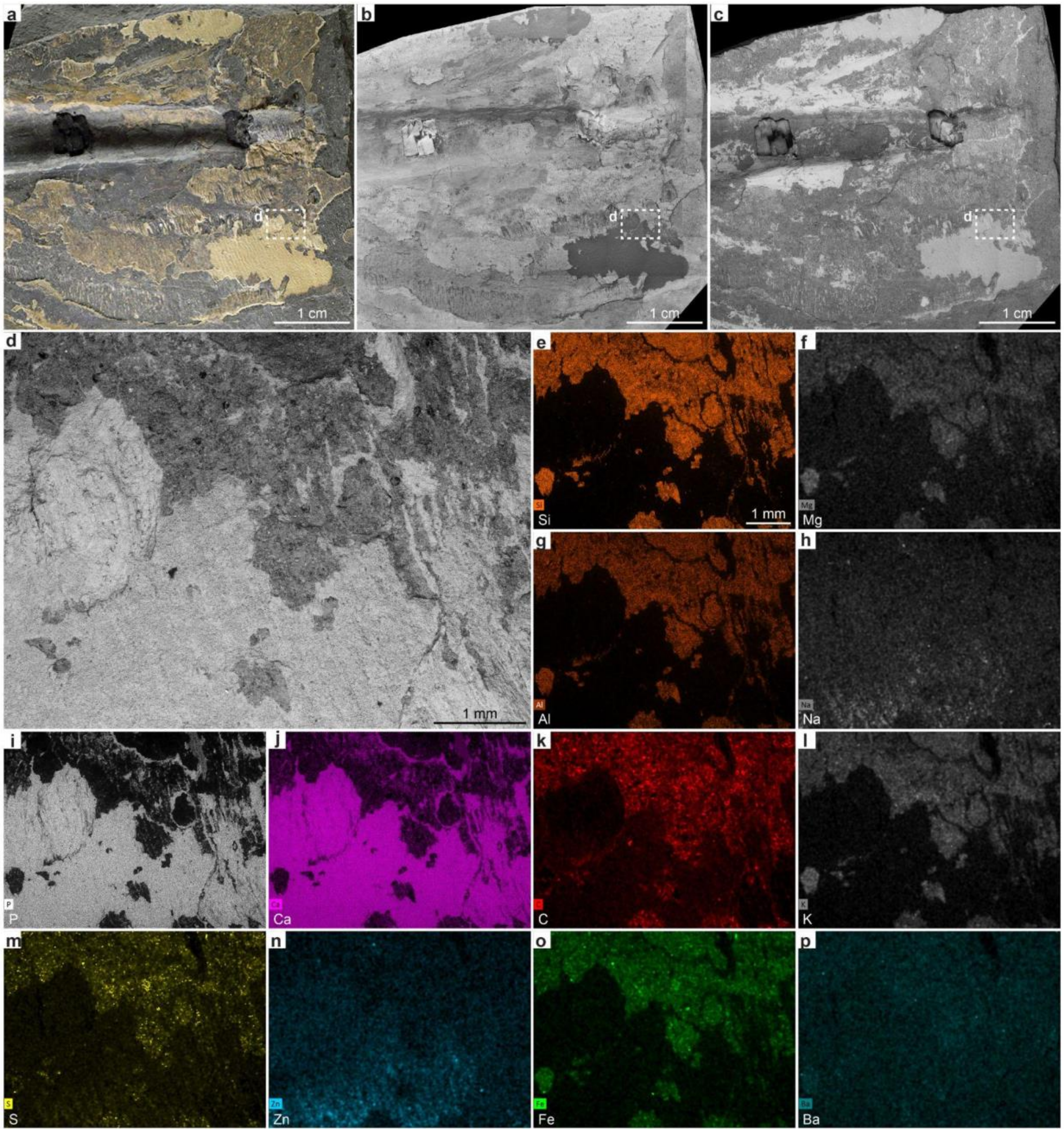

Supplementary Figure S73. Vampyropod *Loligosepia* sp. gladius (NPL00036035.000) with pieces of ink sac from the Posidonia Shale Lagerstätte (Holzmaden, Germany). **a**, Reflected-light photograph of the specimen. **b**, Mosaic SE-SEM image of the specimen. **c-d**, BSE-SEM images. **c**, Mosaic image of specimen. **d**, Magnified view of boxes in **a-c**, showing phosphatized material of gladius. **e-p**, EDS elemental maps of **d**.

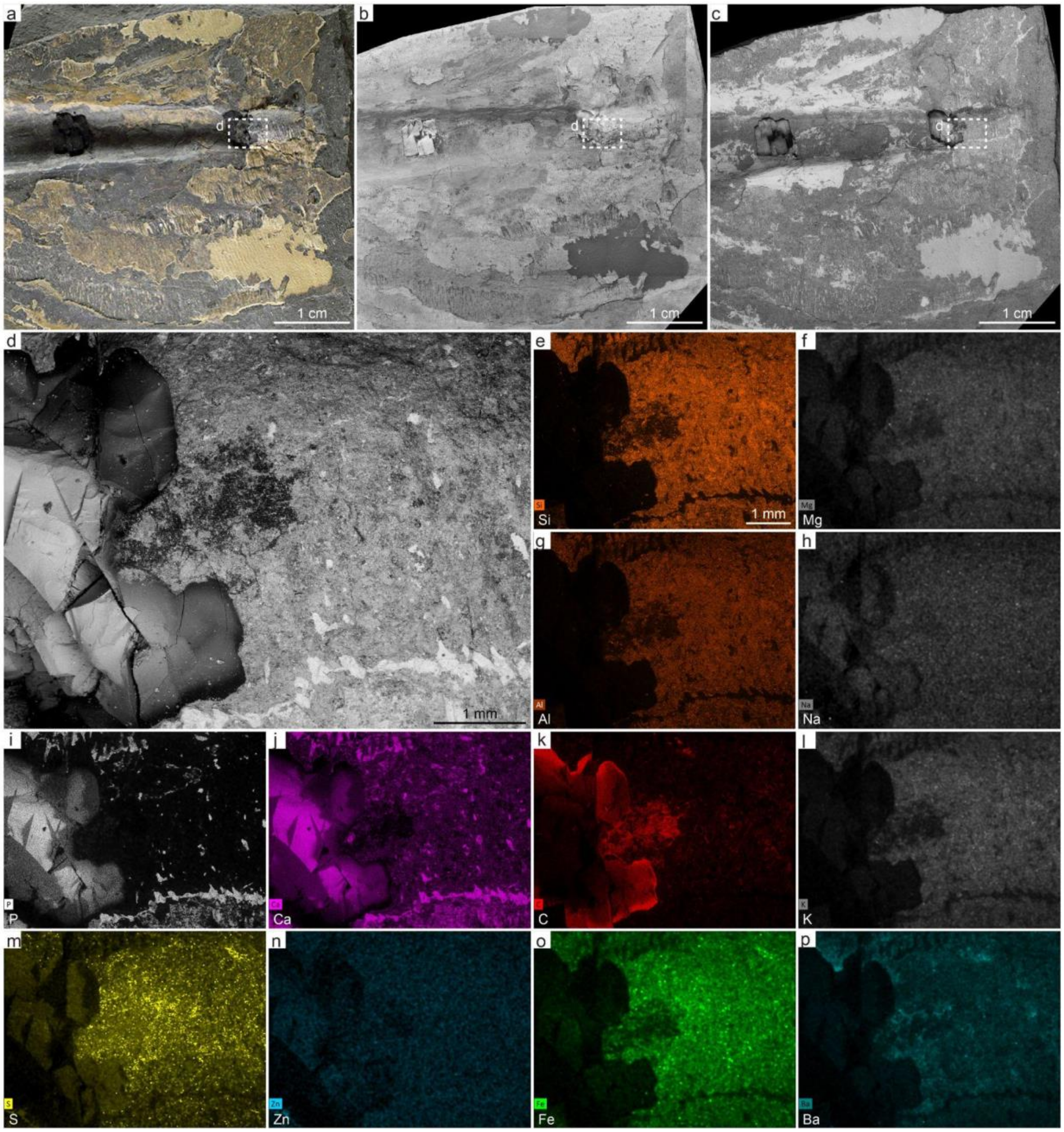

Supplementary Figure S74. Vampyropod *Loligosepia* sp. gladius (NPL00036035.000) with pieces of ink sac from the Posidonia Shale Lagerstätte (Holzmaden, Germany). **a**, Reflected-light photograph of the specimen. **b**, Mosaic SE-SEM image of the specimen. **c-d**, BSE-SEM images. **c**, Mosaic image of the specimen. **d**, Magnified view of boxes in **a-c**, showing phosphatized material of ink sac. **e-p**, EDS elemental maps of **d**.

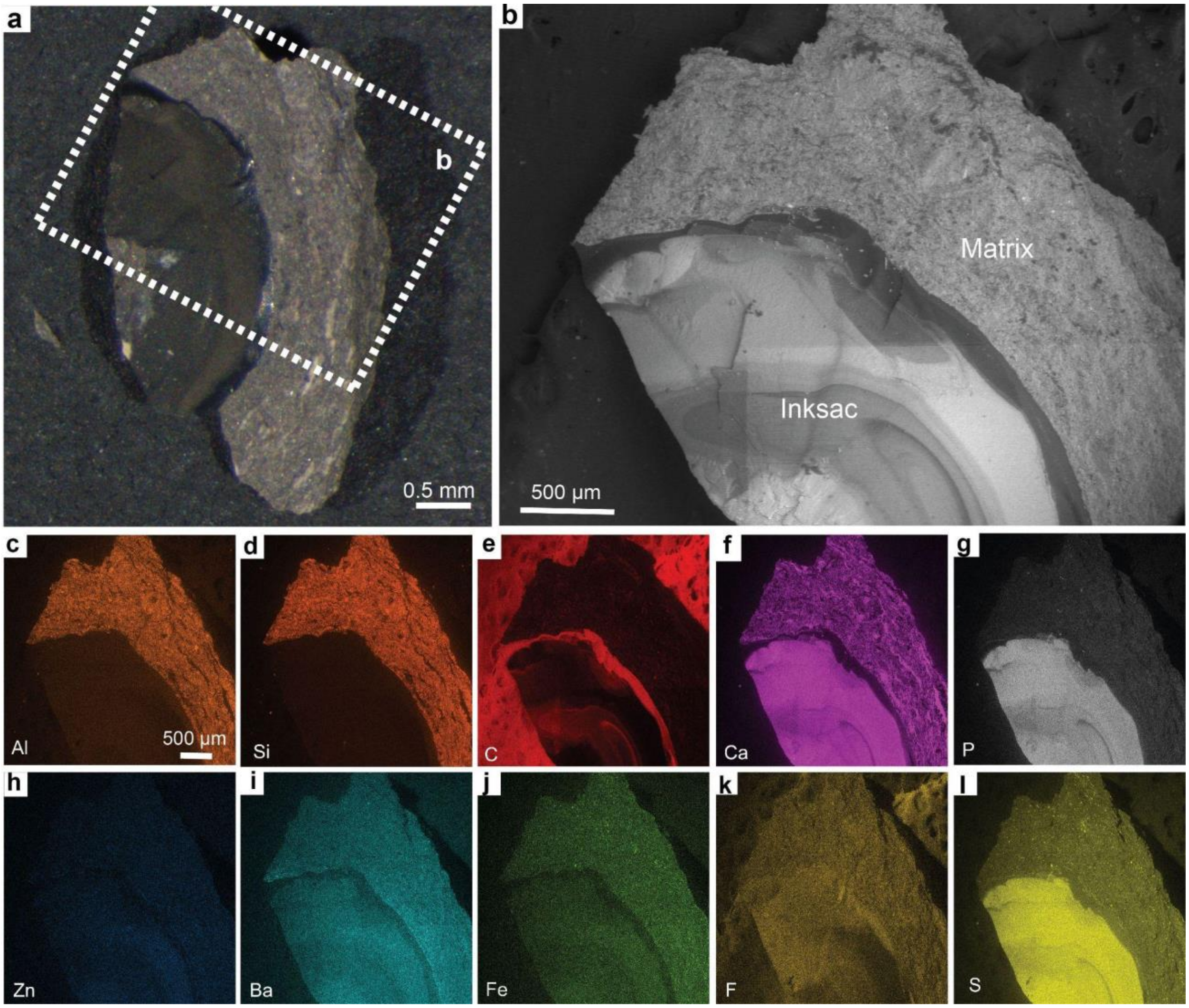

Supplementary Figure S75. Vampyropod ink sac (NPL00036062.000) from the Posidonia Shale Lagerstätte (Dormettingen near Dotternhausen, Germany). **a**, Reflected-light photograph of the specimen. **b**, BSE-SEM images of the box in **a**. **c-l**, EDS elemental maps of **b** showing the sulfur-rich phosphatized ink sac.

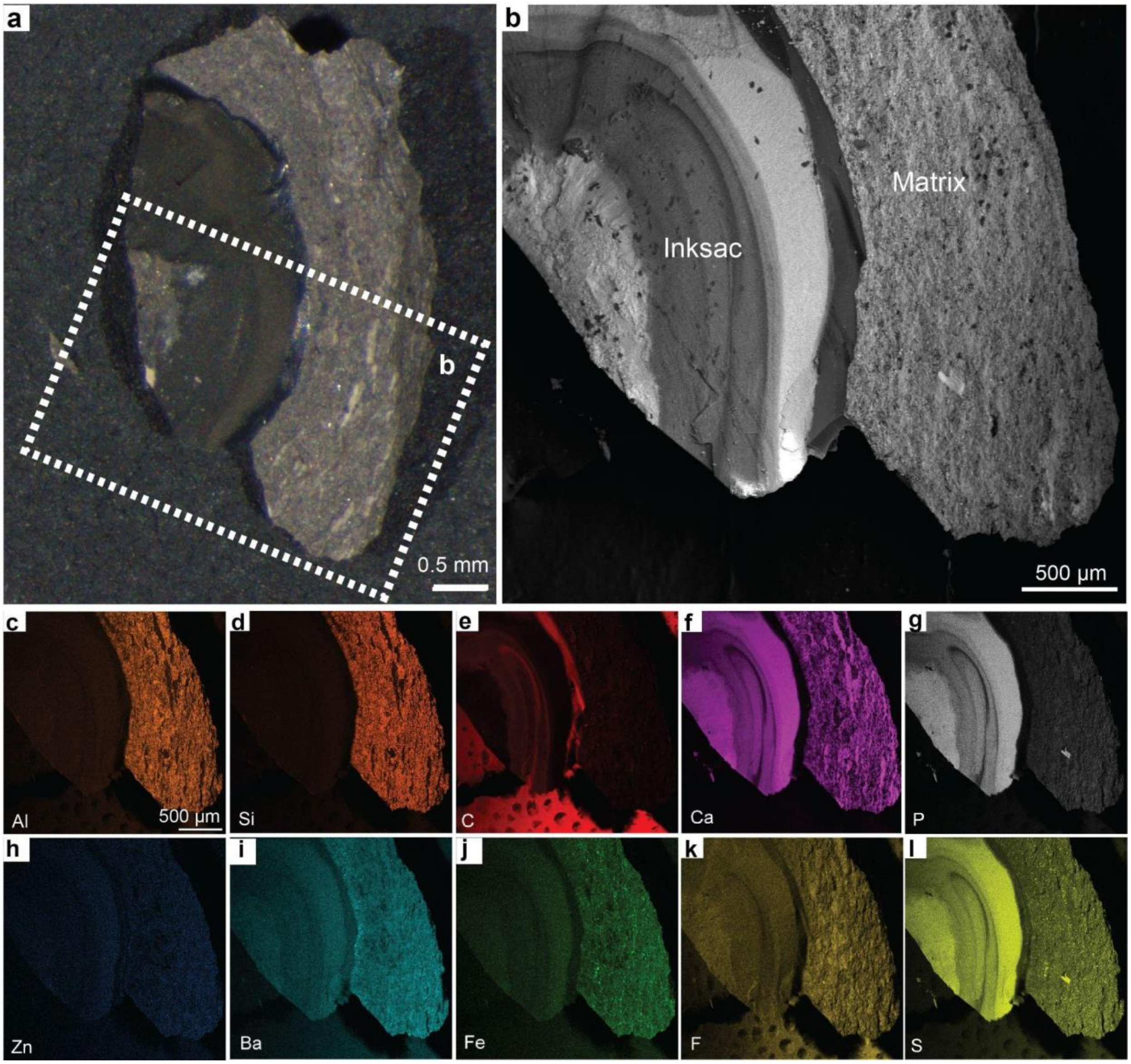

Supplementary Figure S76. Vampyropod (NPL00036062.000) ink sac from the Posidonia Shale Lagerstätte (Dormettingen near Dotternhausen, Germany). **a**, Reflected-light photograph of the specimen. **b**, BSE-SEM images of the box in **a**. **c-l**, EDS elemental maps of **b** showing the sulfur-rich phosphatized ink sac.

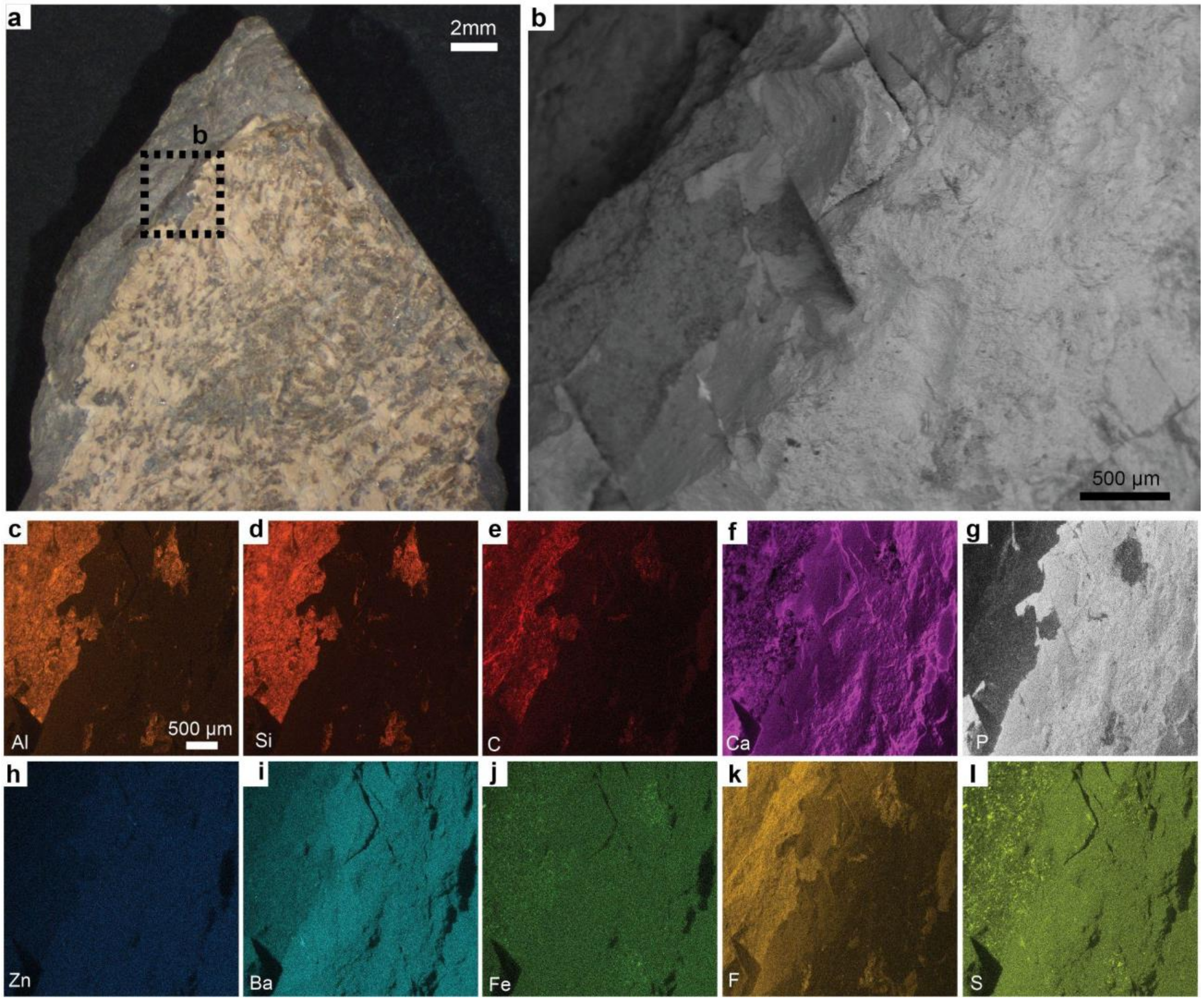

Supplementary Figure S77. Vampyropod (NPL00036062.000) mantle tissue from the Posidonia Shale Lagerstätte (Dormettingen near Dotternhausen, Germany). **a**, Reflected-light photograph of the specimen. **b**, BSE-SEM images of the box in **a**. **c-l**, EDS elemental maps of **b** showing phosphatized mantle tissue.

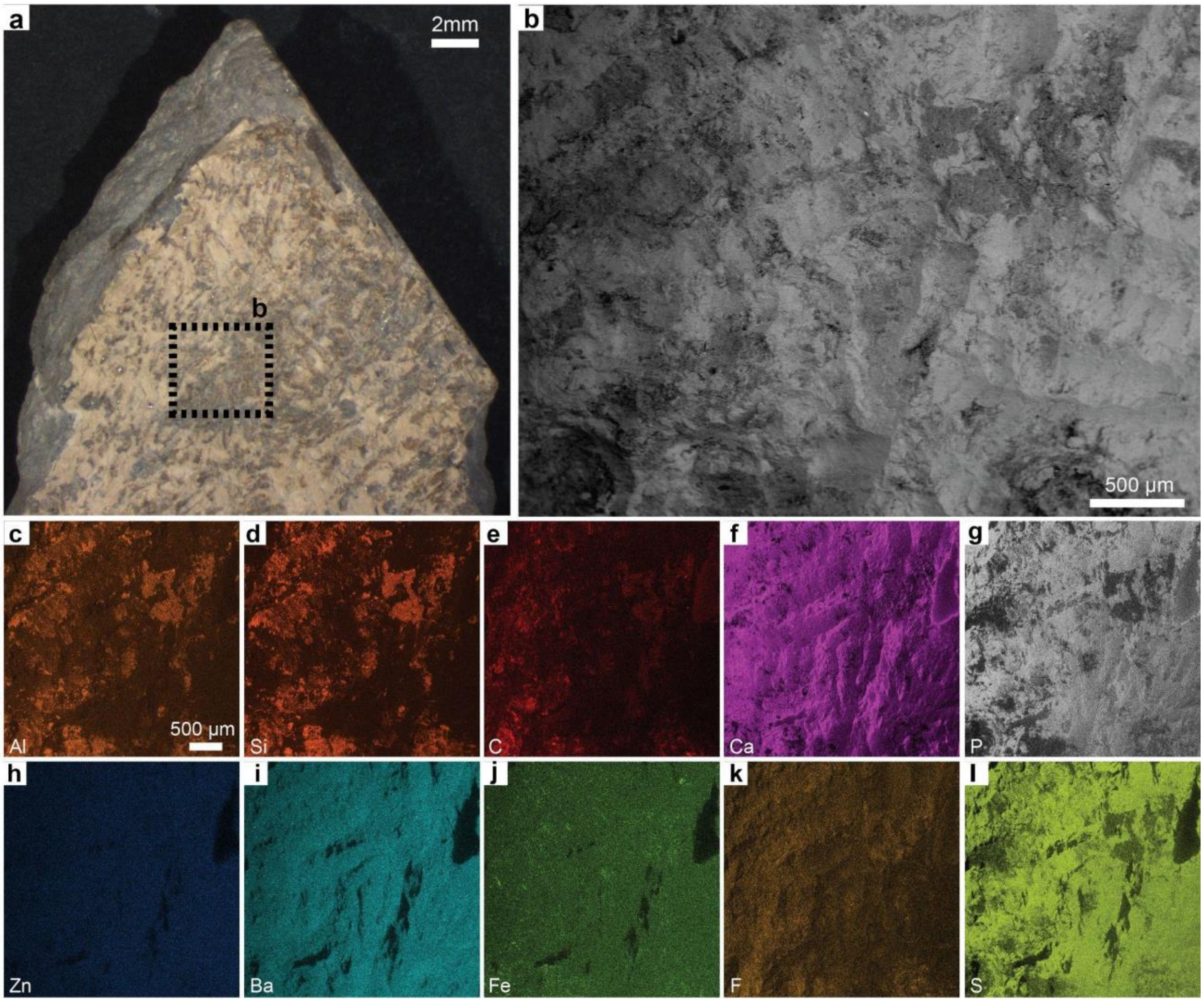

Supplementary Figure S78. Vampyropod (NPL00036062.000) mantle tissue from the Posidonia Shale Lagerstätte (Dormettingen near Dotternhausen, Germany). **a**, Reflected-light photograph of the specimen. **b**, BSE-SEM images of the box in **a**. **c-l**, EDS elemental maps of **b** showing phosphatized mantle tissue.

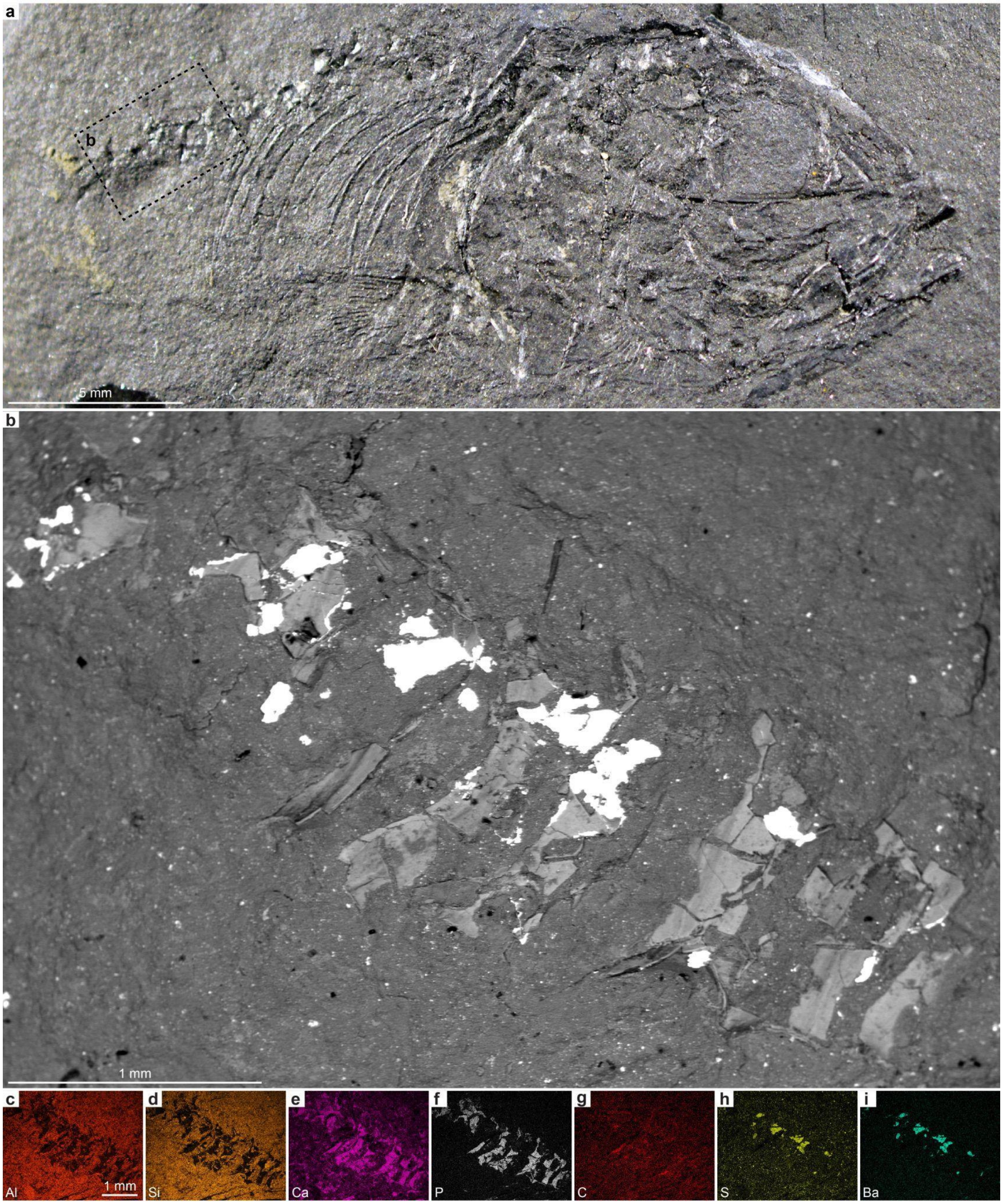

Supplementary Figure S79. Articulated fish with fins and gill arches from Ya Ha Tinda Lagerstätte (TMP2014.021.0043; Toarcian, 16 m in section, Poker Chip Shale Member, Fernie Formation, East Tributary, Alberta, Canada). **a**, Reflected-light photograph of the specimen. **b**, Magnified BSE-SEM image of box in **a**, showing phosphatic skeletal material encrusted by barite. **c-i**, EDS elemental map of **b**.

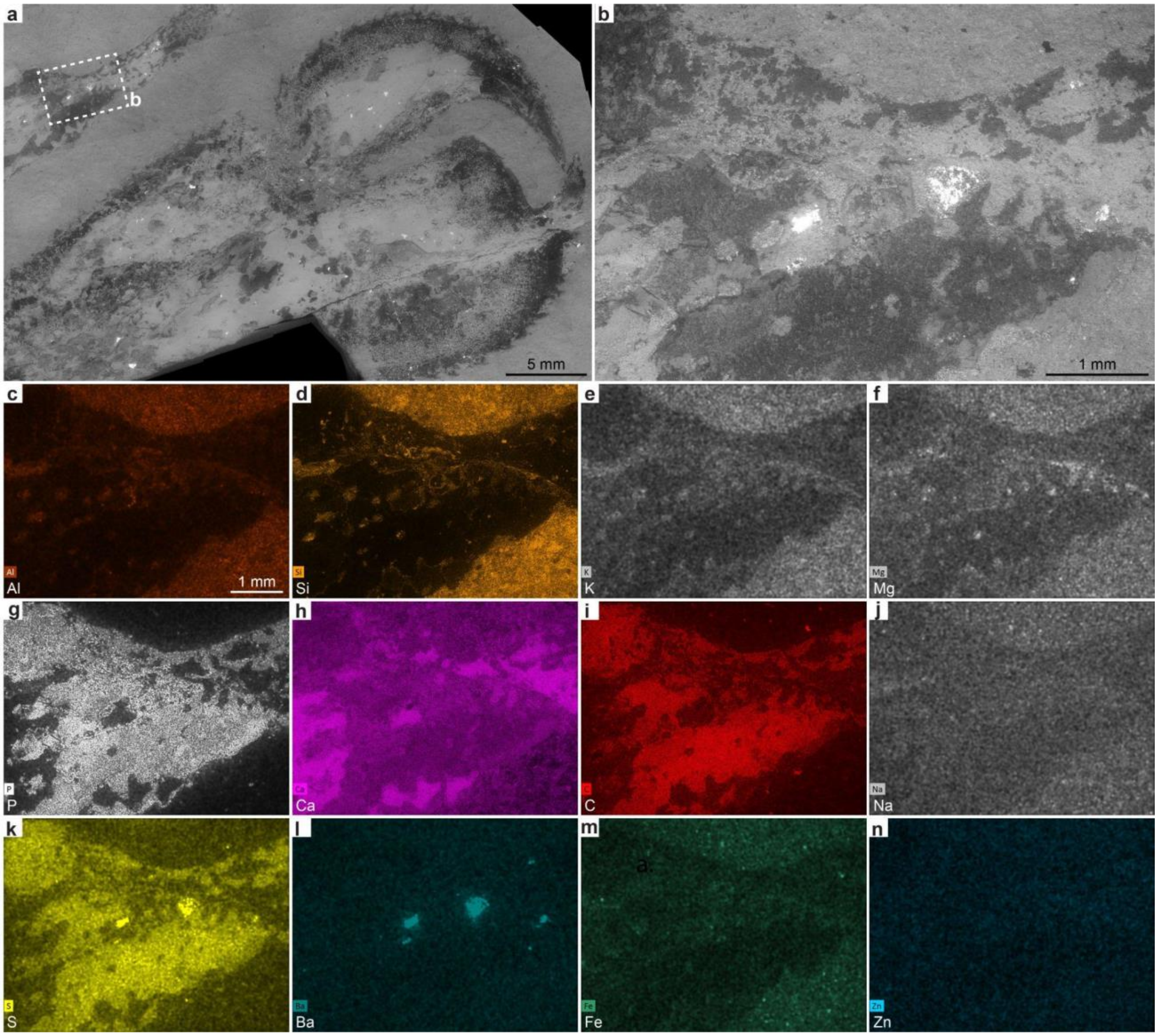

Supplementary Figure S80. Claw of the holotype specimen of the decapod crustacean *Uncina pacifica* from Ya Ha Tinda Lagerstätte (TMP2002.043.0005; *margaritatus* zone, Pliensbachian, Red Deer Member, Fernie Formation, East Tributary, Alberta, Canada). **a**, Mosaic BSE-SEM image of specimen. **b**, Magnified BSE-SEM image of box in **a**, showing phosphatic and carbonaceous material making up the fossil, surrounded by matrix. **c-n**, EDS elemental maps of **b**.

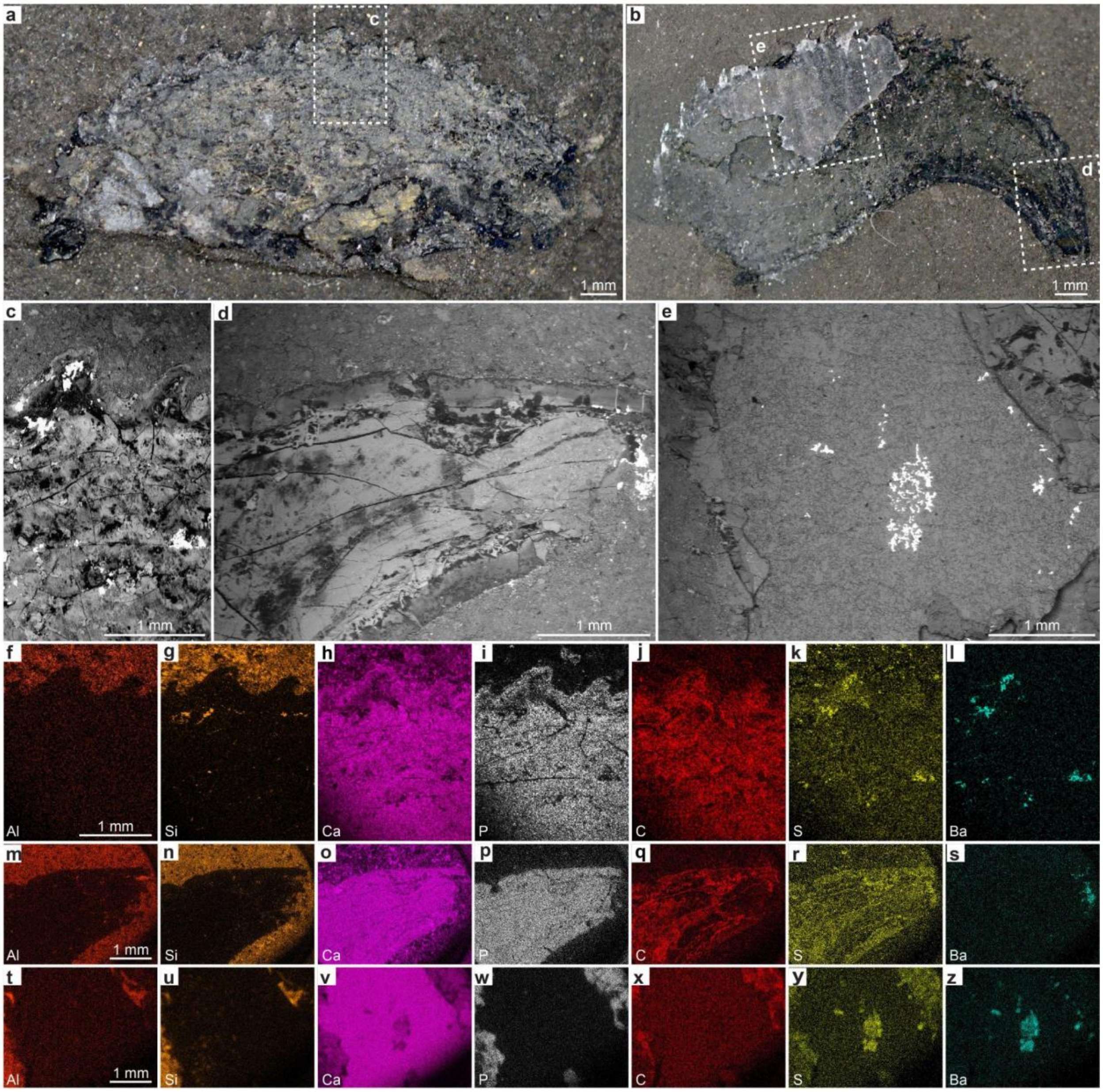

Supplementary Figure S81. Claws of the decapod crustacean *Uncina pacifica* from Ya Ha Tinda Lagerstätte (Red Deer Member, Fernie Formation, East Tributary, Alberta, Canada). **a**, Reflected-light photograph (TMP2018.024.0030). **b**, Reflected-light photograph (TMP2018.024.0037). **c-e**, BSE-SEM images. **c**, Magnified view of box in a, showing the phosphatic and carbonaceous material (encrusted by barite) that make up the fossil. **d**, Magnified view of box in **b**, showing the phosphatic and carbonaceous material (encrusted by barite) that make up the fossil. **e**, Magnified view of box in b, showing calcite and barite that encrust the phosphatic and carbonaceous materials of the fossil. **f-l**, EDS elemental maps of **c**. **m-s**, EDS elemental maps of **d**. **t-z**, EDS elemental maps of e.

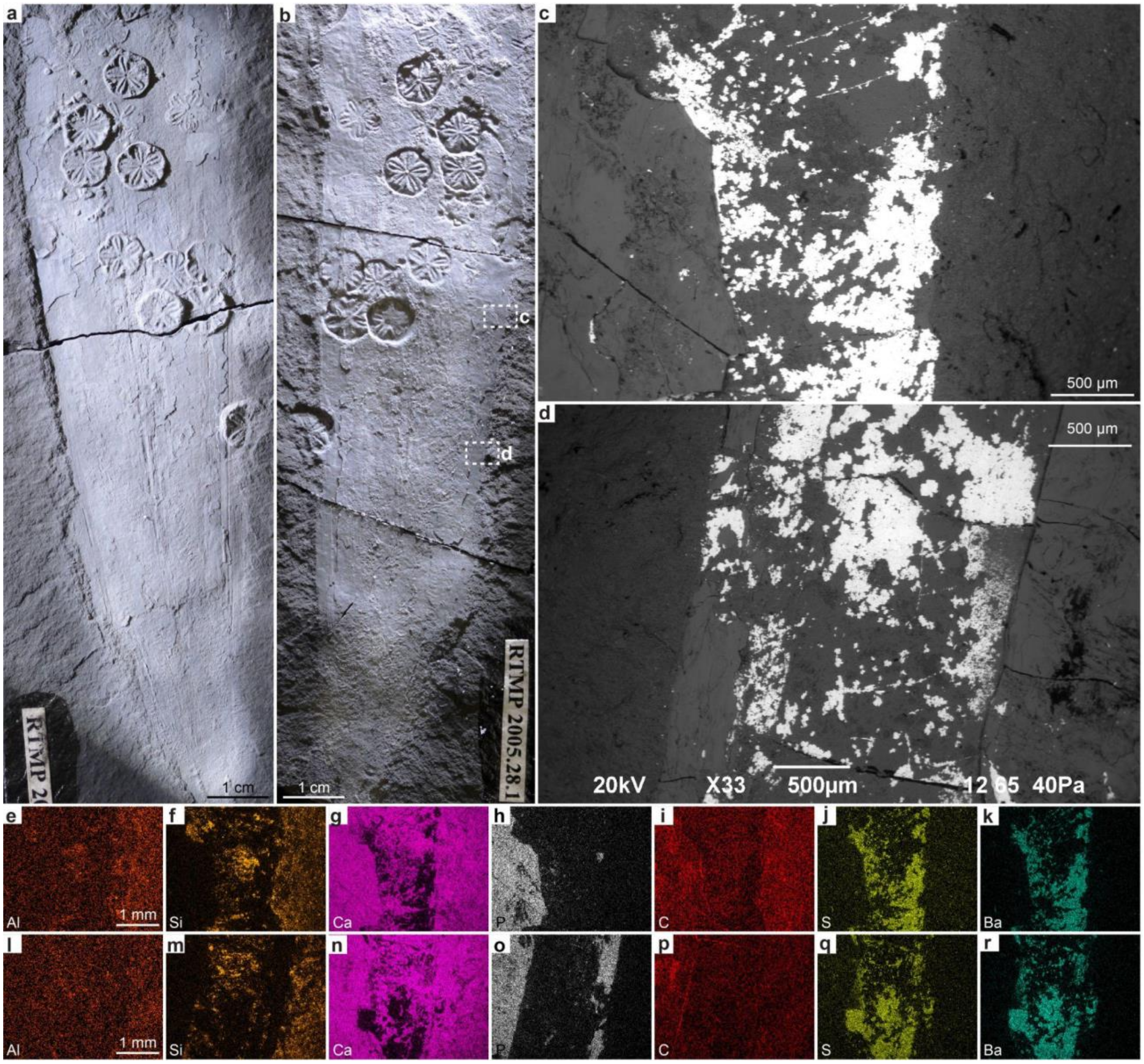

Supplementary Figure S82. Gladius of the coleoid *Paraplesioteuthis* cf. *sagittate* (with several *Seirocrinus* ossicles) from Ya Ha Tinda Lagerstätte (TMP2005.028.0001; *margaritatus* Zone, Pliensbachian, Red Deer Member, Fernie Formation, East Tributary, Alberta, Canada). **a-b**, Reflected-light photographs of part (**a**) and counterpart (**b**). **c-d**, BSE-SEM images. **c**, Magnified view of box in **b**, showing phosphatized gladius tissue, encrusted by calcite and barite, on top of the shale matrix. **d**, Magnified view of box in **b**, showing phosphatized gladius tissue, encrusted by calcite and barite, on top of the shale matrix. **e-k**, EDS elemental maps of **c**. **l-r**. EDS elemental maps of **d**.

Supplementary Table S1. A list of specimens illustrated with the SEM and EDS data in this study

Lagerstätte	Specimen	Specimen number	Figure/ supplementary figure number(s)	Description of specimen tissue	Source location	Source stratigraphy	Curated at
Strawberry Bank	Fish (*Leptolepis*)	BRLSI.M1261	4a, 5, S1, S2	Eye and gut area	Ilminster, UK	Beacon Limestone Fm. (*falciferum* ammonite zone)	Bath Royal Literary and Scientific Institution, Bath, United Kingdom
Strawberry Bank	Fish (*Leptolepis*)	BRLSI.M1261 A	4b, S3, S4, S5	Bones, eyes, gut	Ilminster, UK	Beacon Limestone Fm. (*falciferum* ammonite zone)	Bath Royal Literary and Scientific Institution, Bath, United Kingdom
Strawberry Bank	Fish *(Leptolepis)*	BRLSI.M1269 A	4c, S6, S7, S8	Bones, eyes, gut, tail	Ilminster, UK	Beacon Limestone Fm. (*falciferum* ammonite zone)	Bath Royal Literary and Scientific Institution, Bath, United Kingdom
Strawberry Bank	Fish *(Leptolepis)*	BRLSI.M1271 A	4d, S9, S10, S11	Bones, skull, gut, tail	Ilminster, UK	Beacon Limestone Fm. (*falciferum* ammonite zone)	Bath Royal Literary and Scientific Institution, Bath, United Kingdom
Strawberry Bank	Fish (unidentified)	BRLSI.M1275	4e, S12, S13, S14	Bones, gut, tail	Ilminster, UK	Beacon Limestone Fm. (*falciferum* ammonite zone)	Bath Royal Literary and Scientific Institution, Bath, United Kingdom
Strawberry Bank	Fish *(Leptolepis)*	BRLSI.M1269	4f, S15, S16, S17, S18	Bones, eyes, gut, tail	Ilminster, UK	Beacon Limestone Fm. (*falciferum* ammonite zone)	Bath Royal Literary and Scientific Institution, Bath, United Kingdom
Strawberry Bank	Crustacea (Malacostraca: Proeryon)	BRLSI.M1242	4h, S19	Exoskeleton- claw (cheliped)	Ilminster, UK	Beacon Limestone Fm. (*falciferum* ammonite zone)	Bath Royal Literary and Scientific Institution, Bath, United Kingdom
Strawberry Bank	Crustacea (Malacostraca)	BRLSI.M1243 A	S20, S21, S22	Exoskeleton- claw (cheliped)	Ilminster, UK	Beacon Limestone Fm. (*falciferum* ammonite zone)	Bath Royal Literary and Scientific Institution, Bath, United Kingdom
Strawberry Bank	Crustacea (Malacostraca)	BRLSI.M1243 C	4i, S23, S24, S25, S26	Exoskeleton- claw (cheliped)	Ilminster, UK	Beacon Limestone Fm. (*falciferum* ammonite zone)	Bath Royal Literary and Scientific Institution, Bath, United Kingdom
Strawberry Bank	Crustacea (Malacostraca)	BRLSI.M1245	S27, S28, S29, S30	Exoskeleton- claw (cheliped)	Ilminster, UK	Beacon Limestone Fm. (*falciferum* ammonite zone)	Bath Royal Literary and Scientific Institution, Bath, United Kingdom
Strawberry Bank	Crustacea (Malacostraca)	BRLSI.M1256	4g, 5, S31, S32, S33	Exoskeleton- abdomen (tail)	Ilminster, UK	Beacon Limestone Fm. (*falciferum* ammonite zone)	Bath Royal Literary and Scientific Institution, Bath, United Kingdom
Strawberry Bank	Coleoidea (Vampyropoda)	BRLSI.M3917	4k, 5, S34, S35, S36, S37	Gladii- ink sac and mantle muscle	Ilminster, UK	Beacon Limestone Fm. (*falciferum* ammonite zone)	Bath Royal Literary and Scientific Institution, Bath, United Kingdom
Strawberry Bank	Coleoidea (Vampyropoda)	BRLSI.M1237 B	4j, S38, S39	Gladii	Ilminster, UK	Beacon Limestone Fm. (*falciferum* ammonite zone)	Bath Royal Literary and Scientific Institution, Bath, United Kingdom

Posidonia Shale	Fish (*Leptolepis?*)	NPL00036036 .000	4l, 5, S40, S41, S42, S43	Bones	Kromer Quarry near Ohmden, Germany	Posidonienschiefer Formation	Non-vertebrate Paleontology Lab, University of Texas at Austin, USA
Posidonia Shale	Fish	NPL00094461 .000	4m, S44	Skull	Dormettingen near Dotternhausen , Germany	Posidonienschiefer Formation	Non-vertebrate Paleontology Lab, University of Texas at Austin, USA
Posidonia Shale	Crustacea (Malacostraca: *Uncina posidoniae*)	NPL00036039 .000	4n, S45, S46, S47	Exoskeleton-claw (cheliped)	Germany	Posidonienschiefer Formation (Fleins layer)	Non-vertebrate Paleontology Lab, University of Texas at Austin, USA
Posidonia Shale	Crustacea (Malacostraca: *Uncina posidoniae*)	NPL00036038 .000	4o, 5, S48, S49	Exoskeleton-claw (cheliped)	Holzmaden, Germany	Posidonienschiefer Formation	Non-vertebrate Paleontology Lab, University of Texas at Austin, USA
Posidonia Shale	Crustacea (Malacostraca: *Uncina posidoniae*) claw	NPL00094459 .000	4q, S50, S51, S52, S53	Exoskeleton-claw (cheliped)	Dormettingen near Dotternhausen , Germany	Posidonienschiefer Formation (Unterer Schiefer layer)	Non-vertebrate Paleontology Lab, University of Texas at Austin, USA
Posidonia Shale	Crustacea (Malacostraca: *Uncina*) claw	NPL00094458 .000	4p, S54, S55, S56	Exoskeleton-claw (cheliped)	Dormettingen near Dotternhausen , Germany	Posidonienschiefer Formation (Unterer Schiefer layer)	Non-vertebrate Paleontology Lab, University of Texas at Austin, USA
Posidonia Shale	Crustacea (Malacostraca: *Uncina posidoniae*) claw	NPL00094457 .000	4r, S57, S58, S59	Exoskeleton-claw (cheliped)	Ohmden, Germany	Posidonienschiefer Formation	Non-vertebrate Paleontology Lab, University of Texas at Austin, USA
Posidonia Shale	Coleoidea (Vampyropoda: *Clarkeiteuthis*)	NPL00094460 .000	4t, S60, S61, S62, S63, S64, S65, S66	Gladius- ink sac	Holzmaden, Germany	Posidonienschiefer Formation (Hoblenzer or Hainzen layer)	Non-vertebrate Paleontology Lab, University of Texas at Austin, USA
Posidonia Shale	Coleoidea (Vampyropoda: *Loligosepia aalensis*)	NPL00036037 .000	4s, 5, S67, S68, S69, S70	Gladius- ink sac, mantle muscle	Ohmden, Germany	Posidonienschiefer Formation	Non-vertebrate Paleontology Lab, University of Texas at Austin, USA
Posidonia Shale	Coleoidea (Vampyropoda: *Loligosepia*)	NPL00036035 .000	4u, S71, S72, S73, S74	Gladius- ink sac, mantle muscle	Holzmaden, Germany	Posidonienschiefer Formation	Non-vertebrate Paleontology Lab, University of Texas at Austin, USA

Posidonia Shale	Coleoidea (Vampyropoda)	NPL00036062 .000	S75, S76, S77, S78	Gladius- ink sac, mantle muscle	Dotternhausen quarry, Germany	Posidonienschiefer Formation	Non-vertebrate Paleontology Lab, University of Texas at Austin, USA
Ya Ha Tinda	Fish	TMP2014.021 .0043	4v, 5, S79	Bones	East Tributary section (Alberta, Canada)	Fernie Formation (Poker Chip Shale Member)	Royal Tyrrell Museum of Palaeontology, Drumheller, Alberta, Canada
Ya Ha Tinda	Crustacea (Malacostraca: *Uncina pacifica*)	TMP2002.043 .0005	4z, S80	Exoskeleton-claw (cheliped)	East Tributary section (Alberta, Canada)	Fernie Formation (Red Deer Member)	Royal Tyrrell Museum of Palaeontology, Drumheller, Alberta, Canada
Ya Ha Tinda	Crustacea (Malacostraca: *Uncina pacifica*)	TMP2018.024 .0030	4w, 5, S81	Exoskeleton-claw (cheliped)	East Tributary section (Alberta, Canada)	Fernie Formation (Red Deer Member)	Royal Tyrrell Museum of Palaeontology, Drumheller, Alberta, Canada
Ya Ha Tinda	Crustacea (Malacostraca: *Uncina pacifica*)	TMP2018.024 .0037	4x, S81	Exoskeleton-claw (cheliped)	East Tributary section (Alberta, Canada)	Fernie Formation (Red Deer Member)	Royal Tyrrell Museum of Palaeontology, Drumheller, Alberta, Canada
Ya Ha Tinda	Coleoidea *Paraplesioteuthis* cf. *sagittate*	TMP2005.028 .0001	4y, 5, S82	Gladius	East Tributary section (Alberta, Canada)	Fernie Formation (Red Deer Member)	Royal Tyrrell Museum of Palaeontology, Drumheller, Alberta, Canada

References

1. Orr, P. J., Kearns, S. L. & Briggs, D. E. G. Backscattered electron imaging of fossils exceptionally-preserved as organic compressions. *Palaios* **17**, 110–117 (2002).
2. Orr, P. J., Kearns, S. L. & Briggs, D. E. G. Elemental mapping of exceptionally preserved 'carbonaceous compression' fossils. *Palaeogeography, Palaeoclimatology, Palaeoecology* **277**, 1–8 (2009).
3. Muscente, A. D. & Xiao, S. Resolving three-dimensional and subsurficial features of carbonaceous compressions and shelly fossils using backscattered electron scanning electron microscopy (BSE-SEM). *Palaios* **30**, 462–481 (2015).
4. Briggs, D. E. G. The role of decay and mineralization in the preservation of soft-bodied fossils. *Annual Review of Earth and Planetary Sciences* **31**, 275–301 (2003).
5. Muscente, A. D. *et al.* Exceptionally preserved fossil assemblages through geologic time and space. *Gondwana Research* **48**, 164–188 (2017).
